# Discovery of High-Affinity Amyloid Ligands Using a Ligand-Based Virtual Screening Pipeline

**DOI:** 10.1021/jacs.3c03749

**Published:** 2023-07-13

**Authors:** Timothy S. Chisholm, Mark Mackey, Christopher A. Hunter

**Affiliations:** † Yusuf Hamied Department of Chemistry, University of Cambridge, Lensfield Road, Cambridge CB2 1EW, U.K.; ‡ Cresset, New Cambridge House, Bassingbourn Road, Litlington SG8 0SS, Cambridgeshire, U.K.

## Abstract

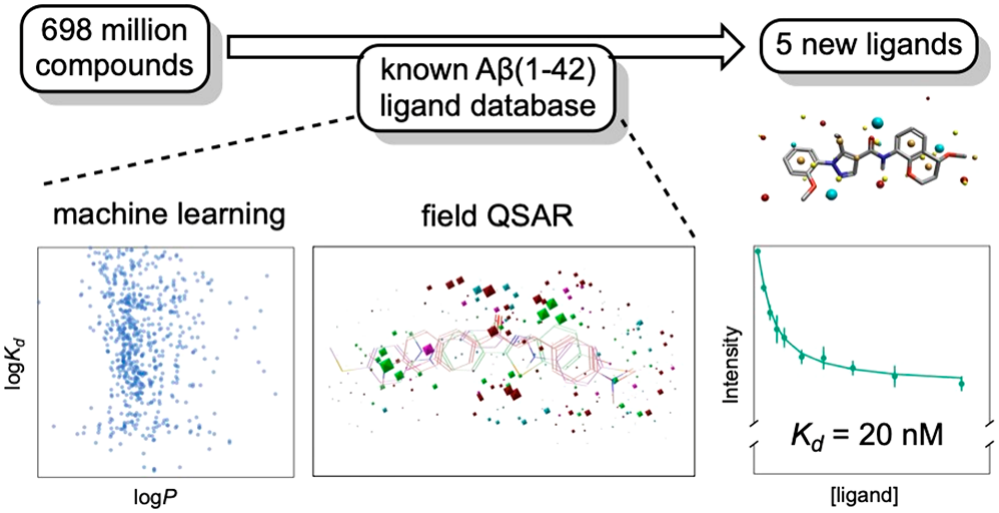

Fibrillar protein aggregates are characteristic of neurodegenerative diseases but represent difficult targets for ligand design, because limited structural information about the binding sites is available. Ligand-based virtual screening has been used to develop a computational method for the selection of new ligands for Aβ(1–42) fibrils, and five new ligands have been experimentally confirmed as nanomolar affinity binders. A database of ligands for Aβ(1–42) fibrils was assembled from the literature and used to train models for the prediction of dissociation constants based on chemical structure. The virtual screening pipeline consists of three steps: a molecular property filter based on charge, molecular weight, and log*P*; a machine learning model based on simple chemical descriptors; and machine learning models that use field points as a 3D description of shape and surface properties in the Forge software. The three-step pipeline was used to virtually screen 698 million compounds from the ZINC15 database. From the top 100 compounds with the highest predicted affinities, 46 compounds were experimentally investigated by using a thioflavin T fluorescence displacement assay. Five new Aβ(1–42) ligands with dissociation constants in the range 20–600 nM and novel structures were identified, demonstrating the power of this ligand-based approach for discovering new structurally unique, high-affinity amyloid ligands. The experimental hit rate using this virtual screening approach was 10.9%.

## Introduction

Amyloidogenic proteins are a class of biomolecules that self-assemble into fibrillar structures with a cross-β sheet structure. The formation of insoluble protein aggregates from amyloidogenic proteins is a hallmark of many diseases, most prominently neurodegenerative diseases.^1−3^ The most common neurodegenerative disease, Alzheimer’s disease (AD), is characterized by the deposition of amyloid plaques comprised of misfolded amyloid-β (Aβ) peptide and neurofibrillary tangles (NFTs) comprised of misfolded tau protein.^4,5^ While the 40-residue Aβ peptide, Aβ(1–40), is the most abundant isoform in the brain, the 42-residue Aβ peptide, Aβ(1–42), is the primary component of amyloid plaques found in AD.^6^ These peptides are generated by cleavage of the integral membrane protein amyloid precursor protein (APP).^7^ However, the precise physiological role of APP and Aβ peptides, and their role in disease onset and progression, is poorly understood.^8,9^

The only current method to definitively diagnose AD is through the post-mortem histopathological identification of Aβ plaques.^10^ Due to the limited accessibility of living brains, AD is diagnosed in the clinic using cognitive tests alongside a panel of imaging or biofluid tests.^11^ However, these diagnostic methods do not reliably diagnose AD until extensive neuronal damage has already occurred.^10^ Accurate and early diagnosis is essential to ensure that patients receive appropriate disease management and to accurately recruit clinical trial populations when developing disease therapeutics.^12^ One promising diagnostic strategy is the detection of amyloid plaques *in vivo* using positron emission tomography.^13−17^ Several radiolabeled PET probes have been reported in the literature for imaging amyloid plaques.^18−21^ Some PET probes have been approved for clinical use, but show insufficient sensitivity and specificity to definitively diagnose AD by themselves.^17,22,23^ Amyloid ligands are also useful for *ex vivo* applications including for imaging and characterizing amyloid deposits and for monitoring protein aggregation.^24,25^ For these applications, amyloid ligands that bind selectively to target fibrils with a high affinity are needed.

To date, amyloid ligands have primarily been discovered from high-throughput screening efforts combined with structure–activity relationship (SAR) studies. These approaches have identified new structural classes of amyloid ligands and have generated high-affinity binders, yet are resource and time intensive. Virtual screening (VS) is one method that can improve the efficiency of discovering active ligands.^26−28^ VS is a computational technique that aims to identify active molecules by using knowledge about either the target (structure-based VS) or known active molecules (ligand-based VS).^29,30^ Structure-based VS typically requires high-resolution structures of the target binding site. Structural information has only recently become available for amyloid fibrils with advancements in cryo-EM technology.^31,32^ However, the binding of amyloid ligands to fibrils is not a straightforward process: multiple binding sites exist, and direct interactions between multiple ligands can occur.^33−39^ Previous efforts have discovered amyloid-binding ligands from blind docking studies,^40^ although results from this approach do not always reflect experimental results.^41,42^ Structural knowledge of ligand binding sites is therefore required for accurate structure-based VS, and this remains an unsolved problem for amyloid fibrils.

Ligand-based VS requires no knowledge of the target’s structure or binding sites.^43−46^ Instead, structure–activity data of known actives (and inactives) are used to predict binding. Previous studies have developed ligand-based pharmacophores to model the binding of stilbene and flavone analogues to amyloid fibrils.^47,48^ Because of the large quantity of reported binding data for amyloid ligands, a ligand-based VS method for finding novel high-affinity ligands is appealing. Here we describe a three-step pipeline using ligand-based VS methods to identify novel high-affinity amyloid ligands. The approach has successfully identified five new ligands exhibiting nanomolar binding affinities for Aβ(1–42) fibrils.

## Approach

**Figure 1:**
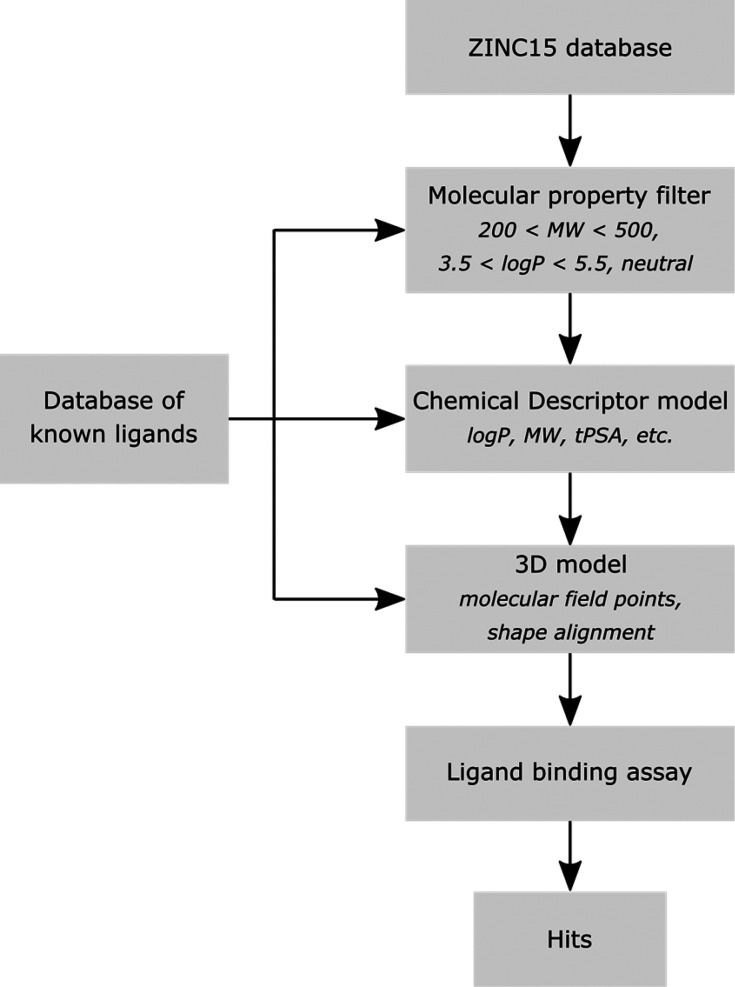
Overview of the three-step ligand-based VS pipeline used. A molecular property filter was applied to the ZINC15 database to select a subset of compounds that are similar to known amyloid ligands. This subset was then screened through two models developed by using the binding affinities of known amyloid ligands in the FBH database. The ligands with the highest predicted affinities obtained from the VS models were then experimentally screened to identify hits.

The steps of the pipeline for the discovery of new amyloid ligands using ligand-based VS are illustrated in Figure 1. First, a database of potential ligands is filtered based on log*P* (the partition coefficient between octan-1-ol and water), molecular weight, and charge. Then machine learning is used to develop a model that describes the dissociation constants of known ligands using multiple chemical descriptors. Finally, a more complicated model that incorporates information about the 3D molecular fields of ligands is trained to predict the dissociation constants of known ligands. In this paper, we describe the development of VS models based on a data set of known Aβ(1–42) ligands and the application of these models to screen 698 million compounds from the ZINC15 database.^49^ A structurally diverse subset of the most promising leads identified by the ligand-based VS was experimentally assayed for binding to Aβ(1–42) fibrils to discover a number of new amyloid ligands (hits).

## Results and Discussion

### Ligand Database

**Figure 2:**
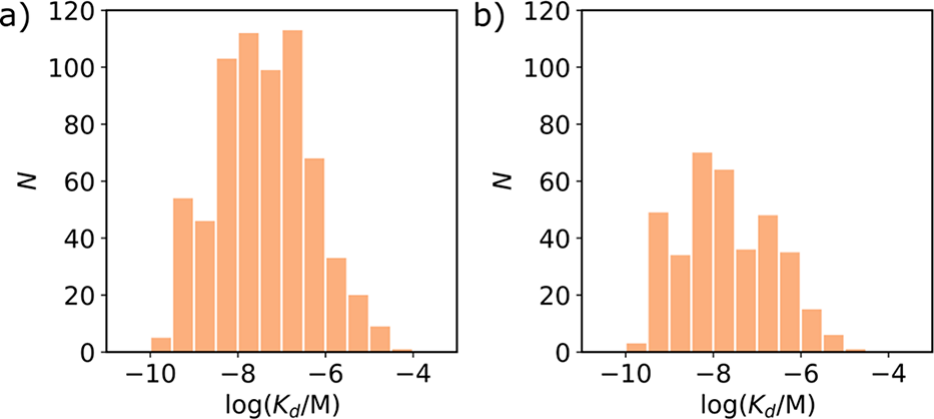
Frequency distributions showing the number of ligands (*N*) with experimentally determined *K*_d_ values for binding to (a) Aβ(1–42) fibrils and (b) the FBH site on Aβ(1–42) fibrils. Where multiple *K*_d_ values were reported for a single ligand, the average value was used.

We first compiled a data set of experimentally determined dissociation constants for ligand binding to Aβ(1–42) fibrils.^50−183^ This initial data set contained a total of 707 unique ligands, which were structurally diverse and exhibited micromolar to subnanomolar dissociation constants (*K*_d_) (Table S3, Table S4). Of these ligands, 44 had *K*_d_ values reported as limiting values (e.g., *K*_d_ > 1 μM). Figure 2a illustrates the distribution of dissociation constants measured for the remaining 663 ligands. Many of these ligands have been reported to target different binding sites on Aβ(1–42) fibrils,^33−38,50^ and in order to construct an accurate predictive model for ligand-based VS, only ligands that share a common binding site should be used.

**Figure 3:**
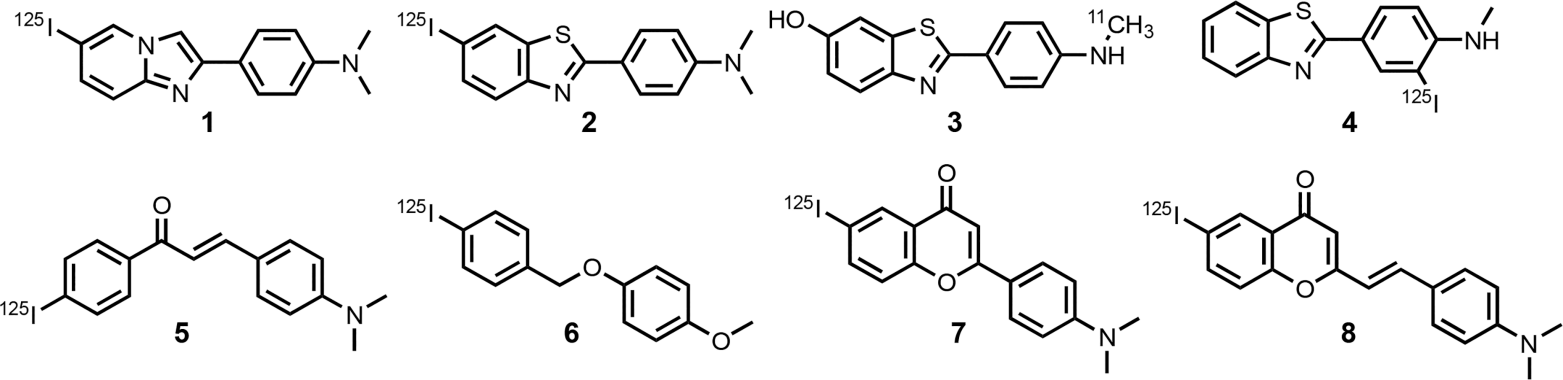
Structures of radiolabeled ligands used in competition assays to report on the FBH binding site on Aβ(1–42) fibrils.

The best way to identify ligands that bind to the same site is from competition assays, and the most common competition assay used to study binding to Aβ(1–42) fibrils involves the displacement of radiolabeled fused 6,5-benzoheterocycles (FBH) (see ligands **1**–**4** in Figure 3). The dissociation constants measured for ligands **1**–**4** in direct binding assays are the same as the dissociation constants measured by competition assays using any pair of these four ligands.^51−66^ This result indicates that ligands **1**–**4** all bind at the same sites on the fibrils, which we designate the FBH site. Similarly, dissociation constants measured for ligands **5**–**8** in direct binding assays are the same as the dissociation constants measured by competition assays with any of ligands **1**–**4**.^67−71^ We therefore conclude that dissociation constants measured by displacing any of the radiolabeled ligands **1**–**8** in a competition assay must report on binding to the FBH site.

**Figure 4:**
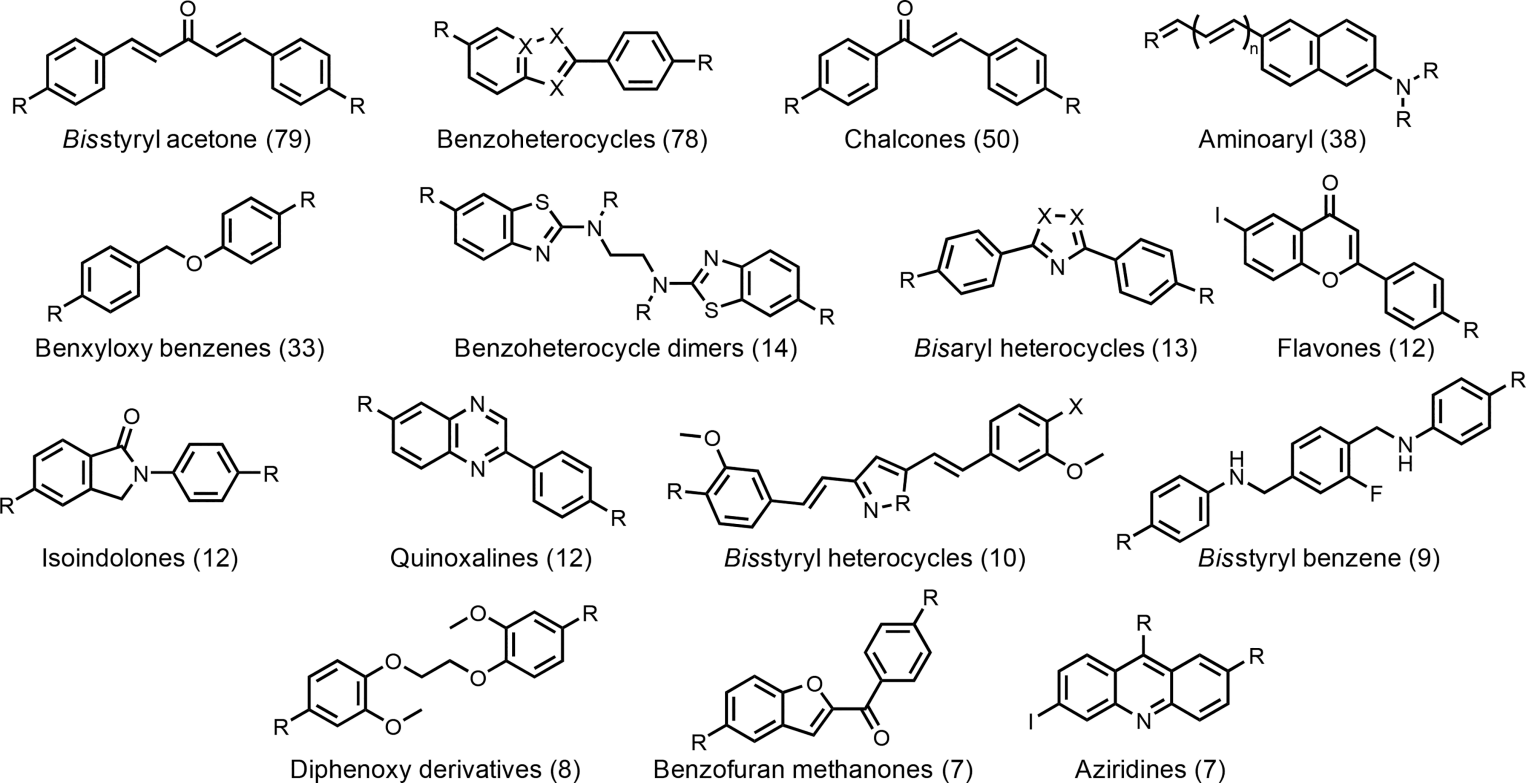
Common ligand motifs that bind to the FBH site on Aβ(1–42) fibrils. The number of unique compounds reported for each structural class is shown in brackets. R and X represent sites of structural variation.

Previous work indicates that there are high-affinity and low-affinity FBH binding sites on Aβ(1–42) fibrils, but the competition assays involving displacement of **1**–**8** report primarily on the high-affinity site.^53,55,124^ We identified a total of 388 ligands within the Aβ(1–42) data set that had been characterized using a competition assay against one of **1**–**8** (Table S3). Of these ligands, 27 had binding constants reported as limiting values. The remaining 361 ligands exhibit a similar range of dissociation constants to that found in the complete 663 ligand data set (compare Figures 2a and 2b), and they have diverse chemical structures (see Figure 4). The database of 388 FBH site ligands therefore constitutes a good starting point for the ligand-based VS pipeline shown in Figure 1.

### Molecular Property Filter

**Figure 5:**
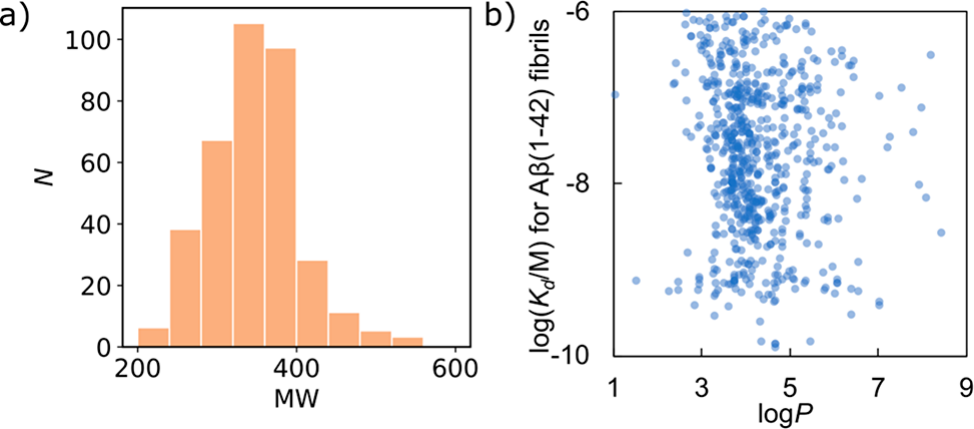
Molecular properties of ligands in the FBH database. (a) Frequency distribution of molecular weights. (b) Relationship between *K*_d_ and log*P* values.

The first step in the pipeline in Figure 1 is to filter the compounds in the ZINC15 database based on molecular properties. Only two of the ligands in the FBH database contain ionizable functional groups, so all charged compounds were excluded. The molecular weights of the ligands in the FBH database fall in the range 200–500 Da, so a filter was applied to exclude any compounds with a molecular weight outside of this window (Figure 5a). Figure 5b shows that although there is no correlation between binding affinity and log*P*, there appears to be a cutoff in log*P* below which there are very few ligands in the database. A third filter was therefore used to exclude compounds with a log*P* outside the range 3.5 to 5.5, reducing the total number of compounds for screening from 698 million to 63 million.

### Chemical Descriptor Model

The second step in the pipeline in Figure 1 is to build models based on chemical descriptors of the ligands in the FBH database. A total of 45 different descriptors based on 1D compositional properties (e.g., molecular weight), 2D topological properties (e.g., topological polar surface area), and 3D conformational properties (e.g., asphericity) were calculated for each ligand using the python package RDKit.^184^ In order to obtain the 3D descriptors, a three-dimensional structure was calculated for each ligand using the XedeX conformation hunting algorithm in Forge.^185^ The Pearson correlation coefficient was less than 0.9 for all pairwise comparisons of the descriptors, which indicates that they can be treated as independent variables. Each set of descriptors *X* was standardized using eq 1 in order to center the distributions at zero with unit standard deviation.
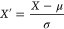
1where μ and σ are the mean and standard deviation of descriptor *X* across the data set.

Different machine learning methods implemented in SciKit-learn were then used to develop predictive models for the values of log(*K*_*d*_/M) in the FBH database.^186^ Machine learning models based on decision trees and boosted trees as well as a support vector machine were implemented in a nested cross-validation (CV) procedure using *k*-fold validation (*k* = 5 for both inner and outer loops, see SI).^187^ Regression models were scored by calculating the mean average error (MAE) between the predicted and experimental values of log(*K*_d_/M). However, 27 dissociation constants were reported as limiting values and could not be readily incorporated into regression models, so classification models were developed by converting the log(*K*_d_/M) of each ligand into a binding class: class 0 for log(*K*_d_/M) ≤ −8, class 1 for −8 < log(*K*_d_/M) ≤ −7, class 2 for −7 < log(*K*_d_/M) ≤ −6, and class 3 for −6 < log(*K*_d_/M). The classification models were scored by calculating the balanced accuracy. The scores for all of the different models are reported in Table 1.

**Table 1:** Evaluation of Models for Prediction of log(*K*_d_/M) for Ligands in the FBH Database^a^

	classification	regression
model	balanced accuracy	MAE
random forest	0.57	0.48
extra trees	0.43	0.55
XGBoost^188^	0.52	0.51
gradient boosting	0.58	0.48
LightGBM^189^	0.55	0.45
histogram-based GB	0.58	0.47
Ada boost^190^	0.46	0.63
support vector machine	0.60	0.41
baseline (average)		1.10
baseline (randomize)	0.33	2.10

a Scores are the average of the best models for each pass of the 5-fold outer cross-validation procedure.

The classification models gave balanced accuracy scores of 0.43 to 0.60, all of which represent an improvement on a random reallocation baseline model that gave a balanced accuracy of 0.33. The support vector machine was the highest-scoring classification model, with a balanced accuracy of 0.60. However, the improvement in the balanced accuracy score relative to the baseline model is modest, and since classification models do not predict the values for continuous variables such as log(*K*_d_/M), this approach was not considered further.

All of the regression models outperformed a random reallocation baseline model (MAE = 2.10) and a baseline model that used the average value of log(*K*_d_/M) for all ligands (MAE = 1.10). The support vector machine was the highest-scoring regression model, with an MAE of 0.41. The random forest, extra trees, and gradient boosted models also scored relatively well. An advantage of the random forest model is that the relative importance of different chemical descriptors can be evaluated (see SI). This analysis suggested that the number of aromatic rings is the most important feature for determining log(*K*_d_/M), whereas the number of aliphatic rings and aliphatic chains has little influence. Based on the results in Table 1, the regression support vector machine model was selected as the chemical descriptor model for the second step of the pipeline in Figure 1. The 63 million compounds selected from the ZINC15 database using the molecular property filter were predicted to have dissociation constants in the range 4.1 < −log(*K*_d_/M) < 10.2 using the support vector machine model. A total of 10,000 compounds were predicted to have values of −log(*K*_d_/M) > 9.8, and these compounds were selected for further analysis in the next step.

### 3D Model

**Figure 6:**
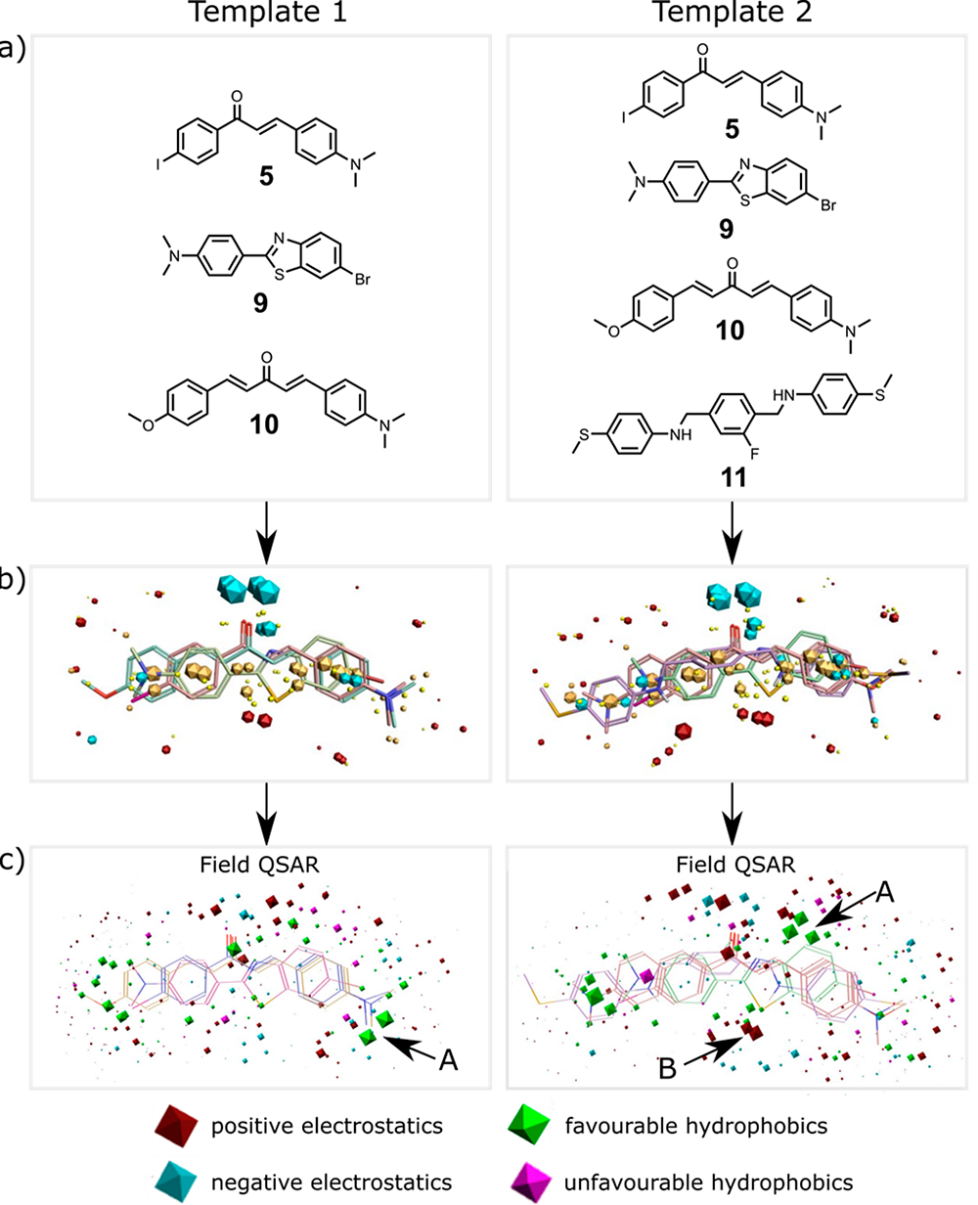
Field point templates. (a) Reference ligands **5**, **9**, and **10** were used to construct Template 1, and **5**, **9**, **10**, and **11** were used to construct Template 2. (b) 3D alignment of the reference ligands showing the field points (blue: negative electrostatic potential; red: positive electrostatic potential; orange: high hydrophobicity; yellow: van der Waals interactions). (c) Field-QSAR models shown relative to the reference ligands: blue sites describe regions where more negative or less positive electrostatic field coefficients favor affinity; red sites describe regions where less negative or more positive electrostatic field coefficients favor affinity; green sites describe regions where hydrophobes favor affinity; pink sites describe regions where hydrophobes disfavor affinity. The sites labeled A represent regions where hydrophobic interactions are important, and the site labeled B highlights important electrostatic interactions.

The third step of the pipeline in Figure 1 is the development of a model using 3D descriptions of the ligands in the FBH database. Cresset field points, which describe the local extrema of the electrostatic, van der Waals, and hydrophobic potential fields, were calculated for a diverse set of conformations of each ligand using the extended electronic distribution (XED) molecular mechanics force field in the Forge software.^185,191^ These field points were used to construct 3D models by using the process outlined in Figure 6. First, a small number of high-affinity reference ligands that have structural cores representative of the entire database were chosen. Field points were used to align multiple conformers of the reference ligands to one another to generate a set of templates. These field point templates were scored based on the shape and field similarity of the aligned ligands, and the best template was selected and used to align all remaining ligands in the database. Finally, a quantitative structure–activity relationship (QSAR) model was generated from the relationship between the experimentally measured log(*K*_d_/M) and the field point distribution of each ligand. This model provides the basis for predicting the affinity of different compounds on a virtual screen.

Ligands **5**, **9**, **10**, and **11** shown in Figure 6a were used to construct templates because they have nanomolar binding affinities and structural cores that occur with a high frequency in the FBH database. It was possible to align all four ligands to create a template, but high-energy conformations were required (Template 2 in Figure 6). If ligand **11** was excluded, it would be possible to align the other three ligands in low-energy conformations (Template 1 in Figure 6). Structurally similar ligands from the FBH database were then aligned to these templates: for Template 1, benzoheterocycles, aminoaryls, flavones, quinoxalines, benzyloxybenzenes, chalcones, bis-aryl heterocycles, and bis-styryl acetones; for Template 2, bis-styryl heterocycles and bis-styryl benzenes were added (see Figure 4). Alignments were scored based on the similarity of the shape and field points of the aligned compound to the template. Alignments were manually reviewed to ensure consistency within each structural class. Certain functionalities proved to be challenging to align. For example, large numbers of alignments with similar scores but different field point distributions were generated by ligands with ethylene glycol chains or polyene linkers. These compounds were discarded from the model development. After refining the aligned data set, a total of 212 ligands were aligned to Template 1 and 222 ligands were aligned to Template 2.

For each Template, the ligands were partitioned into a training set (80%) and a test set (20%) for model development. Partitioning was activity-stratified and performed manually to ensure that each of the ligand structural classes shown in Figure 4 was represented in both training and test sets. Five different QSAR models were constructed for each template using Forge: random forest, support vector machine, relevance vector machine, *k*-nearest neighbors, and a regression method based on partial least-squares analysis of field points (field QSAR).^192^ A *k*-fold cross-validation procedure (*k* = 5) was used for the random forest, support vector machine, and relevance vector machine models, and a leave-one-out cross-validation procedure was used for the *k*-nearest neighbors and field QSAR models. Model performance was evaluated using the regression coefficient *r*^2^ for the training and test sets and the cross-validation regression coefficient *q*^2^ for the training set. The results are listed in Table 2.

**Table 2:** Regression Coefficients (*r*^2^) and Cross-Validation Regression Coefficients (*q*^2^) for the Forge Models

template	model	cross-validation *q*^2^	training set *r*^2^	test set *r*^2^
Template 1	field QSAR	0.53	0.83	0.43
	random forest	0.48	0.93	0.57
	support vector machine	0.50	0.99	0.49
	*k*-nearest neighbors	0.51	a	0.64
	relevance vector machine	0.46	0.86	0.54
Template 2	field QSAR	0.53	0.81	0.38
	random forest	0.43	0.93	0.28
	support vector machine	0.56	0.99	0.50

a Not applicable.

The performances of different models were very similar for the training and cross-validation sets. For the test set, the highest *r*^2^ values were obtained using the random forest model for Template 1 and the support vector machine model for Template 2. These models together with the field QSAR models were used to screen the 10,000 compounds that were selected from the ZINC15 database using chemical descriptors. Although the test set *r*^2^ values for the field QSAR models were lower, these models are useful because they identify the interactions that are important for determining ligand binding affinity. Figure 6c shows the two field QSAR models. The two templates show clear differences in the most important sites identified for hydrophobic (arrow A) and electrostatic (arrow B) interactions.

The 10,000 compounds were first filtered using the field QSAR distance-to-model score, which is based on how well the field points of the compound are represented in the models illustrated in Figure 6c. Only compounds with a good or excellent distance-to-model score were considered further. These compounds were then separately ranked for each template by averaging the values of log(*K*_d_/M) predicted by two models, i.e., random forest and field QSAR for Template 1 and support vector machine and field QSAR for Template 2. The 50 compounds with the lowest average log(*K*_d_/M) for each template were selected, and 46 of these 100 compounds were purchased for experimental screening based on commercial availability and scaffold diversity (see SI). While previously reported Aβ(1–42) ligands in the literature (Figure 4) are generally rigid and flat, many of the hits from the virtual screening procedure contained flexible aliphatic chains and rings.

### Experimental Binding Assays

**Figure 7:**
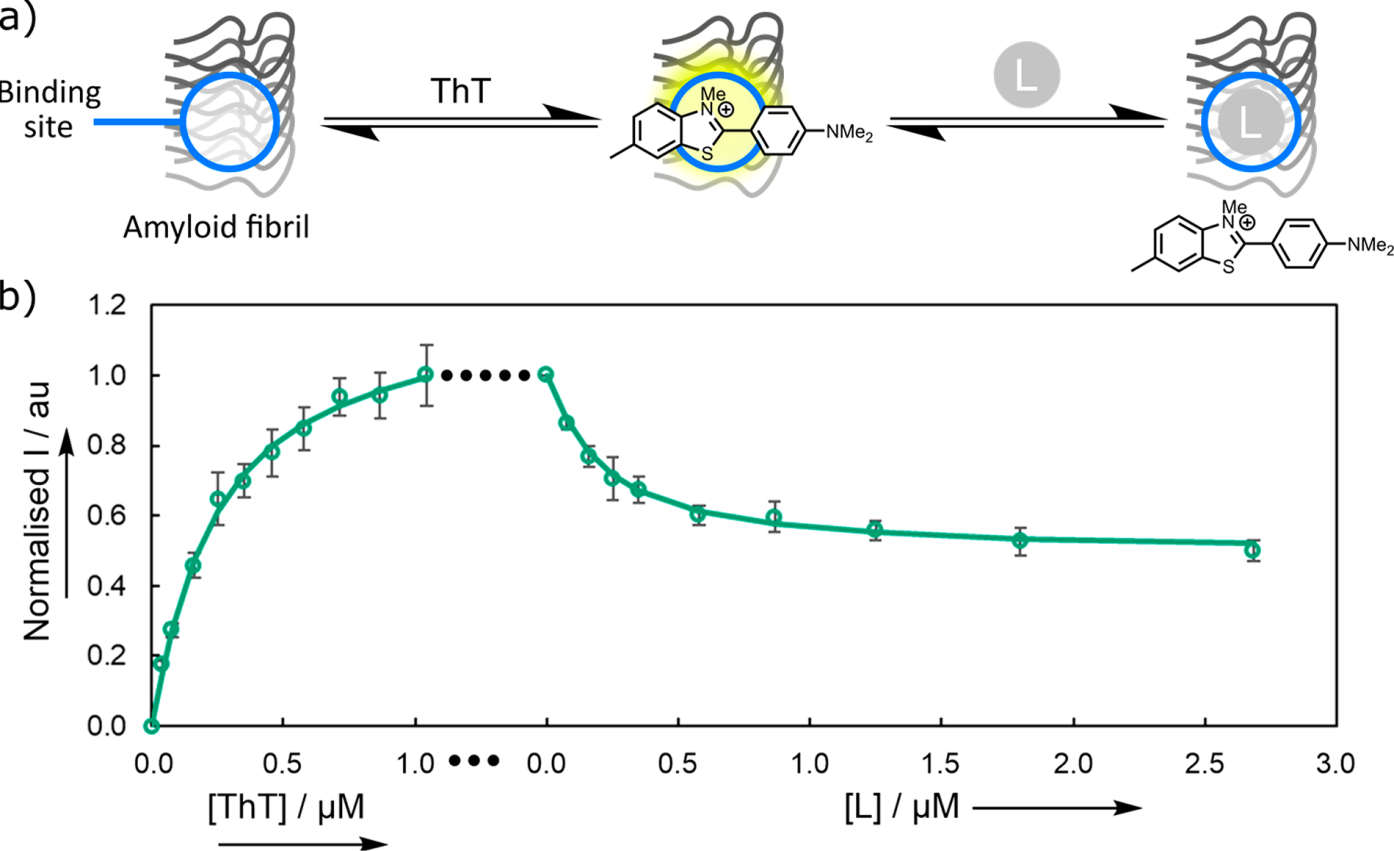
(a) Competition binding assay using thioflavin T (ThT). There is an increase in the fluorescence intensity when ThT binds to an amyloid fibril. Binding of a competing ligand L (**E570**) is detected by the decrease in fluorescence intensity when ThT is displaced. (b) Fluorescence titration of ThT into a solution of Aβ(1–42) fibrils (500 nM) in 1× PBS buffer (pH 7.4, 25 °C), followed by a titration of the competing ligand. Spectra were recorded using λ_ex_ = 440 nm, and the fluorescence emission was monitored at λ_em_ = 483 nm. The experimental measurements are shown as points (error bars represent the 95% confidence interval calculated from at least three independent experiments), and the lines are the best fits to eq 2 with log(*K*_d_(ThT)/M) = −6.7 and log(*K*_d_(L)/M) = −7.6.

Thioflavin T (ThT) competition assays were used to screen the 46 compounds selected from the VS pipeline (Figure 7a). When ThT binds to Aβ(1–42) fibrils, there is a large enhancement in the intensity of the fluorescence emission and a shift in the emission wavelength. Addition of a second nonfluorescent ligand L that binds to Aβ(1–42) fibrils at a ThT binding site will lead to a decrease in fluorescence intensity due to the displacement of the ThT. Titration of the second competing ligand (L) into a mixture of ThT and Aβ(1–42) fibrils therefore allows determination of the binding affinity. Figure 7b shows an example of this competition assay. When ThT binds to Aβ(1–42) fibrils in the first phase of the experiment, there is a characteristic increase in the fluorescence intensity. Addition of the competing ligand in the second phase of the experiment leads to a decrease in fluorescence intensity, due to displacement of the ThT from roughly half of the binding sites (Figure 7b).

Both the free and bound states of ThT fluoresce; therefore, the background fluorescence due to free ThT must be accounted for in analysis of the titration data. In addition, the presence of two different types of binding sites must be considered: S_1_, which binds both ThT and L, and S_2_, which is only accessible to ThT.

The intensity of the fluorescence emission (*I*) is therefore given by eq 2:
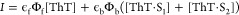
2where ϵ_f_ϕ_f_ and ϵ_b_ϕ_b_ are the products of the UV–vis absorption extinction coefficient and the fluorescence quantum yield for free and bound ThT, respectively, [ThT] is the concentration of free ThT, and [ThT·S_1_] and [ThT·S_2_] are the concentrations of ThT bound to S_1_ and S_2_, respectively.

The concentration of ThT bound to each site is given by eq 3:
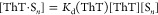
3where [S_*n*_] is the concentration of unbound site S_*n*_ (*n* = 1 or 2), and the dissociation constant of ThT, *K*_d_(ThT), is assumed to be the same for both sites.

The concentration of L bound to site S_1_ is given by eq 4:
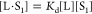
4where [L] is the concentration of free L, [L·S_1_] is the concentration of L bound to S_1_, and *K*_d_ is the dissociation constant.

The total concentrations of ThT, [ThT]_tot_ and L, [L]_tot_ are then given by eqs 5 and 6:
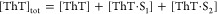
5
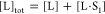
6The quantity ϵ_f_ϕ_f_ for ThT was measured from a dilution experiment in 1× PBS buffer (pH 7.4, 25 °C), and values of ϵ_b_ϕ_b_ and *K*_d_(ThT) were found by fitting eqs 2, 3, and 5 to a single-site binding model for the first phase of the experiment illustrated in Figure 7b, i.e., direct titration of ThT into Aβ(1–42) fibrils (−log(*K*_d_/M) = 6.7 ± 0.1; see SI for details).

**Figure 8:**
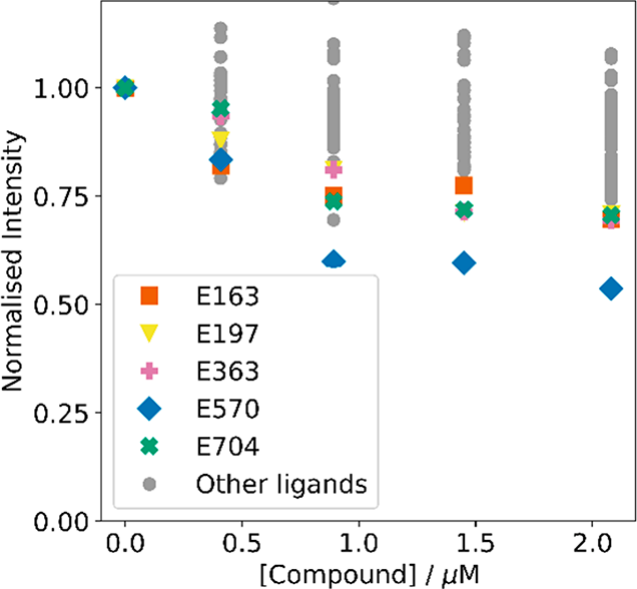
ThT competition assay for 44 compounds from the VS pipeline. Increasing concentrations of each compound were added to Aβ(1–42) fibrils (250 nM) and ThT (1.0 μM) in 1× PBS buffer (pH 7.4, 25 °C). Fluorescence spectra were recorded using λ_ex_ = 440 nm, and the emission intensity was monitored at λ_em_ = 483 nm. Gray data points denote low-affinity compounds that were not investigated further.

Figure 8 shows the result of titrating 44 of the 46 candidates from the VS into a mixture of Aβ(1–42) fibrils and ThT. The five compounds that displaced the greatest quantity of ThT (**E163**, **E197**, **E363**, **E570**, and **E704**) are highlighted as the colored data points in Figure 8, and these compounds were selected for further characterization. The other two candidates from the VS were fluorescent coumarin derivatives, so direct titration into Aβ(1–42) fibrils was used to assay these compounds instead of the competition assay. No binding was detected in these cases (see SI).

**Figure 9:**
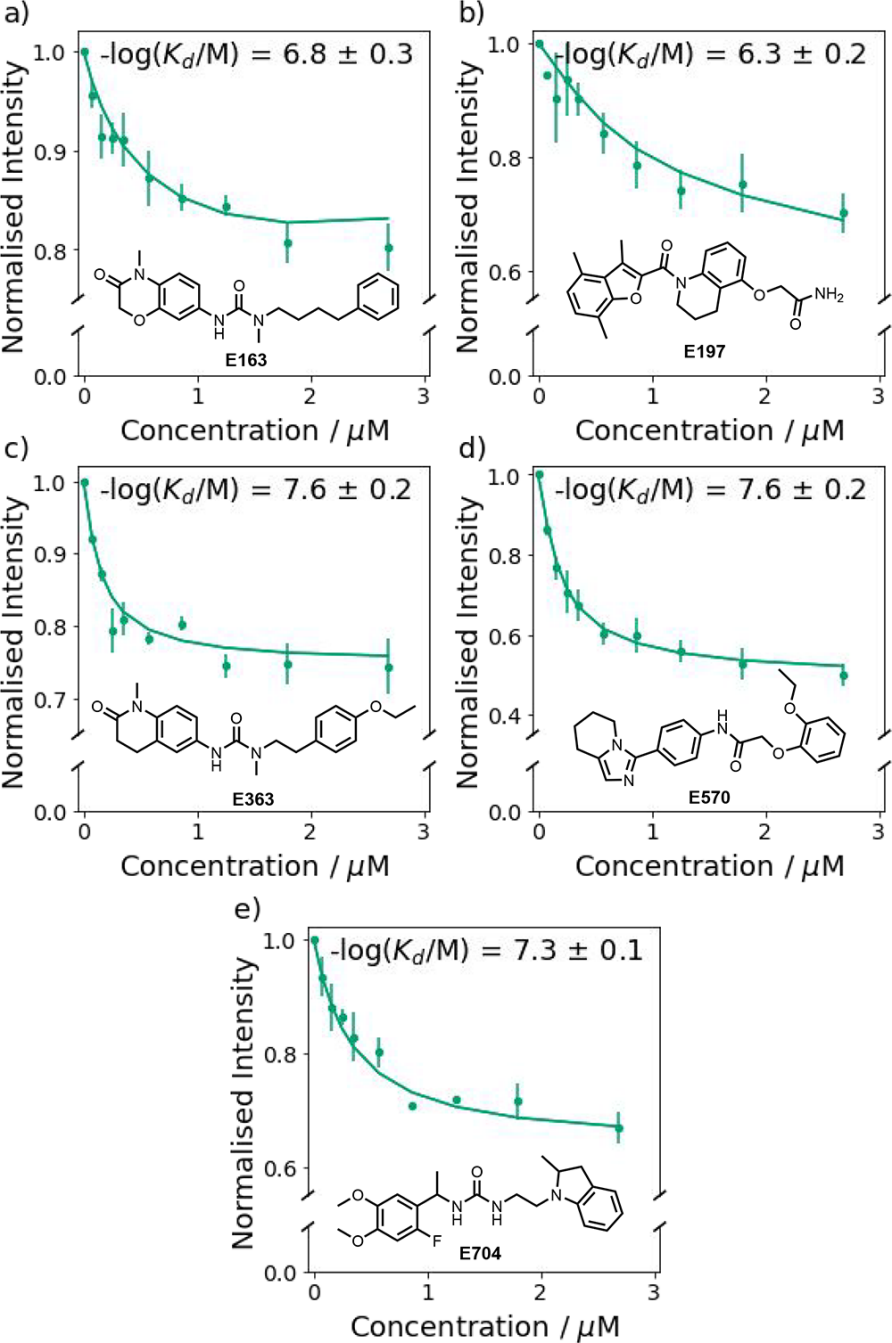
Fluorescence titration of (a) **E163**, (b) **E197**, (c) **E363**, (d) **E570**, and (e) **E704** into a mixture of Aβ(1–42) fibrils (500 nM) and ThT (1.0 μM) in aqueous 1× PBS buffer (pH 7.4, 25 °C). The spectra were recorded by using λ_ex_ = 440 nm, and emission was monitored at λ_em_ = 483 nm. The experimental measurements are shown as points (error bars represent the 95% confidence interval calculated from at least three independent experiments). The lines are the best fit to eq 2, and the resulting dissociation constants are shown.

Figure 9 shows the results of titration experiments used to measure the binding affinity of **E163**, **E197**, **E363**, **E570**, and **E704** for Aβ(1–42) fibrils. Fitting 1:1 binding isotherms yielded nanomolar dissociation constants for all five compounds (Table 3, 20–600 nM). The amount of ThT displaced varies from one compound to another, which indicates that the compounds target different subsets of ThT binding sites. **E570** displaces about half of the ThT, whereas the other four ligands displace only 20–40% of the bound ThT. One explanation for this result is that the ligands used for model development may bind at more than one site on the fibrils, and the compounds selected by the VS would therefore contain different combinations of features that favor binding at different sites. Partial ligand displacement is a potentially useful feature of these assays that may provide additional information on the nature and distribution of different binding sites that are present on different types of fibril.^34^

**Table 3:** Dissociation Constants for **E163**, **E197**, **E363**, **E570**, and **E704** Measured by Fluorescence Competition Assays into a Mixture of Aβ(1–42) Fibrils (500 nM) and ThT (1.0 μM) in Aqueous 1× PBS buffer (pH 7.4, 25 °C)^a^

compound	*K*_d_/nM	–log(*K*_d_/M)
**E163**	200 ± 100	6.8 ± 0.3
**E197**	600 ± 300	6.3 ± 0.2
**E363**	20 ± 10	7.6 ± 0.2
**E570**	20 ± 10	7.6 ± 0.2
**E704**	56 ± 6	7.3 ± 0.1

a The spectra were recorded using λ_ex_ = 440 nm, and emission was monitored at λ_em_ = 483 nm. Dissociation constants are given as the average of fits from at least three independent experiments.

**Figure 10:**
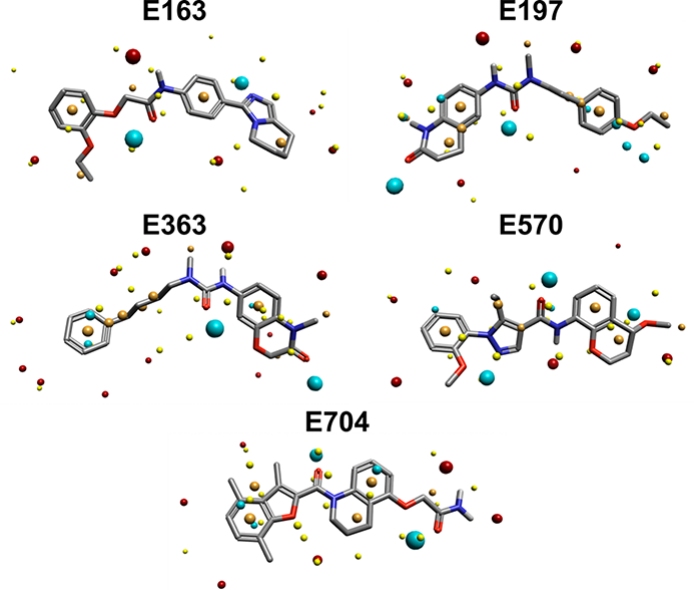
Field points of **E163**, **E197**, **E363**, **E570**, and **E704** aligned to Template 2. Blue field points describe regions of negative electrostatic potential; red field points describe regions of positive electrostatic potential; orange field points describe regions with high hydrophobicity; and yellow field points describe van der Waals interactions.

Figure 10 shows the field points for these ligands in the same alignment as Template 2. There is no obvious similarity between the field point distributions, which would be consistent with different binding site preferences and highlights the utility of the 3D models for finding structurally diverse ligands.

**Figure 11:**
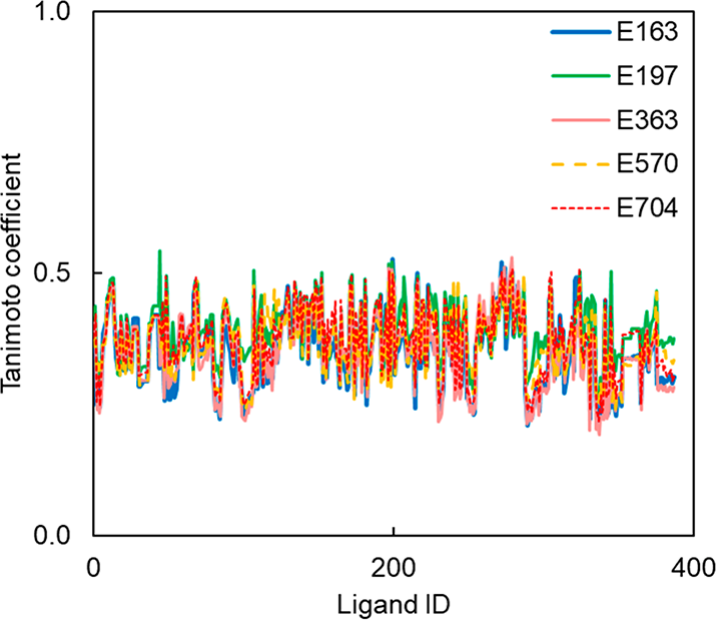
Tanimoto similarity coefficients between **E163**, **E197**, **E363**, **E570**, and **E704** and each ligand in the FBH database calculated using RDKit fingerprints.

**Figure 12:**
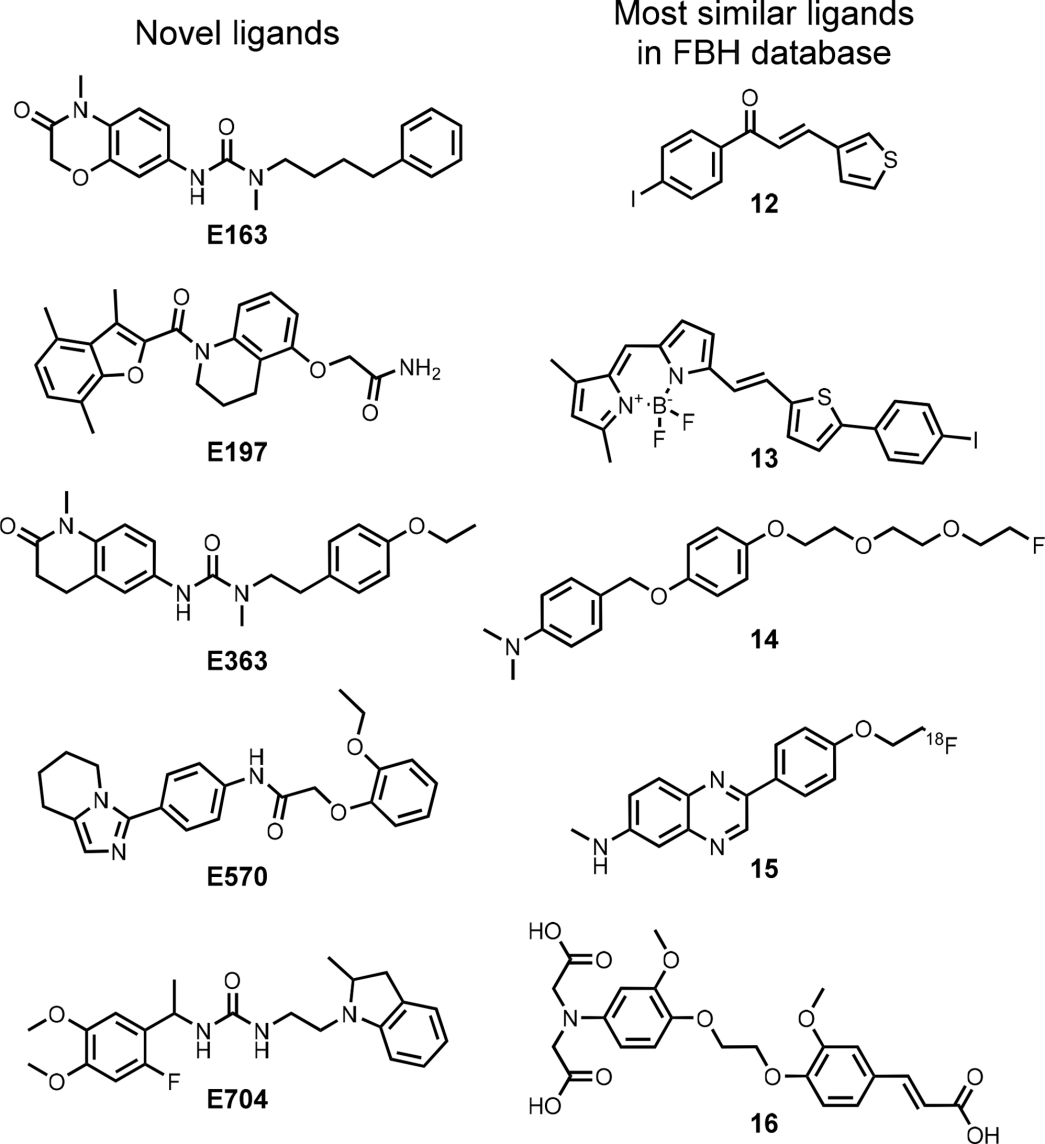
Comparison of the chemical structures of the novel ligands identified by the VS pipeline with the chemical structure of the corresponding ligand in the FBH database with the highest Tanimoto similarity coefficient: 0.53 for **E163** and **12**, 0.54 for **E197** and **13**, 0.53 for **E363** and **14**, 0.50 for **E570** and **15**, and 0.51 for **E704** and **16**.

RDKit fingerprints were used to calculate Tanimoto similarity coefficients between each of the five new ligands and each compound in the FBH database.^193,194^Figure 11 illustrates the results. The maximum values of the similarity coefficients are about 0.5 in all cases, indicating that the newly discovered ligands have chemical structures very different from those of all previously reported Aβ(1–42) ligands. Figure 12 compares the structures of **E163**, **E197**, **E363**, **E570**, and **E704** with the corresponding ligand in the FBH database, which has the highest Tanimoto coefficient.

## Conclusion

Amyloid fibrils present a challenging target for structure-based VS due to the lack of knowledge regarding binding site location and structure. Here, we describe a three-step ligand-based VS approach that exploits the wealth of Aβ(1–42) ligand data in the literature. A data set of 707 Aβ(1–42) fibril-binding ligands was first compiled, of which 388 had binding constants that reported on the same binding site, as determined by ligand competition assays. Key molecular properties required for binding were identified from the FBH database. The 698 million compounds in the ZINC15 database were filtered using charge, molecular weight, and log*P*, leading to 63 million compounds for further screening. The FBH database was used to train a support vector machine to predict dissociation constants by using computationally inexpensive chemical descriptors. This model was used to select the 10,000 compounds with the highest predicted affinities. The FBH database was used to train 3D models based on field points, which represent a description of surface, shape, and electronic properties.

These models were used to select 100 compounds with the highest predicted binding affinity, and 46 of these were experimentally investigated in fluorescence competition binding assays for Aβ(1–42) fibrils. The five highest affinity ligands all had nanomolar dissociation constants (25–500 nM) without any further structural optimization. The discovery of five new amyloid ligands from an experimental investigation of 46 compounds selected by the ligand-based VS pipeline represents a 10.9% hit rate. The VS pipeline also generated structurally diverse compounds that represent novel scaffolds for Aβ(1–42) ligands, which have not previously been reported.^195^ The conformational flexibility of the new ligands also suggests that the rigid, highly conjugated structures of the previously reported Aβ(1–42) ligands are not strictly required. The approach is not restricted to Aβ(1–42) ligands. For example, application of this methodology to *in vivo* data on ligand binding would be of particular interest to accelerate the discovery of novel high-affinity ligands for the biological fibrils associated with disease. New ligands for protein aggregates have a number of potential applications in disease diagnosis, including use as *in vivo* imaging agents or identification of different fibril polymorphs in tissue samples.

## Supporting Information Available

Details of the methods used to construct the virtual screening models, experimental details on preparation and characterization of protein aggregates, chemical structures of all ligands, and titration data (PDF)
