# Supplementary material for: Discovery of High-Affinity Amyloid Ligands Using a Ligand-Based Virtual Screening Pipeline
Source: J Am Chem Soc. 2023 Jul 13;145(29):15936–50. doi: 10.1021/jacs.3c03749 (PMC10375536; doi:10.1021/jacs.3c03749)
Supplement: Supplementary file 1 — ja3c03749_si_001.pdf [file ja3c03749_si_001.pdf]

Discovery of high-affinity amyloid ligands using a ligand-based virtual screening pipeline

Timothy S. Chisholm,[a] Mark Mackey [b] and Christopher A. Hunter [a,*]

[a] Yusuf Hamied Department of Chemistry, University of Cambridge, Lensfield Road, Cambridge CB2 1EW, UK. Email: herchelsmith.orgchem@ch.cam.ac.uk.

[b] Cresset, New Cambridge House, Bassingbourn Road, Litlington SG8 0SS, Cambridgeshire, UK

Supporting Information

Contents

Dataset Generation and Preparation 3
- Literature Search 3
- 3D Conformation Hunt 3
- Chemical Descriptors 4

Chemical Descriptor Model Development 4
- Regression Models 4
- Classification Models 4
- Model Scoring 4
- Nested Cross-Validation 5
- Fitting the Final Model 5

3D Model Development 6
- Reference Template Generation 6
- Compound Alignment and Partitioning 7
- Model Development 7
- Feature Importance 8
- Field QSAR Variance 9

Virtual Screening 10
- ZINC15 Database and Molecular Property Filter 10
- Predicting Binding Affinities 10
- Calculating Similarity 11

Materials and Instrumentation 13

Aβ(1-42) Aggregation 14
- Transmission Electron Microscopy 14
- Circular Dichroism 15

In Vitro Binding Assays 15
- General Procedure for Fluorescence Measurements 15
- Fluorescence Characterisation 16
- Saturation Binding Assays 17
- Competition Binding Assays 18
- Fluorescence Anisotropy Assays 18
- Ligand Dilution Series 18
- Data Fitting 22

References 22

FBH Database 24

Non-FBH Database 41

Experimental/Methods

Dataset Generation and Preparation

Literature Search

Protein-ligand binding data were acquired from literature searches using Web of Science and Google Scholar, and the PDBbind and ChEMBL online databases.[1,2] The requirements to be included in this database were 1) a chemical structure, and 2) an experimentally determined dissociation constant for binding to Aβ(1-42) fibrils. A total of 707 unique ligands were found. Of these ligands, 44 had K_d values reported as limiting values (eg. K_d > 1 µM). Binding measurements given as IC_{50} values were converted to K_d values where possible.

The dataset was refined by considering only datapoints corresponding to a common binding site. Ligands **1**-**4** have a common 6,5-fused benzoheterocycle (FBH) structural core and therefore likely target the same subset of binding sites. This subset of binding sites is referred to as the *FBH binding site*. Ligands **5**-**8** in Figure S1 also appear to target the same binding site. The dissociation constants of ligands **5**-**8** were similar when measured by (a) a direct binding assay, and (b) a competition binding assay against one of **1**-**4**. Of the Aβ(1-42) data, 388 datapoints were found to correspond to the FBH binding site. Of this subset of the data, 361 datapoints had given values reported for binding constants, and 27 datapoints had limiting values reported for binding constants.

For ligands where multiple binding constants were reported, the average of these measurements were used. The log of all binding constants in M^{-1} were taken and the $\log(K_d/M)$ values recorded. A binding class was assigned to each ligand based on this $\log(K_d/M)$ value; class 0 for $\log(K_d/M) \leq -8$, class 1 for $-8 < \log(K_d/M) \leq -7$, class 2 for $-7 < \log(K_d/M) \leq -6$, and class 3 for $-6 < \log(K_d/M)$.

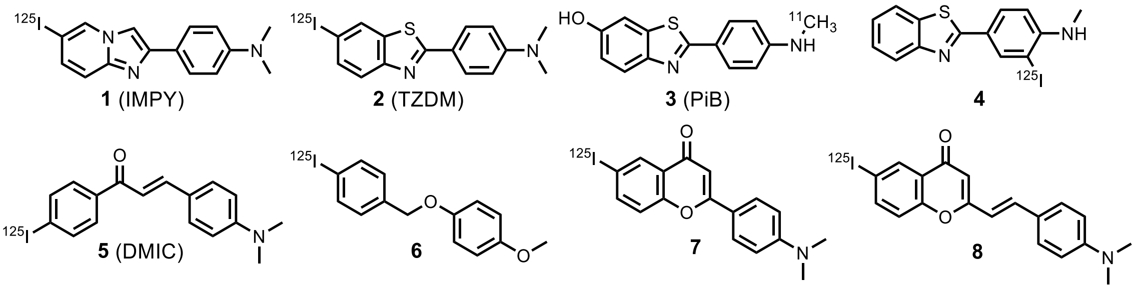

Figure S1. Aβ(1-42) ligands which target the FBH binding site**.**

3D Conformation Hunt

Conformational hunts for all ligands in the FBH dataset were performed using the "Accurate but Slow" calculation method in Forge. For each ligand, 200 conformations were generated with a gradient cut-off of 0.100 kcal/mol/A for conformer minimisation. Duplicate conformers were filtered at an RMS of 0.50 A, and an energy window of 3.00 kcal/mol were used. No coulombic or attractive vdW forces were considered. Field points were then generated by Forge using the XED (eXtended Electron Distribution) molecular mechanics forcefield. Positive electrostatic, negative electrostatic, van der Waals, and hydrophobic field points were calculated. The XED force field uses several monopoles to generate a multipole description of the underlying force fields.

Chemical Descriptors

Molecular descriptors were calculated using the Python cheminformatics toolkit RDKit.[3] A set of 45 molecular descriptors were calculated for each ligand using the *Descriptors*, *rdMolDescriptors*, *Lipinksi*, and *Descriptors3D* modules of RDKit. Only descriptors calculated using the *Descriptors3D* module are dependent on molecular conformation. These descriptors describe computed experimental properties (*eg.* log*P*, molar refractivity), 1D compositional properties (*eg.* heavy atom counts, molecular weight, number of hydrogen bond donors), 2D topological properties (*eg.* topological polar surface area), and 3D conformational properties (*eg.* asphericity, eccentricity). The Pearson correlation coefficient was less than 0.9 for all pair-wise comparisons of the descriptors, indicating that they can be treated as independent variables.

Each set of descriptors X was standardised using Equation S1,

$$X' = \frac{X - \mu}{\sigma}, \qquad \text{Eq.S1}$$

for the mean μ and standard deviation σ across the dataset. This transformation centres the value of each descriptor around the mean with a unit standard deviation.

This collection of ligand-based features are referred to as “chemical descriptors” within this work.

Chemical Descriptor Model Development

Regression Models

Two baseline regression models were created. The first baseline model predicted the log(K_d/M) of each ligand as the average log(K_d/M) of the entire FBH dataset. The second baseline model randomly reallocated every log(K_d/M) value to each ligand. The regression models studied were a random forest regressor, extra-trees regressor, X-gradient boosted regressor,[4] light-gradient boosted machine regressor,[5] gradient boosted regressor, ada-boost regressor,[6] histogram-based gradient boosted regressor, and a support vector machine regressor with a rbf kernel. These models were evaluated with the described nested cross validation procedure and scored using mean absolute error between the predicted and experimental log(K_d/M) values.

Classification Models

A baseline model that randomly reallocated the binding class between ligands was used. The classification models studied were a random forest classifier, X-gradient boosted classifier,[4] light gradient-boosted machine classifier,[5] a gradient boosted classifier, a histogram-based gradient boosted classifier, an ada-boosted classifier,[6] an extra-trees classifier, and a support vector machine classifier with a rbf kernel. These models were evaluated with the described nested cross validation procedure and scored using balanced accuracy.

Model Scoring

Regression models were scored using the mean absolute error (MAE) between the experimental and predicted log(K_d/M) values. MAE is calculated using Equation S2:

$$MAE = \frac{\sum_{i=1}^{n} |\log(K_{pred,i}/M) - \log(K_{exp,i}/M)|}{n}, \qquad \text{Eq.S2}$$

Where log($K_{pred,i}$/M) is the predicted log(K_d/M) of the ith ligand, log($K_{exp,i}$/M) is the experimentally measured log(K_d/M) of the ith ligand, and n is the number of ligands.

All classification models were scored using the balanced accuracy, which is defined as the average of recall for each class. For a given class, the recall is defined in Equation S3 as

$$Recall = \frac{TP}{TP + FN}, \qquad \text{Eq.S3}$$

where *TP* is the number of true positives, and *FN* is the number of false negatives. For a multiclassification problem, the balanced accuracy is defined in Equation S4 as

$$Balanced\ accuracy = \frac{\sum_{i=1}^{n} Recall_i}{n}, \qquad \text{Eq.S4}$$

where $Recall_i$ is the recall for the ith class out of a total *n* classes.

Nested Cross-Validation

Nested cross-validation (CV) was used for hyperparameter optimisation and model evaluation. Both the outer and inner CV procedures used random *k*-fold cross validation (*k* = 5).

The outer CV loop uses a random *k*-fold cross validation (*k* = 5) procedure. Within each loop the inner CV loop is called using the outer CV training set and model hyperparameters are optimised. The outer test set is then used to evaluate model accuracy. Final model accuracy is the average model accuracy over the *k* folds.

The inner CV loop uses a *k*-fold cross validation (*k* = 5) procedure to evaluate model accuracy while optimising hyperparameters using Optuna.[7]

Optuna suggests model hyperparameter values from a user-defined window which are then used to fit the inner CV training set. The inner CV test set is then used to evaluate model accuracy. Final model accuracy is the average model accuracy over the *k* folds. This procedure was repeated 200 times using new hyperparameter values suggested by Optuna with each iteration. The hyperparameters which produced the model with the highest accuracy were then passed to the outer CV loop for model evaluation.

Fitting the Final Model

To develop final models, a support vector machine regressor (rbf kernel) and random forest regressor were fitted by passing the entire dataset into the inner cross-validation procedure.

3D Model Development

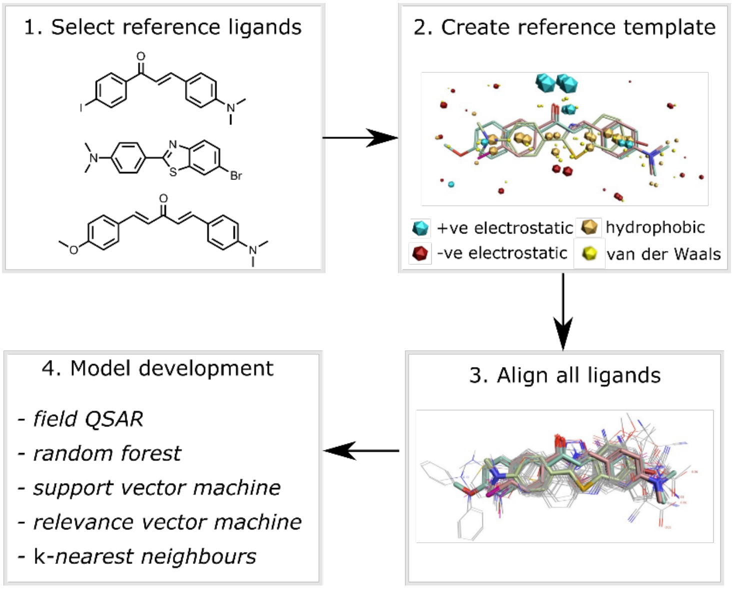

Figure S2. Model development using Forge. 1) High-affinity reference ligands were selected with representative structures. 2) A diverse set of conformations were generated for each reference ligand. Conformations of each ligand were aligned to one another based on similarity in their calculated field points and shape. Blue field points describe regions of negative electrostatic potential, red field points describe regions of positive electrostatic potential, orange field points describe regions with high hydrophobicity, and yellow field points describe van der Waals interactions. 3) Conformations of all other ligands in the dataset were generated and aligned to the reference template. 4) Quantitative structure activity relationship (QSAR) models were developed from the relationship between the field points and the experimentally measured log(K_d/M) for each ligand.

Reference Template Generation

The FieldTemplater module of Forge (Cresset Inc., UK) was used to generate reference templates for ligand alignment. The ligands **5**, **9**, **10**, and **11** were chosen as reference molecules due to (a) their low nanomolar binding affinities, and (b) their structural core being representative of a large part of the known dataset (Figure S3). A conformational hunt of these ligands was performed as above, combined with an alignment step that was scored by a 50:50 consideration of shape similarity and fieldpoint alignment between ligands. Templates were generated with a minimum of three molecules, a maximum number of 100 comparisons between pairs, a maximum score delta of 0.10 per pair, a minimum link density of 0.80, and duplicate templates were filtered at an RMS of 0.7 A. Two templates were generated using this procedure: Template 1 was comprised of ligands **5**, **9**, and **10** in their lowest energy conformation with a field similarity score of 0.710, a shape similarity score of 0.806, and an overall similarity score of 0.758. Template 2 was comprised of ligands **5**, **9**, **10**, and **11**, with all ligands in their lowest energy conformation aside from ligand **11** which occupied it's 17th-lowest energy conformation. This template had a field similarity score of 0.626, a shape similarity score of 0.749, and an overall similarity score of 0.687.

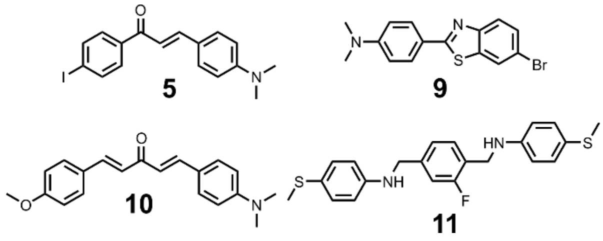

Figure S3. Structures of the reference ligands used for Forge model development.

Compound Alignment and Partitioning

Compound alignment to each reference template was performed using a "Normal" calculation method in Forge. A maximum scoring method was used to align ligands to the templates containing multiple reference compounds. Alignment was scored by a 50/50 weighting of shape similarity and field point alignment. Once alignments were generated they were manually verified. Ligands that failed to align were discarded from the dataset. The remaining ligands were then partitioned into training and test set compounds in approximately a 4:1 ratio (Template 1: 169 training and 43 test set compounds; Template 2: 176 training and 46 test set compounds). This partitioning was activity-stratified and performed manually to ensure each structural class was represented in both datasets.

Model Development

Five models were created for each template.

1. **Field QSAR Model**

Field quantitative structure-activity relationship (QSAR) models were created using a "Normal" calculation method in Forge. A maximum of 20 model components were allowed, with a sample point minimum distance of 1.0 A and 50 Y scrambles. Leave-one-out CV was used for model evaluation.

2. **k-Nearest Neighbour (kNN)**

kNN models were created with a maximum of 20 neighbours, and using molecular fields as the measure of similarity with 50% of this similarity coming from the shape of the ligand.

3. **Random Forest (RF)**

RF models were built using 1000 trees, a feature subsampling fraction of 0.33, and a minimum of 5 samples per leaf. A sample point minimum distance of 1.0 A was used. Both electrostatic and volume fields were used for model development. The generalisation error is calculated as a cross-validated coefficient of determination (q^2) based on *out-of-bag* scores. These scores are calculated for a particular molecule by computing the average prediction from trees not trained using that molecule.

4. **Support Vector Machine (SVM)**

SVM models were built with a sample point minimum distance of 1.0 A. For cross-validation, 5 folds were used in *k*-fold CV to calculate a cross-validated coefficient of determination (q^2). A maximum of 50 optimiser iterations were allowed with a time limit of 60 min for global optimisation. A gamma between 10^{-5} and 10^{-1} was allowed, and a C between 10^{-1} and 10^3, and an epsilon between 10^{-4} and 1. Both electrostatic and volume fields were used for model development.

5. Relevance Vector Machine (RVM)

RVM models were built with a sample point minimum distance of 1.0 A. For cross-validation, 5 folds were used in *k*-fold CV to calculate a cross-validated coefficient of determination (q^2). A maximum of 50 optimiser iterations were allowed with a time limit of 60 min for global optimisation, and a gamma between 10^{-5} and 10^{-1} allowed. Both electrostatic and volume fields were used for model development.

Feature Importance

The feature importance of each descriptor included in the final random forest model was computed (Figure S4).

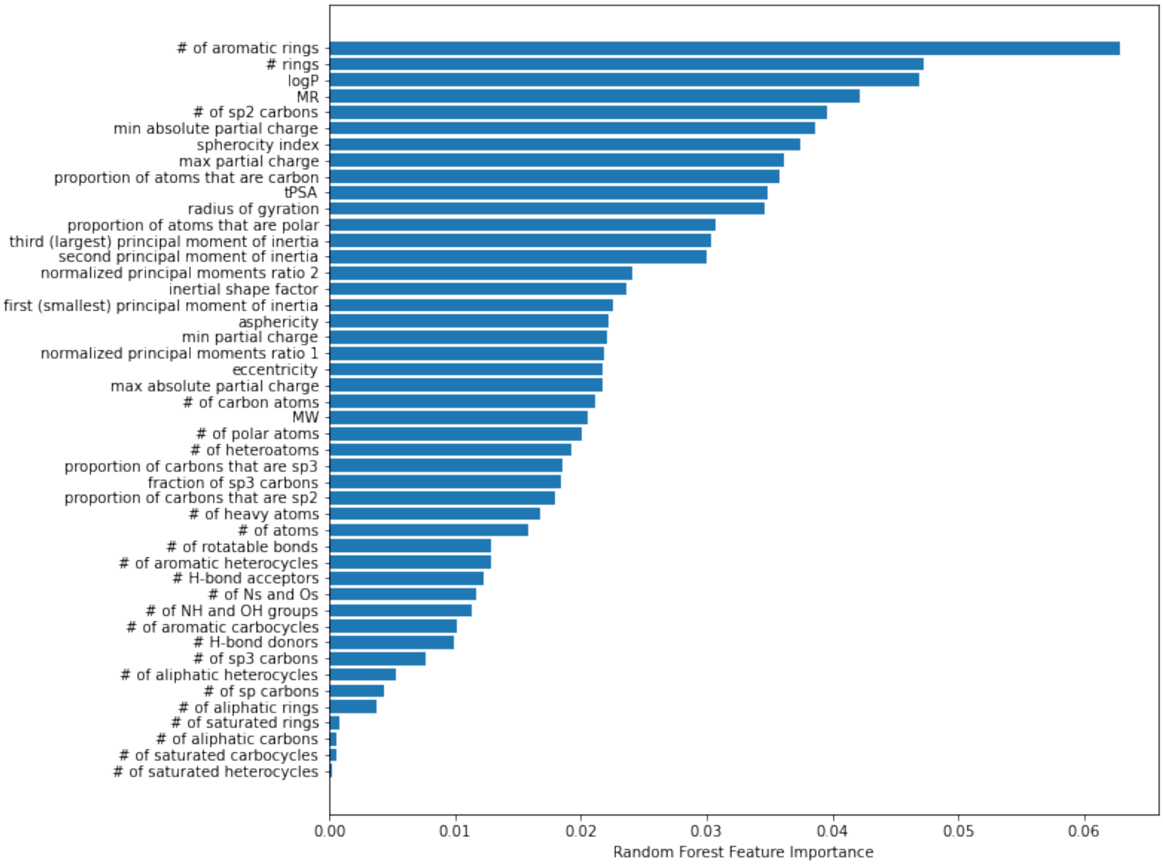

Figure S4. The feature importance of each descriptor included in the random forest regressor model.

Field QSAR Variance

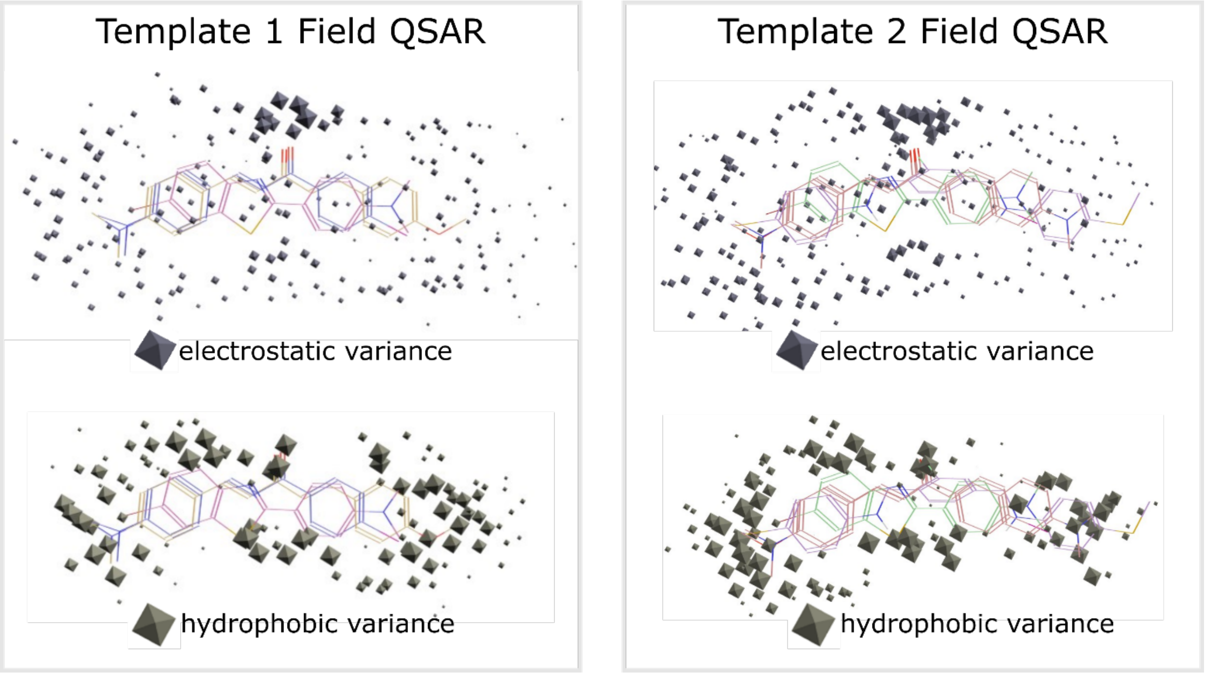

Figure S5. Variance in the model coefficients of Field QSAR models for each template.

Virtual Screening

ZINC15 Database and Molecular Property Filter

Tranches of compounds with 3D conformations were downloaded from the ZINC15 database corresponding to molecules that (a) were neutrally charged, (b) had a molecular weight between 200 and 500 Da, and (c) had c*log*P values between 3.5 and 5.5.[8] These tranches corresponded to 63 million ligands. Chemical descriptors were calculated for these compounds as described above.

Predicting Binding Affinities

The *log*(K_{pred}/M) of the 63 million ligands from the ZINC15 database were predicted using the final support vector machine regressor (rbf kernel) model. The top 10,000 ligands were then screened using Forge 3D models.

Four 3D models developed using Forge were used: the Field QSAR and RF models from Template 1, and the Field QSAR and SVM models from Template 2. Each of these models were used to predict the binding affinity of the 10,000 ligands passed from the chemical descriptor model. These 3D models predicted a broader range of -log(K_d/M) values compared to the SVM Chemical Descriptor model (Figure S6). The Field QSAR models predict a wider distribution of -log(K_d/M) compared to the RF and SVM models.

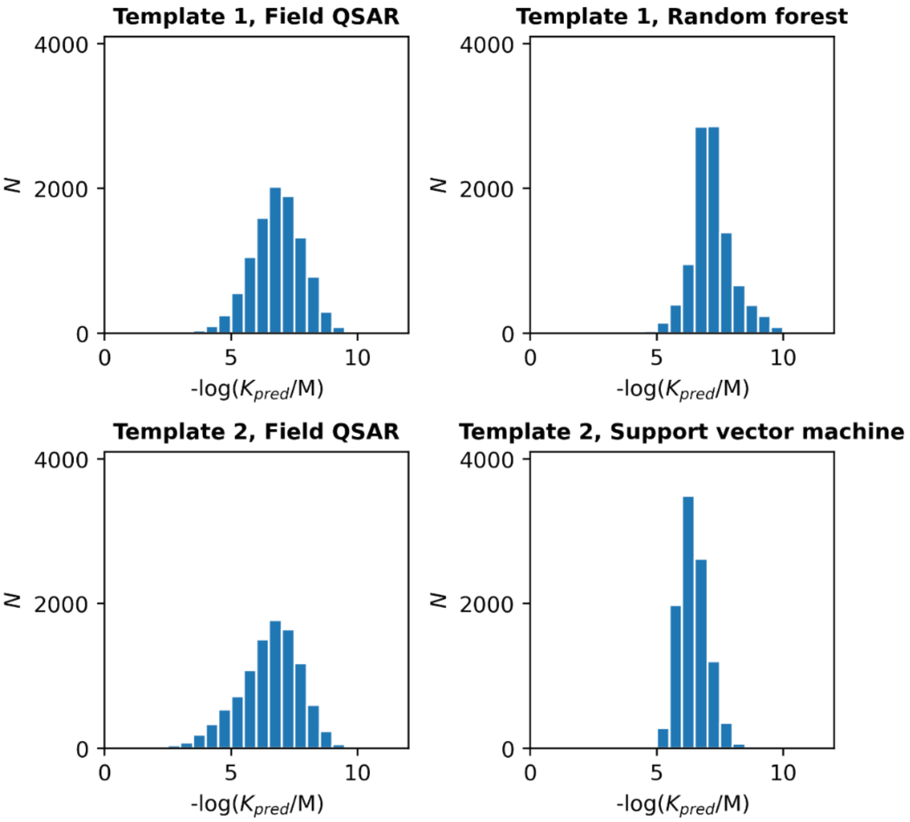

Figure S6. The predicted log(K_d/M) values for the 10,000 top-ranked Chemical Descriptor ligands screened through each 3D model.

The Field QSAR models calculated a "Distance to the model" score. This score is a measure of how well the field points of the screened ligand are represented in the trained model. If the screened ligand has many field points that were not found in the data used to create the model, then the model will be

unreliable for predicting ligand activity. Only ligands with a “Good” or “Excellent” distance to model score were therefore considered further.

The predicted affinity for the screened ligands were averaged between the two molecules for each template (ie. The predicted ligand affinity from the Template 1 Field QSAR and Template 1 RF models were averaged, and the predicted ligand affinity from the Template 2 Field QSAR and Template 2 SVM models were averaged). The two resultant rankings were then score-fused, and the top 25 predicted ligands from each template were purchased from Enamine (Ukraine) for screening. Of these ligands, 46 were successfully synthesised by Enamine (Ukraine) (Figure S7).

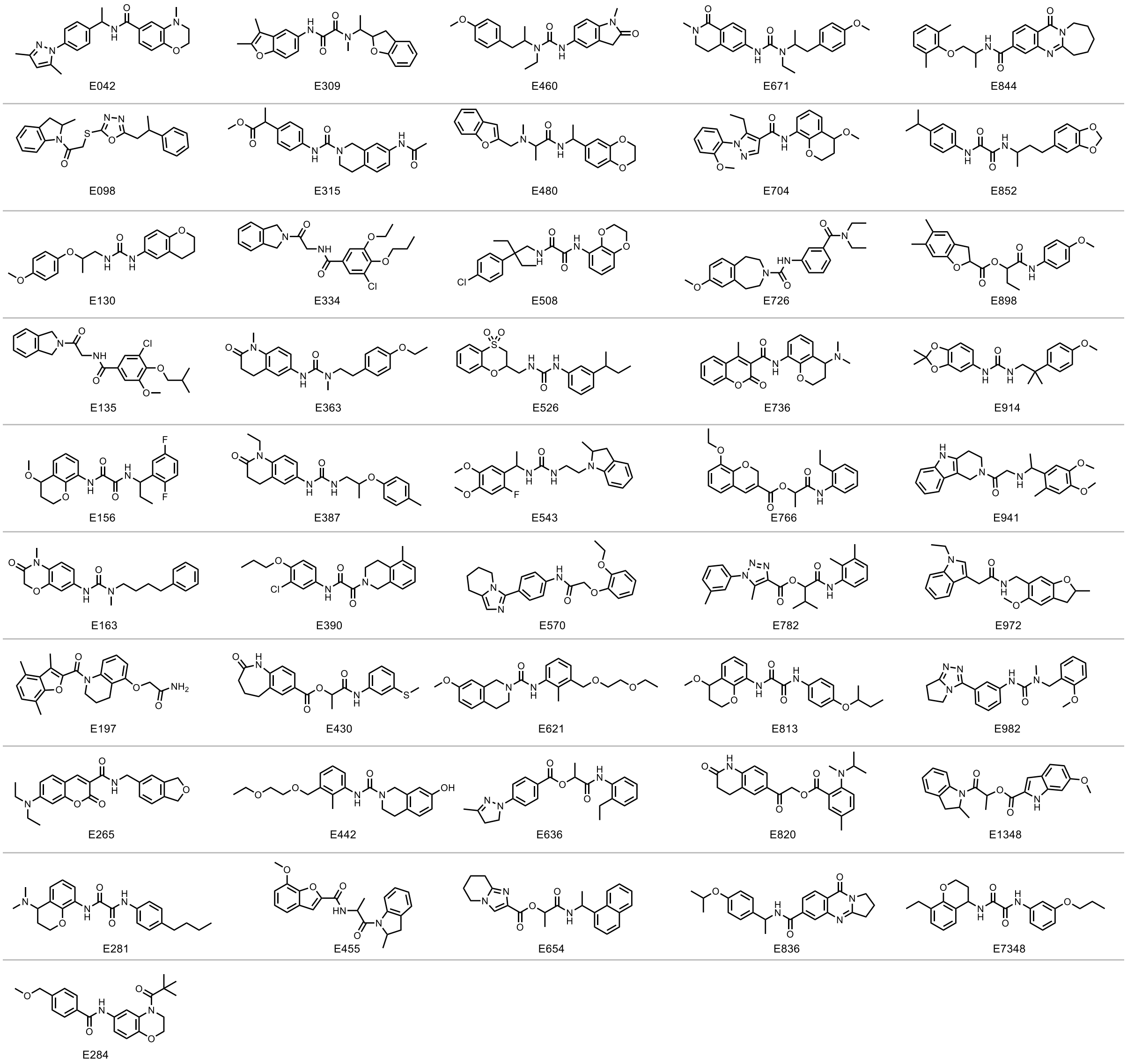

Figure S7. The 46 compounds purchased for experimental screening.

Calculating Similarity

RDKit fingerprints were calculated for **E163**, **E197**, **E363**, **E570**, and **E704**, and the FBH ligand database. Tanimoto and Dice similarity coefficients were then calculated between each of **E163**, **E197**, **E363**, **E570**, and **E704**, and the FBH ligand database.[9–12] Plots of the calculated similarity coefficients are shown in Figure S8. The most similar pairs are shown in Figure S9, with calculated similarity coefficients in Table S1.

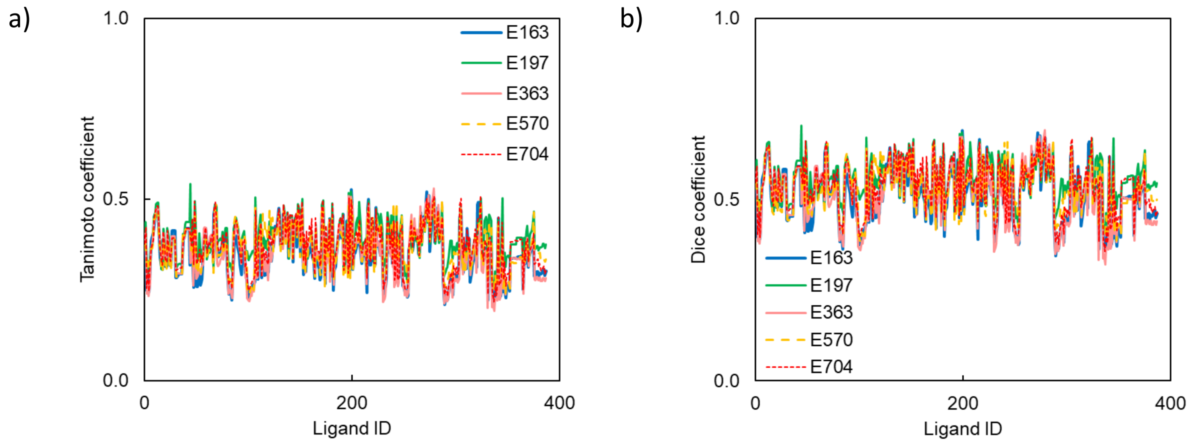

Figure S8. The calculated (a) Tanimoto similarity coefficients and (b) Dice similarity coefficients between **E163**, **E197**, **E363**, **E570**, and **E704**, and each ligand in the FBH database. RDKit fingerprints were used for similarity calculations.

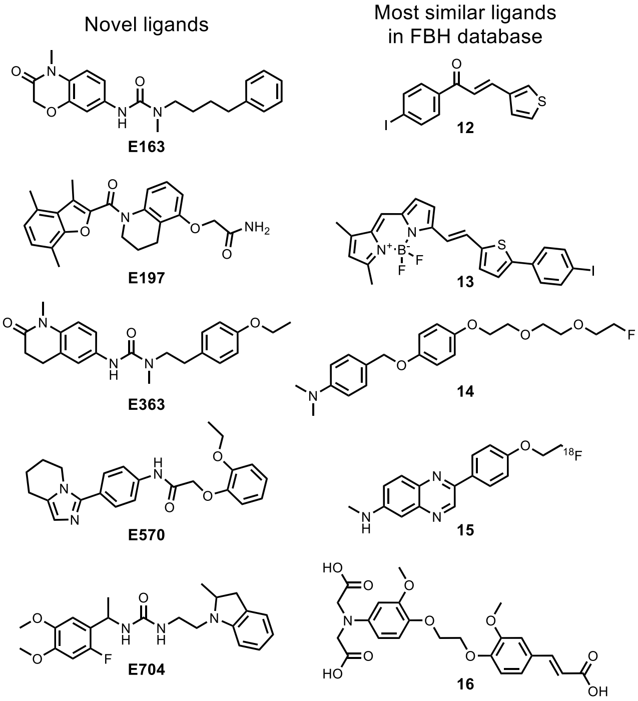

Figure S9. The chemical structures of the ligands in the FBH database with the highest Tanimoto and Dice similarity coefficients to each of the novel ligands discovered. The ligands with the highest Tanimoto coefficients also had the highest Dice coefficients.

Table S1. The calculated Tanimoto and Dice coefficients for the pairs shown in Figure S9.

	**E163** and **12**	**E197** and **13**	**E363** and **14**	**E570** and **15**	**E704** and **16**
Tanimoto similarity coefficient	0.53	0.54	0.53	0.50	0.51
Dice similarity coefficient	0.69	0.70	0.69	0.67	0.68

Materials and Instrumentation

All solvents and chemicals were obtained from commercial sources and used without further purification unless otherwise stated. Protein LoBind (Eppendorf) microtubes were used for preparing and storing all solutions containing Aβ(1-42). Low retention pipette tips were used for all fluid handling. Ligands used for experimental screening were purchased from Enamine (Ukraine).

2-(4-(dimethylamino)phenyl)-3,6-dimethylbenzo[d]thiazol-3-ium chloride (Thioflavin T, ThT)

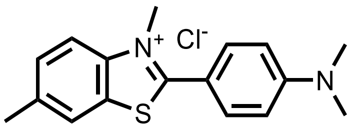

Thioflavin T (1.13 g, 3.54 mmol) was purchased from Sigma Aldrich as the chloride salt with dye content ≥65%. Thioflavin T was recrystallised twice from hot water prior to use (0.531 g, 1.67 mmol, 47%).

***^{1}H NMR (400 MHz, $CDCl_3$), δ(ppm)**:* 8.05 – 7.97 (m, 2H), 7.86 (d, *J* = 8.5 Hz, 2H), 7.53 (d, *J* = 8.5 Hz, 1H), 6.82 (d, *J* = 8.4 Hz, 2H), 4.55 (s, 3H), 3.12 (s, 6H), 2.49 (s, 3H).

^{13}C NMR (101 MHz, $CDCl_3$) δ(ppm): 172.57, 163.88, 154.15, 141.00, 139.09, 132.78, 131.27, 128.25, 123.44, 116.73, 112.40, 111.00, 77.48, 77.16, 76.84, 40.28, 39.65, 21.64.

HRMS (ESI+): 283.1265 m/z: Calculated for $C_{17}H_{19}N_2S^+$ = 283.1269 $[M]^+$.

IR (ATR, cm^{-1}): 3403, 1604, 1501, 1480, 1441, 1387, 1350, 1233, 1212, 1158, 827.

MP: 195.5-196.5 °C

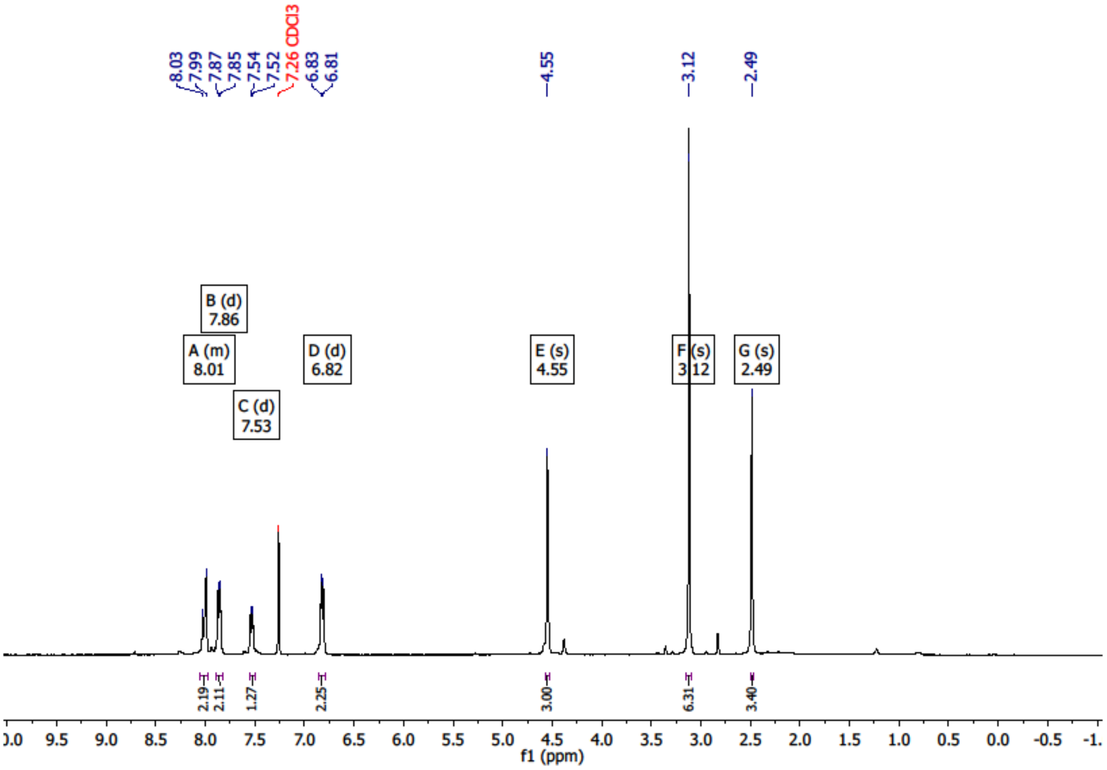

Figure S10: ^{1}H NMR spectra of ThT.

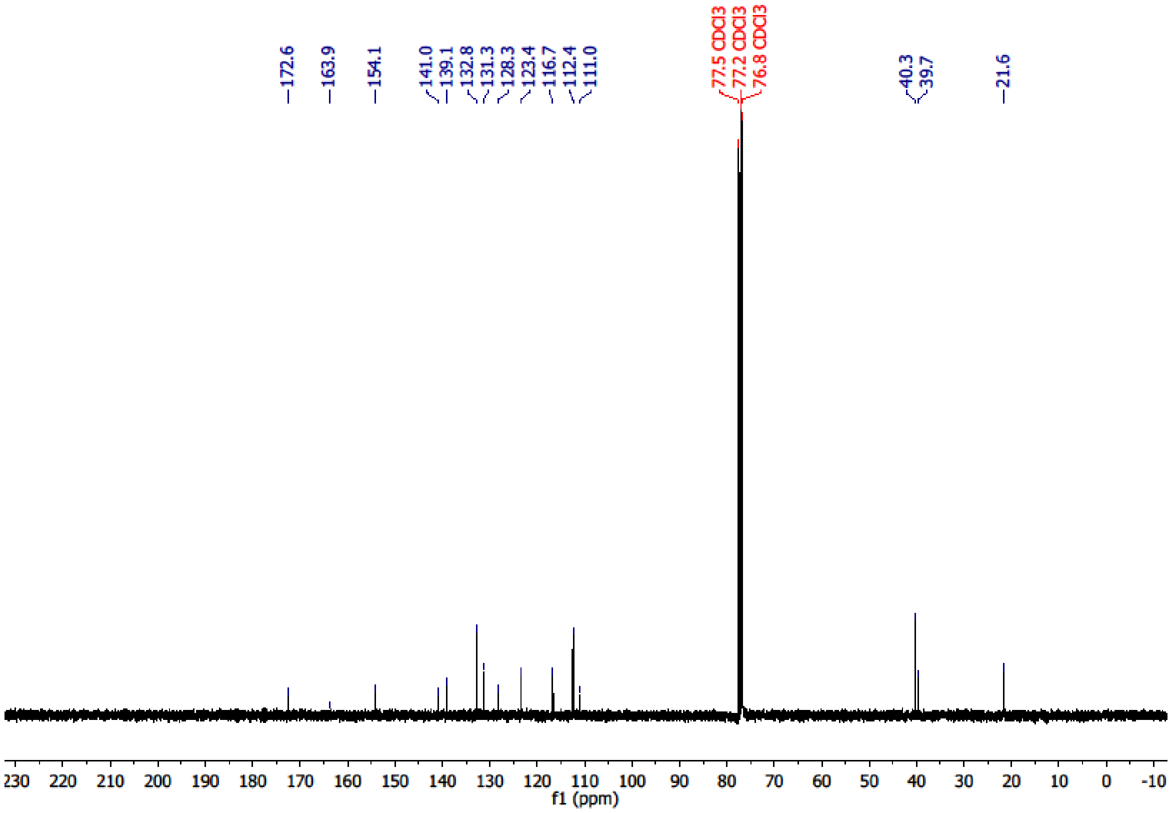

Figure S11: ^{13}C NMR spectra of ThT.

Aβ(1-42) Aggregation

Preparation and aggregation of Aβ(1-42) was performed based on standard literature procedures.[13,14] Monomeric Aβ(1-42) (1 mg: Rockland Immunochemicals, Limerick, PA, Lot #31541) was dissolved in 2 mM NaOH (1 mg/mL) on ice and gently agitated until the lyophilisate was wetted. The monomer was then left for 3 min to dissolve and a homogeneous appearance of the solution was achieved. The solution was then sonicated for 1 min, added to an Amicon Ultra-15 Centrifugal filter (30 kDa MWCO), and centrifuged (30 min, 4000 x g, 4 °C). The filter was washed with 2 mM NaOH (1 mL) and centrifuged (30 min, 4000 x g, 4 °C). The combined filtrates were kept on ice and gently rotated to homogenise the mixture. The absorbance at 280 nm was then measured (ε = 1440 M^{-1} cm^{-1}) to determine the concentration of the solution (165 µM). The solution was then added to 2xPBS (2 mL, pH 7.1) to afford a final stock solution in 1xPBS (pH 7.4). This solution was then incubated at 37 °C for 72 h with agitation using a magnetic stir bar. Aliquots were than taken and stored at -78 °C until use.

Transmission Electron Microscopy

Nanoscale morphologies of fibril samples were observed by TEM using a Thermo Scientific (FEI Company) Talos F200X G2 microscope operating at 200 kV (Figure S12). Images were recorded with a Ceta 4k x 4k CMOS camera. For sample preparation, TEM grids (continuous carbon film on 300 mesh Cu) were glow discharged using a Quorom Technologies GloQube at 25 mA for 60 s. A 2 µL sample of fibril in 1xPBS (1 µM Aβ(1-42)) was placed on a freshly glow-discharged grid, and after 1 min was carefully removed by blotting with filter paper. The sample was negatively stained using 2.0 µL of 2% (w/v) uranyl acetate solution in ethanol for 30 s. The grid was blotted and dried in air for 10 min at room temperature before use.

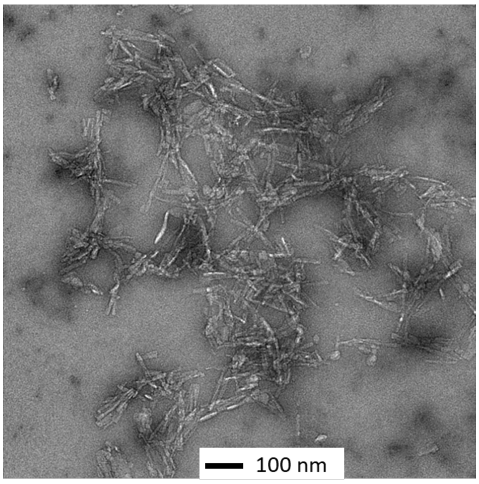

Figure S12. Representative TEM micrograph of the Aβ(1-42) fibrils used.

Circular Dichroism

CD spectra of Aβ(1-42) in 1xPBS (pH 7.4) were recorded with a Chirascan CD1 Spectrometer (Applied Photonics Ltd.) equipped with a Series 800 Temperature Controller (Alpha Omega Instruments) (Figure S13). Far-ultraviolet measurements (190 nm - 250 nm) of Aβ(1-42) (1.0 µM) in 1xPBS (pH 7.4) were recorded at 25 °C with a 10 mm optical pathlength, a time-per-point of 1.0 s, and a wavelength step of 0.1 nm. CD spectra were averaged over six scans. Data were baseline corrected by subtracting the complete buffer spectrum of 1xPBS (pH 7.4) averaged over six scans. Applied Photophysics Pro-Data Chirascan software was then used to smooth the data using Savitsky-Golay smoothing and a window size of eight, then used to convert the data to molar ellipticity.

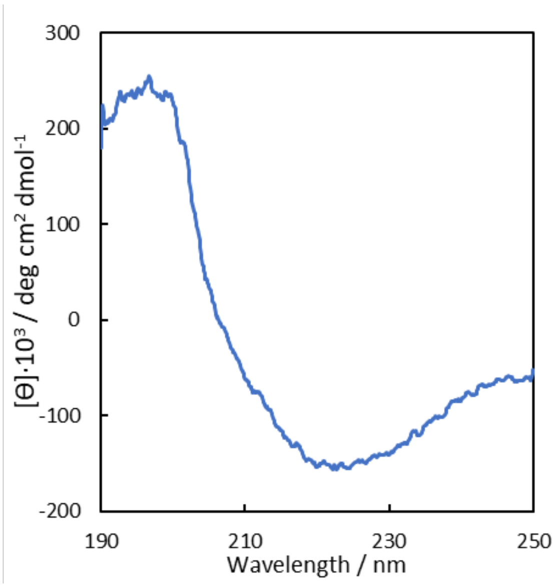

Figure S13. CD spectra of Aβ(1-42) (1.0 µM) in 1xPBS (pH 7.4, 298 K).

In Vitro Binding Assays

General Procedure for Fluorescence Measurements

Fluorescence spectroscopic data were measured using a Cary Eclipse Fluorescence Spectrophotometer G9800A (Agilent) controlled by Cary Eclipse software. Fluorescence excitation experiments were performed using 20 nm excitation and emission slits, a scan rate of 600 nm/min, a

data interval of 1.0 nm, an averaging time of 0.10, and medium PMT voltage at 25°C. Experiments detecting ThT used λ_{ex} = 440 nm, and measured emissions from λ_{em}= 470 – 600 nm. Stock solutions of all ligands were prepared in DMSO (1 mM). Stock solutions of Aβ(1-42) fibrils (10 µM) were prepared in 1xPBS (pH 7.4). Titration solutions were prepared by dilution of the corresponding stock solutions using 1xPBS (pH 7.4).

Fluorescence anisotropy experiments were performed using 10 nm excitation and emission slits, a scan rate of 120 nm/min, a data interval of 1.0 nm, an averaging time of 0.5 s, and medium PMT voltage at 25°C.

Fluorescence Characterisation

A stock solution of ligand was prepared in pure DMSO (1 mM). Dilutions of the ligand (10 µM) were then prepared in 1xPBS (pH 7.4), optionally containing Aβ(1-42) fibril (10 µM) as prepared above. Fluorescence spectra were then taken at 298 K.

Two fluorescent coumarins, **E265** and **E736**, were identified from VS. The coumarin **E265** exhibited similar excitation and emission wavelengths to ThT, while the fluorescence spectra of **E736** was more blue shifted (Table S1). Neither coumarin experienced a significant enhancement in fluorescence upon addition to Aβ(1-42) fibrils, nor a significant solvatochromic shift.

Table S2. Fluorescence excitation and emission maxima found for ThT, **E265**, and **E736**.

	$\lambda_{ex, free}$	$\lambda_{em, free}$	$\lambda_{ex, A\beta(1-42)}$	$\lambda_{em, A\beta(1-42)}$
**ThT**	346 nm	506 nm	440 nm	487 nm
**E265**	425 nm	480 nm	425 nm	475 nm
**E736**	333 nm	422 nm	333 nm	415 nm

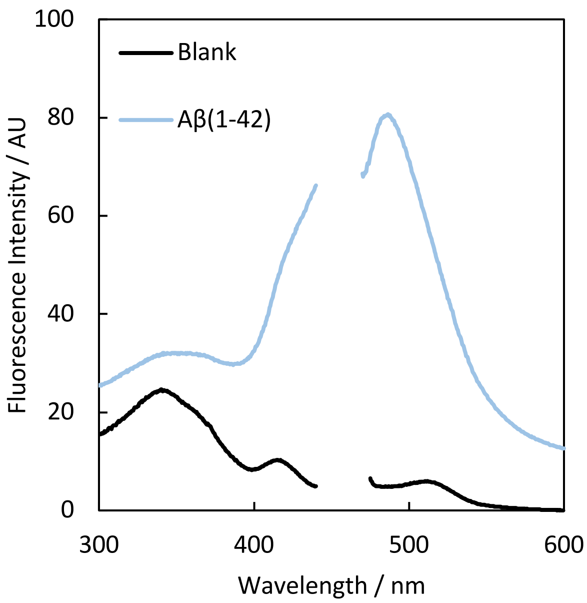

Figure S14. Fluorescence spectra (λ_{ex} = 440 nm, λ_{em} = 485 nm) of ThT (1.0 µM) in the presence and absence of Aβ(1-42) fibrils (500 nM).

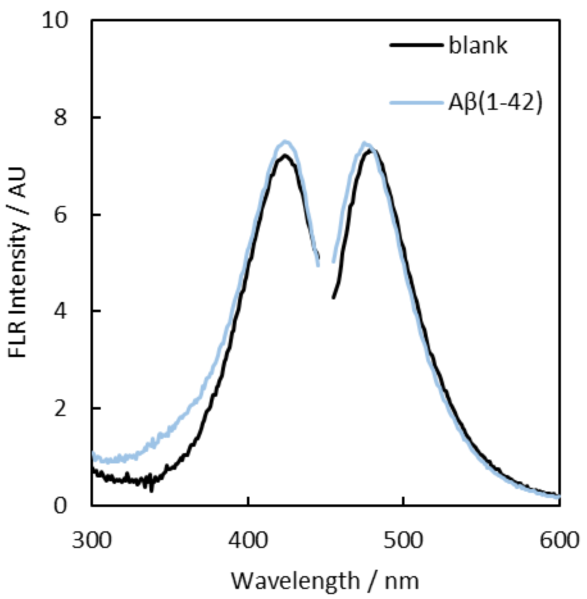

Figure S15. Fluorescence spectra (λ_{ex} = 425 nm, λ_{em} = 475 nm) of **E265** (1.0 µM) in the presence and absence of Aβ(1-42) fibrils (500 nM).

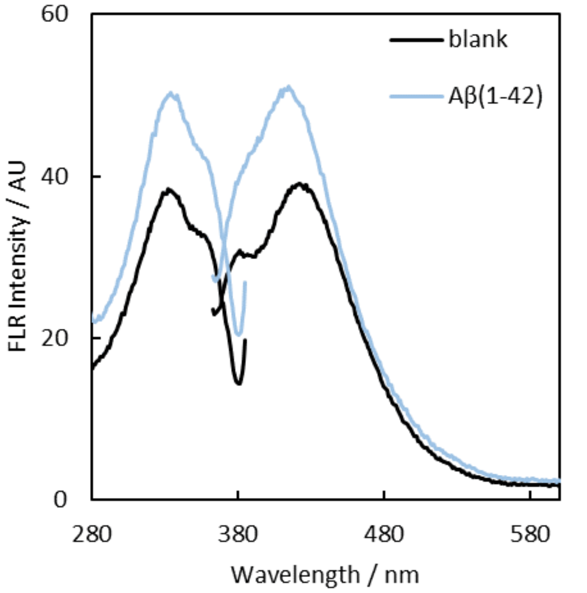

Figure S16. Fluorescence spectra (λ_{ex} = 333 nm, λ_{em} = 415 nm) of **E736** (1.0 µM) in the presence and absence of Aβ(1-42) fibrils (500 nM).

Saturation Binding Assays

For saturation binding assays, a solution of ThT, **E265**, or **E736** (10 µM) and Aβ(1-42) fibrils (500 nM) in 1xPBS (pH 7.4) was titrated into a solution of Aβ(1-42) fibrils (500 nM) in 1xPBS (pH 7.4) at 298 K. Fluorescence emission spectra were taken for ThT at λ_{ex} = 440 nm (λ_{em} = 470-600 nm), for **E265** at λ_{ex} = 425 nm (λ_{em} = 460-600 nm), and for **E736** at λ_{ex} = 333 nm (λ_{em} = 375-600 nm). The presence of fibrils in the ligand solution ensures that there is no dilution of the protein upon titration.

Competition Binding Assays

For the competition binding assay screening, solutions of competing ligand (82 μM), ThT (1.0 μM), and Aβ(1-42) fibrils (250 nM) were titrated into a solution of ThT (1.0 μM) and Aβ(1-42) fibrils (250 nM) in 1xPBS (pH 7.4) at 298 K. Fluorescence emission spectra were taken for ThT at λ_{ex} = 440 nm (λ_{em} = 470-600 nm).

For the higher-resolution competition binding assays of **E163**, **E363**, **E197**, **E363**, and **E704**, solutions of competing ligand (82 μM), ThT (1.0 μM), and Aβ(1-42) fibrils (250 nM) were titrated into a solution of ThT (1.0 μM) and Aβ(1-42) fibrils (250 nM) in 1xPBS (pH 7.4) at 298 K. Fluorescence emission spectra were taken for ThT at λ_{ex} = 440 nm (λ_{em} = 470-600 nm).

Fluorescence Anisotropy Assays

For fluorescence anisotropy blank assays, solutions of ligand (10 μM) in 1xPBS (pH 7.4) were titrated into a solution of 1xPBS (pH 7.4) at 298 K. Fluorescence emission spectra were taken for **E265** at λ_{ex} = 425 nm (λ_{em} = 465-485 nm), and for **E736** at λ_{ex} = 333 nm (λ_{em} = 405-425 nm). G-factors were calibrated using solutions of **E265** and **E736** in 1xPBS (20 μM, pH 7.4) for each titration respectively.

Ligand Dilution Series

Ligand dilution series were performed by repeating the above procedures in the absence of Aβ(1-42) fibrils.

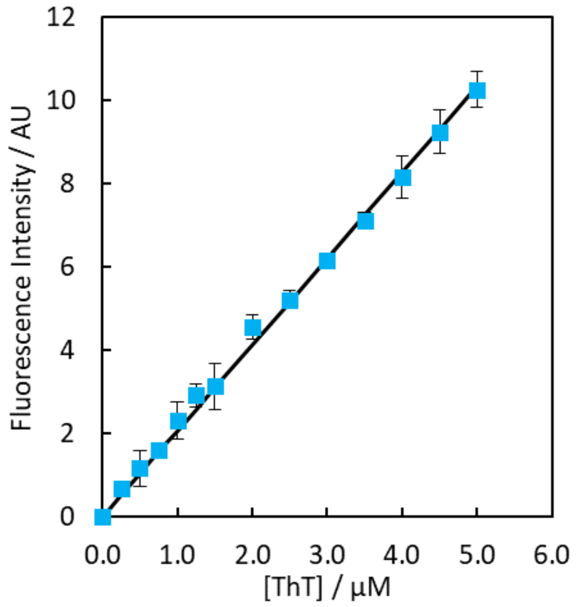

Figure S17: Dilution series of ThT into 1xPBS (pH 7.4, 25 °C). Spectra were recorded using λ_{ex} = 440 nm and monitoring emission at λ_{em} = 483 nm. The experimental measurements are shown as points (error bars represent the 95% confidence interval calculated from at least three independent experiments).The line of best fit has a slope of $(\mathbf{1.9} \pm \mathbf{0.2}) \times \mathbf{10^6}\ \boldsymbol{M^{-1}}$ and a y-intercept of 0.

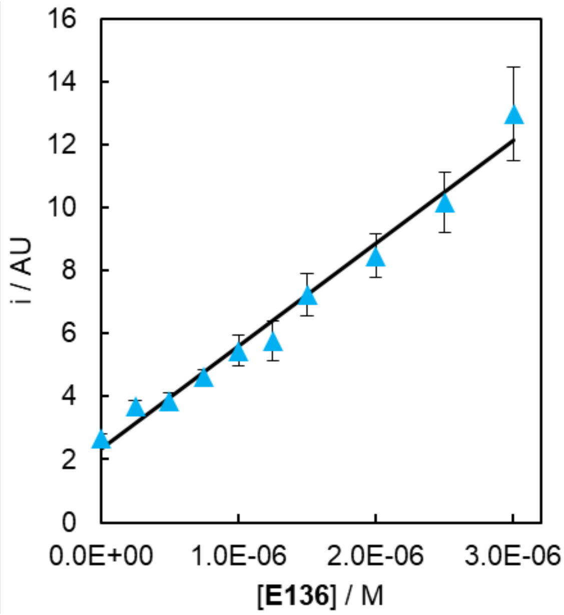

Figure S18. Dilution series of **E136** into a solution of ThT (1.0 µM) in 1xPBS (pH 7.4, 25 °C). Spectra were recorded using λ_{ex} = 440 nm and monitoring emission at λ_{em} = 483 nm. The experimental measurements are shown as points (error bars represent the 95% confidence interval calculated from at least three independent experiments).The line of best fit has a slope of $(\mathbf{3.3} \pm \mathbf{0.4}) \times \mathbf{10^{6}}\ \boldsymbol{M^{-1}}$ and a y-intercept of $\mathbf{2.3} \pm \mathbf{0.1}$.

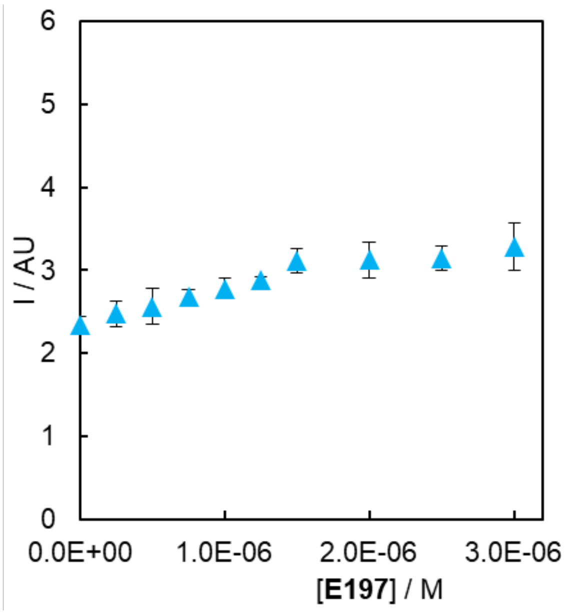

Figure S19. Dilution series of **E197** into a solution of ThT (1.0 µM) in 1xPBS (pH 7.4, 25 °C). Spectra were recorded using λ_{ex} = 440 nm and monitoring emission at λ_{em} = 483 nm. The experimental measurements are shown as points (error bars represent the 95% confidence interval calculated from at least three independent experiments).

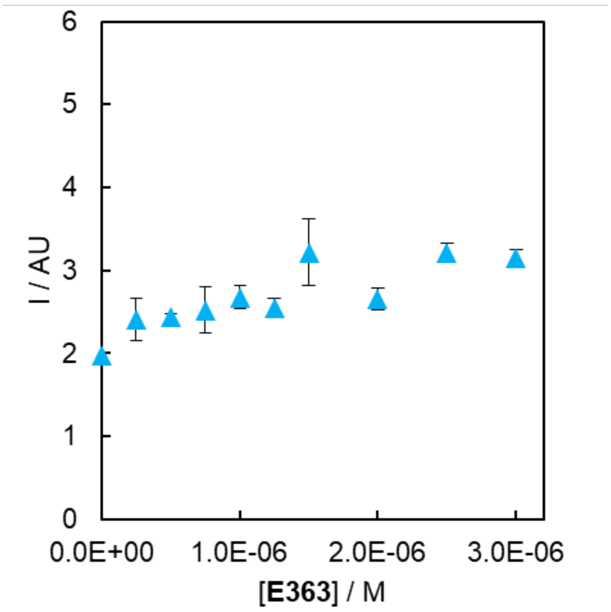

Figure S20. Dilution series of **E363** into a solution of ThT (1.0 µM) in 1xPBS (pH 7.4, 25 °C). Spectra were recorded using λ_{ex} = 440 nm and monitoring emission at λ_{em} = 483 nm. The experimental measurements are shown as points (error bars represent the 95% confidence interval calculated from at least three independent experiments).

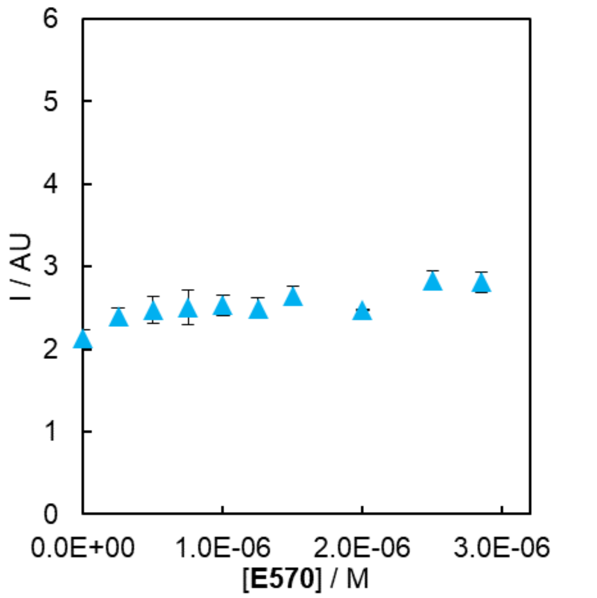

Figure S21. Dilution series of **E570** into a solution of ThT (1.0 µM) in 1xPBS (pH 7.4, 25 °C). Spectra were recorded using λ_{ex} = 440 nm and monitoring emission at λ_{em} = 483 nm. The experimental measurements are shown as points (error bars represent the 95% confidence interval calculated from at least three independent experiments).

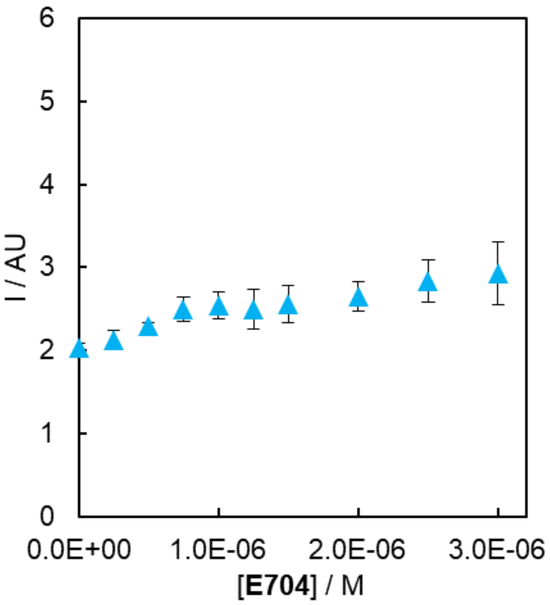

Figure S22. Dilution series of **E740** into a solution of ThT (1.0 μM) in 1xPBS (pH 7.4, 25 °C). Spectra were recorded using λ_{ex} = 440 nm and monitoring emission at λ_{em} = 483 nm. The experimental measurements are shown as points (error bars represent the 95% confidence interval calculated from at least three independent experiments).

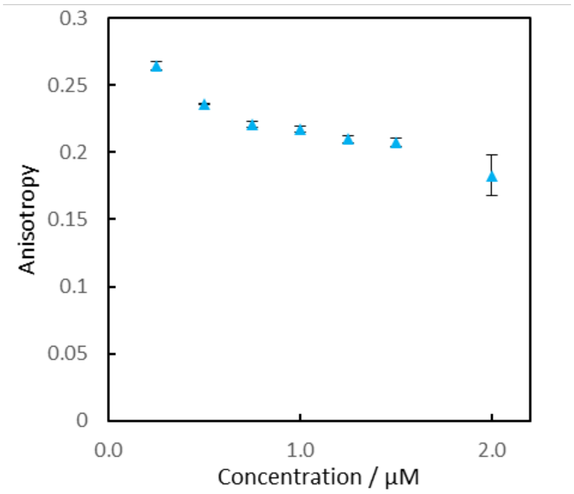

Figure S23. The measured fluorescence anisotropy from titrating a solution of E265 (10 μM) in 1xPBS (pH 7.4) into 1xPBS (pH 7.4, 298K). Spectra were recorded using λ_{ex} = 425 nm and monitoring emission at λ_{em} = 475 nm. Experimental measurements are shown as points (error bars represent the 95% confidence interval calculated from at least three independent experiments).

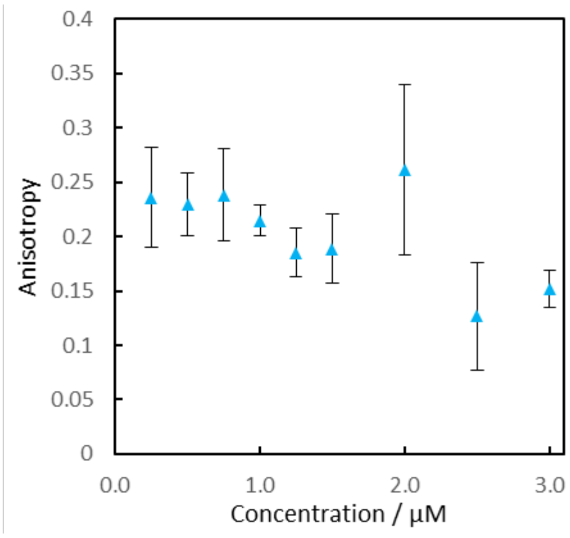

Figure S24. The measured fluorescence anisotropy from titrating a solution of E736 (10 µM) in 1xPBS (pH 7.4) into 1xPBS (pH 7.4, 298K). Spectra were recorded using λ_{ex} = 333 nm and monitoring emission at λ_{em} = 415 nm. Experimental measurements are shown as points (error bars represent the 95% confidence interval calculated from at least three independent experiments).

Data Fitting

Fluorescence spectra were analysed using a Microsoft Excel spreadsheet prepared by Professor Christopher Hunter. This spreadsheet fitted the measured fluorescence intensity at a fixed wavelength to a 1:1 binding isotherm using purpose-written VBA macros employing two algorithms, COGS and Simplex.

References

(1) Wang, R.; Fang, X.; Lu, Y.; Wang, S. The PDBbind Database: Collection of Binding Affinities for Protein−Ligand Complexes with Known Three-Dimensional Structures. *J. Med. Chem.* **2004**, *47* (12), 2977–2980.

(2) Mendez, D.; Gaulton, A.; Bento, A. P.; Chambers, J.; De Veij, M.; Félix, E.; Magariños, M. P.; Mosquera, J. F.; Mutowo, P.; Nowotka, M.; Gordillo-Marañón, M.; Hunter, F.; Junco, L.; Mugumbate, G.; Rodriguez-Lopez, M.; Atkinson, F.; Bosc, N.; Radoux, C. J.; Segura-Cabrera, A.; Hersey, A.; Leach, A. R. ChEMBL: Towards Direct Deposition of Bioassay Data. *Nucleic Acids Res.* **2019**, *47* (D1), D930–D940.

(3) RDKit: Open-Source Cheminformatics. http://www.rdkit.org.

(4) Chen, T.; Guestrin, C. XGBoost: A Scalable Tree Boosting System. In *Proceedings of the 22nd ACM SIGKDD International Conference on Knowledge Discovery and Data Mining*; KDD ’16; Association for Computing Machinery: New York, NY, USA, 2016; pp 785–794. https://doi.org/10.1145/2939672.2939785.

(5) Ke, G.; Meng, Q.; Finley, T.; Wang, T.; Chen, W.; Ma, W.; Ye, Q.; Liu, T.-Y. LightGBM: A Highly Efficient Gradient Boosting Decision Tree. In *Advances in Neural Information Processing Systems*; Curran Associates, Inc., 2017; Vol. 30.

(6) Freund, Y.; Schapire, R. E. A Decision-Theoretic Generalization of On-Line Learning and an Application to Boosting. *J. Comput. Syst. Sci.* **1997**, *55* (1), 119–139.

(7) Akiba, T.; Sano, S.; Yanase, T.; Ohta, T.; Koyama, M. Optuna: A Next-Generation Hyperparameter Optimization Framework. arXiv July 25, 2019.

(8) Sterling, T.; Irwin, J. J. ZINC 15 – Ligand Discovery for Everyone. *J. Chem. Inf. Model.* **2015**, *55* (11), 2324–2337.

(9) Tanimoto, T. *An Elementary Mathematical Theory of Classification and Prediction.*; Internal IBM Technical Report; 1958.
(10) Jaccard, P. The Distribution of the Flora in the Alpine Zone.1. *New Phytol.* **1912**, *11* (2), 37–50.
(11) Sorensen, T. A Method of Establishing Groups of Equal Amplitudes in Plant Sociology Based on Similarity of Species Content and Its Application to Analyses of the Vegetation on Danish Commons. *K. Dan. Vidensk. Selsk. Biol. Skr.* **1948**, *5*, 134.
(12) Dice, L. R. Measures of the Amount of Ecologic Association Between Species. *Ecology* **1945**, *26* (3), 297–302.
(13) Teplow, D. B. Preparation of Amyloid β-Protein for Structural and Functional Studies. *Methods Enzymol.* **2006**, *413*, 20–33.
(14) Zhang, J.; Konsmo, A.; Sandberg, A.; Wu, X.; Nyströ, S.; Obermü, U.; Wegenast-Braun, B. M.; Konradsson, P.; Lindgren, M.; Hammarströ, P. Phenolic Bis-Styrylbenzo[c]-1,2,5-Thiadiazoles as Probes for Fluorescence Microscopy Mapping of Aβ Plaque Heterogeneity. *J. Med. Chem.* **2019**, *62*, 2038–2048.

FBH Database

Table S3. The FBH database collected from the literature and used in model development. The "K_i / nM" column lists the dissociation constant measured of the compound in a competition assay. The "Error / nM" column gives the error in this measurement reported in the publication. The "K_i against" column lists the reporting ligand used in the competition assay.

Compound	SMILES	DOI	K_i / nM	Error / nM	K_i against
CG	OC(C=C1)=C(C(O)=O)C=C1/N=N/C2=CC=C(C3=CC=C(/N=N/C4=CC(C(O)=O)=C(O)C=C4)C=C3)C=C2	10.1021/jm010045q	>1000		[^{125}I]TZDM
IMSB	O=C(O)C1=CC(/C=C/C2=CC=C(/C=C/C3=CC(C(O)=O)=C(OC)C=C3)C(I)=C2)=CC=C1OC	10.1021/jm010045q	>1000		[^{125}I]TZDM
ThT	CN(C)C(C=C1)=CC=C1C2=[N+](C)C3=CC=C(O)C=C3S2	10.1021/jm010045q	294	40	[^{125}I]TZDM
Zhuang16a	BrC1=CC(SC(C2=CC=C(N(C)C)C=C2)=N3)=C3C=C1	10.1021/jm010045q	0.8	0.03	[^{125}I]TZDM
Zhuang16b	BrC1=CC(SC(C2=CC=C(N3CCN(C)CC3)C=C2)=N4)=C4C=C1	10.1021/jm010045q	5	0.8	[^{125}I]TZDM
TZDM	IC1=CC(SC(C2=CC=C(N(C)C)C=C2)=N3)=C3C=C1	10.1021/jm010045q	2.2	0.4	[^{125}I]TZDM
TZPI	IC1=CC(SC(C2=CC=C(N3CCN(C)CC3)C=C2)=N4)=C4C=C1	10.1021/jm010045q	6.4	0.7	[^{125}I]TZDM
Cui2a	CN(C)C1=CC=C(/C=C(C#N)/C#N)C=C1	10.1021/ja4052922	>10000		[^{125}I]IMPY
Cui2b	CN(C)C1=CC=C(/C=C/C=C(C#N)/C#N)C=C1	10.1021/ja4052922	518.8	29.3	[^{125}I]IMPY
Cui2c	CN(C)C1=CC=C(/C=C/C=C/C=C(C#N)/C#N)C=C1	10.1021/ja4052922	36.9	6.8	[^{125}I]IMPY
Fu3a	CN(C)C1=CC(C=CC(/C=C(C#N)/C#N)=C2)=C2C=C1	10.1021/acs.jmedchem.5b00861	156.4	15.8	[^{125}I]IMPY
Fu3b	CN(C)C1=CC(C=CC(/C=C/C=C(C#N)/C#N)=C2)=C2C=C1	10.1021/acs.jmedchem.5b00861	96.7	6.7	[^{125}I]IMPY
Fu3c	CN(C)C1=CC(C=CC(/C=C/C=C/C=C(C#N)/C#N)=C2)=C2C=C1	10.1021/acs.jmedchem.5b00861	32.7	3.4	[^{125}I]IMPY
Fu3d	CN(C)C1=CC(C=CC(/C=C/C=C/C=C/C=C(C#N)/C#N)=C2)=C2C=C1	10.1021/acs.jmedchem.5b00861	197.4	43	[^{125}I]IMPY
Fu3e	CN(C)C1=CC(C=CC(/C=C/C=C/C=C/C=C/C=C(C#N)/C#N)=C2)=C2C=C1	10.1021/acs.jmedchem.5b00861	>1000		[^{125}I]IMPY
MAAD-1	CN(C)C1=CC=C(/C=C/C=C2C(OC(C)(C)OC\2=O)=O)C=C1	10.1039/c4cc04907a	8007.46	462.69	[^{125}I]IMPY
DMDAD-1	CN(C)C1=CC=C(/C=C/C=C2C(CC(C)(C)CC\2=O)=O)C=C1	10.1039/c4cc04907a	2775.75	1.12	[^{125}I]IMPY
MCAAD-1	CN(C)C1=CC=C(/C=C/C=C(C(OC)=O)/C#N)C=C1	10.1039/c4cc04907a	5221.64	723.35	[^{125}I]IMPY
DMMAD-1	CN(C)C1=CC=C(/C=C/C=C(C(OC)=O)/C(OC)=O)C=C1	10.1039/c4cc04907a	7989.18	592.91	[^{125}I]IMPY
MAAD-2	CN(C)C1=CC=C(/C=C/C=C/C=C2C(OC(C)(C)OC\2=O)=O)C=C1	10.1039/c4cc04907a	767.16	109.98	[^{125}I]IMPY
DMDAD-2	CN(C)C1=CC=C(/C=C/C=C/C=C2C(CC(C)(C)CC\2=O)=O)C=C1	10.1039/c4cc04907a	592.09	108.97	[^{125}I]IMPY
MCAAD-2	CN(C)C1=CC=C(/C=C/C=C/C=C(C(OC)=O)/C#N)C=C1	10.1039/c4cc04907a	544.37	71.6	[^{125}I]IMPY
DMMAD-2	CN(C)C1=CC=C(/C=C/C=C/C=C(C(OC)=O)/C(OC)=O)C=C1	10.1039/c4cc04907a	712.46	156.52	[^{125}I]IMPY

Compound	SMILES	DOI	K_i / nM	Error / nM	K_i against
MAAD-3	CN(C)C1=CC=C(/C=C/C=C/C=C/C=C2C(OC(C)(C)OC\2=O)=O)C=C1	10.1039/c4cc04907a	354.3	43.5	[^{125}I]IMPY
DMDAD-3	CN(C)C1=CC=C(/C=C/C=C/C=C/C=C2C(CC(C)(C)CC\2=O)=O)C=C1	10.1039/c4cc04907a	645.2	77.2	[^{125}I]IMPY
MCAAD-3	CN(C)C1=CC=C(/C=C/C=C/C=C/C=C(C(OC)=O)/C#N)C=C1	10.1039/c4cc04907a	106	29.8	[^{125}I]IMPY
DMMAD-3	CN(C)C1=CC=C(/C=C/C=C/C=C/C=C(C(OC)=O)/C(OC)=O)C=C1	10.1039/c4cc04907a	652.6	143	[^{125}I]IMPY
PHC-1	CN(C)C1=CC=C(/C=C(C#N)/C#N)C=C1	10.1021/acs.analchem.7b02246	>10000		[^{125}I]IMPY
PHC-2	CN(C)C1=CC=C(/C=C/C=C(C#N)/C#N)C=C1	10.1021/acs.analchem.7b02246	518.8	29.3	[^{125}I]IMPY
PHC-3	CN(C)C1=CC=C(/C=C/C=C/C=C(C#N)/C#N)C=C1	10.1021/acs.analchem.7b02246	36.9	6.8	[^{125}I]IMPY
PHC-4	CN(C)C1=CC=C(/C=C/C=C/C=C/C=C(C#N)/C#N)C=C1	10.1021/acs.analchem.7b02246	14.1	6.4	[^{125}I]IMPY
PHC-5	CN(C)C1=CC=C(/C=C/C=C/C=C/C=C/C=C(C#N)/C#N)C=C1	10.1021/acs.analchem.7b02246	104.7	23.9	[^{125}I]IMPY
PHC-6	CN(C)C1=CC=C(/C=C/C=C/C=C/C=C/C=C/C=C(C#N)/C#N)C=C1	10.1021/acs.analchem.7b02246	835	175.9	[^{125}I]IMPY
PHC-7	CN(C)C1=CC=C(/C=C/C=C/C=C/C=C/C=C/C=C/C=C(C#N)/C#N)C=C1	10.1021/acs.analchem.7b02246	3061	687.7	[^{125}I]IMPY
PYC-1	CN(C)C1=CC=C(/C=C(C#N)/C#N)C=N1	10.1021/acs.analchem.7b02246	>10000		[^{125}I]IMPY
PYC-2	CN(C)C1=CC=C(/C=C/C=C(C#N)/C#N)C=N1	10.1021/acs.analchem.7b02246	>10000		[^{125}I]IMPY
PYC-3	CN(C)C1=CC=C(/C=C/C=C/C=C(C#N)/C#N)C=N1	10.1021/acs.analchem.7b02246	4984	99.7	[^{125}I]IMPY
PYC-4	CN(C)C1=CC=C(/C=C/C=C/C=C/C=C(C#N)/C#N)C=N1	10.1021/acs.analchem.7b02246	92.6	5.8	[^{125}I]IMPY
PYC-5	CN(C)C1=CC=C(/C=C/C=C/C=C/C=C/C=C(C#N)/C#N)C=N1	10.1021/acs.analchem.7b02246	26.6	3.9	[^{125}I]IMPY
PYC-6	CN(C)C1=CC=C(/C=C/C=C/C=C/C=C/C=C/C=C(C#N)/C#N)C=N1	10.1021/acs.analchem.7b02246	88.2	21.7	[^{125}I]IMPY
PYC-7	CN(C)C1=CC=C(/C=C/C=C/C=C/C=C/C=C/C=C/C=C(C#N)/C#N)C=N1	10.1021/acs.analchem.7b02246	603	138.7	[^{125}I]IMPY
PMC-1	CN(C)C1=NC=C(/C=C(C#N)/C#N)C=N1	10.1021/acs.analchem.7b02246	>10000		[^{125}I]IMPY
PMC-2	CN(C)C1=NC=C(/C=C/C=C(C#N)/C#N)C=N1	10.1021/acs.analchem.7b02246	>10000		[^{125}I]IMPY
PMC-3	CN(C)C1=NC=C(/C=C/C=C/C=C(C#N)/C#N)C=N1	10.1021/acs.analchem.7b02246	4340	1301	[^{125}I]IMPY
PMC-4	CN(C)C1=NC=C(/C=C/C=C/C=C/C=C(C#N)/C#N)C=N1	10.1021/acs.analchem.7b02246	978.2	252	[^{125}I]IMPY
PMC-5	CN(C)C1=NC=C(/C=C/C=C/C=C/C=C/C=C(C#N)/C#N)C=N1	10.1021/acs.analchem.7b02246	162.1	23.6	[^{125}I]IMPY
PMC-6	CN(C)C1=NC=C(/C=C/C=C/C=C/C=C/C=C/C=C(C#N)/C#N)C=N1	10.1021/acs.analchem.7b02246	20.6	5.1	[^{125}I]IMPY
PMC-7	CN(C)C1=NC=C(/C=C/C=C/C=C/C=C/C=C/C=C/C=C(C#N)/C#N)C=N1	10.1021/acs.analchem.7b02246	2655	425.8	[^{125}I]IMPY
BODIPY7	F[B-]1(F)N2C(C=C3[N+]1=C(C)C=C3C)=CC=C2/C=C/C4=CC=C(C5=CC=C(I)C=C5)S4	10.1016/j.bmcl.2010.05.027	108		[^{125}I]IMPY

Compound	SMILES	DOI	K_i / nM	Error / nM	K_i against
Curcumin	OC1=C(OC)C=C(/C=C/C(/C=C(O)/C=C/C2=CC(OC)=C(O)C=C2)=O)C=C1	10.1002/cmdc.200700218	107	48	[^{125}I]IMPY
Narlawar02	OC(C(OC)=C1)=CC=C1/C=C/C2=CC(/C=C/C3=CC(OC)=C(O)C=C3)=NO2	10.1002/cmdc.200700218	138	95	[^{125}I]IMPY
Narlawar03	COC1=CC(/C=C/C2=CC(/C=C/C3=CC(OC)=C(OCC(OC(C)(C)C)=O)C=C3)=NO2)=CC=C1OCC(OC(C)(C)C)=O	10.1002/cmdc.200700218	>1000		[^{125}I]IMPY
Narlawar04	COC1=CC(/C=C/C2=CC(/C=C/C3=CC(OC)=C(OCC(O)=O)C=C3)=NO2)=CC=C1OCC(O)=O	10.1002/cmdc.200700218	>1000		[^{125}I]IMPY
Narlawar05a	OC(C(OC)=C1)=CC=C1/C=C/C2=CC(/C=C/C3=CC(OC)=C(O)C=C3)=NN2C	10.1002/cmdc.200700218	476	67	[^{125}I]IMPY
Narlawar05b	OC(C(OC)=C1)=CC=C1/C=C/C2=CC(/C=C/C3=CC(OC)=C(O)C=C3)=NN2CC4=CC=CC=C4	10.1002/cmdc.200700218	>1000		[^{125}I]IMPY
Narlawar05c	OC(C(OC)=C1)=CC=C1/C=C/C2=CC(/C=C/C3=CC(OC)=C(O)C=C3)=NN2C4=CC=CC=C4	10.1002/cmdc.200700218	>1000		[^{125}I]IMPY
Narlawar05d	OC(C(OC)=C1)=CC=C1/C=C/C2=CC(/C=C/C3=CC(OC)=C(O)C=C3)=NN2C4=CC=C([N+]([O-])=O)C=C4	10.1002/cmdc.200700218	>1000		[^{125}I]IMPY
Narlawar05e	OC(C(OC)=C1)=CC=C1/C=C/C2=CC(/C=C/C3=CC(OC)=C(O)C=C3)=NN2C4=NN=C(Cl)C=C4	10.1002/cmdc.200700218	>1000		[^{125}I]IMPY
Narlawar05f	OC(C(OC)=C1)=CC=C1/C=C/C2=CC(/C=C/C3=CC(OC)=C(O)C=C3)=NN2C4=CC=C(C)C=C4	10.1002/cmdc.200700218	>1000		[^{125}I]IMPY
Narlawar05g	OC(C(OC)=C1)=CC=C1/C=C/C2=CC(/C=C/C3=CC(OC)=C(O)C=C3)=NN2C4=CC=C(C(C)C)C=C4	10.1002/cmdc.200700218	>1000		[^{125}I]IMPY
BF-188	CNC(C=C1)=CC=C1/C=C/C=C/C2=NC3=C(N2)C=CC=C3	10.1007/s11307-013-0667-2	6.3		[^{3}H]PiB
PiB	CNC(C=C1)=CC=C1C2=NC3=CC=C(O)C=C3S2	10.1007/s11307-013-0667-2	5.7		[^{3}H]PiB
Ono2009-7a	O=C(/C=C/C1=CC=C(N(C)C)C=C1)C(C=C2)=CC=C2OCCF	10.1021/jm901057p	45.7	7.1	[^{125}I]DMIC
Ono2009-7b	O=C(/C=C/C1=CC=C(N(C)C)C=C1)C(C=C2)=CC=C2OCCOCCF	10.1021/jm901057p	20	2.5	[^{125}I]DMIC
Ono2009-7c	O=C(/C=C/C1=CC=C(N(C)C)C=C1)C(C=C2)=CC=C2OCCOCCOCCF	10.1021/jm901057p	38.9	4.2	[^{125}I]DMIC
Ono2009-11a	O=C(/C=C/C1=CC=C(N)C=C1)C(C=C2)=CC=C2OCCF	10.1021/jm901057p	678.9	21.7	[^{125}I]DMIC
Ono2009-11b	O=C(/C=C/C1=CC=C(N)C=C1)C(C=C2)=CC=C2OCCOCCF	10.1021/jm901057p	1048	114.3	[^{125}I]DMIC
Ono2009-11c	O=C(/C=C/C1=CC=C(N)C=C1)C(C=C2)=CC=C2OCCOCCOCCF	10.1021/jm901057p	790	132.1	[^{125}I]DMIC
Ono2009-12a	O=C(/C=C/C1=CC=C(NC)C=C1)C(C=C2)=CC=C2OCCF	10.1021/jm901057p	197.1	58.8	[^{125}I]DMIC
Ono2009-12b	O=C(/C=C/C1=CC=C(NC)C=C1)C(C=C2)=CC=C2OCCOCCF	10.1021/jm901057p	216.4	13.8	[^{125}I]DMIC
Ono2009-12c	O=C(/C=C/C1=CC=C(NC)C=C1)C(C=C2)=CC=C2OCCOCCOCCF	10.1021/jm901057p	470.9	100.4	[^{125}I]DMIC
Ono2009-13	FC1=CC=C(C(/C=C/C2=CC=C(N(C)C)C=C2)=O)C=C1	10.1021/jm901057p	49.8	6.2	[^{125}I]DMIC
Ono2009-15	FC1=CC=C(C(/C=C/C2=CC=C(N)C=C2)=O)C=C1	10.1021/jm901057p	663	88.3	[^{125}I]DMIC
Ono2009-16	FC1=CC=C(C(/C=C/C2=CC=C(NC)C=C2)=O)C=C1	10.1021/jm901057p	234.2	44	[^{125}I]DMIC

Compound	SMILES	DOI	K_i / nM	Error / nM	K_i against
DMIC	IC1=CC=C(C(/C=C/C2=CC=C(N(C)C)C=C2)=O)C=C1	10.1021/jm901057p	13.1	3	[125I]DMIC
IMPY	IC1=CN2C(C=C1)=NC(C3=CC=C(N(C)C)C=C3)=C2	10.1021/jm901057p	28	4.1	[125I]DMIC
Cui2011-01	FC1=CC=C(C(/C=C/C2=CC=C(NC=C3)C3=C2)=O)C=C1	10.1016/j.bmcl.2010.12.045	35.06	6.21	[125I]IMPY
Cui2011-02	ClC1=CC=C(C(/C=C/C2=CC=C(NC=C3)C3=C2)=O)C=C1	10.1016/j.bmcl.2010.12.045	8.43	2.13	[125I]IMPY
Cui2011-03	BrC1=CC=C(C(/C=C/C2=CC=C(NC=C3)C3=C2)=O)C=C1	10.1016/j.bmcl.2010.12.045	8.96	0.92	[125I]IMPY
Cui2011-04	IC1=CC=C(C(/C=C/C2=CC=C(NC=C3)C3=C2)=O)C=C1	10.1016/j.bmcl.2010.12.045	8.22	1.46	[125I]IMPY
Cui2011-05	OC1=CC=C(C(/C=C/C2=CC=C(NC=C3)C3=C2)=O)C=C1	10.1016/j.bmcl.2010.12.045	>360		[125I]IMPY
Cui2011-06	COC1=CC=C(C(/C=C/C2=CC=C(NC=C3)C3=C2)=O)C=C1	10.1016/j.bmcl.2010.12.045	8.52	2.15	[125I]IMPY
Cui2011-07	NC1=CC=C(C(/C=C/C2=CC=C(NC=C3)C3=C2)=O)C=C1	10.1016/j.bmcl.2010.12.045	>1008		[125I]IMPY
Cui2011-08	CNC1=CC=C(C(/C=C/C2=CC=C(NC=C3)C3=C2)=O)C=C1	10.1016/j.bmcl.2010.12.045	51.09	7.71	[125I]IMPY
Cui2011-09	CN(C)C1=CC=C(C(/C=C/C2=CC=C(NC=C3)C3=C2)=O)C=C1	10.1016/j.bmcl.2010.12.045	5.17	0.32	[125I]IMPY
Cui2011-10	O=C(/C=C/C1=CC=C(NC=C2)C2=C1)C(C=C3)=CC4=C3C=C(OC)C=C4	10.1016/j.bmcl.2010.12.045	4.46	0.87	[125I]IMPY
DMIC	IC1=CC=C(C(/C=C/C2=CC=C(N(C)C)C=C2)=O)C=C1	10.1016/j.bmcl.2010.12.045	1.97	0.26	[125I]IMPY
FIAR	IC1=CC(C(/C(O2)=C/C3=CC=C(OCCF)C=C3)=O)=C2C=C1	10.1016/j.bmcl.2011.08.063	6.81		[125I]IMPY
Ono2005-10	IC1=CC(C(C=C(C2=CC=C(NC)C=C2)O3)=O)=C3C=C1	10.1021/jm050635e	30	3.4	[125I]Ono2005-11
Ono2005-11	IC1=CC(C(C=C(C2=CC=C(N(C)C)C=C2)O3)=O)=C3C=C1	10.1021/jm050635e	15.6	2.4	[125I]Ono2005-11
Ono2005-19	IC1=CC(C(C=C(C2=CC=C(OC)C=C2)O3)=O)=C3C=C1	10.1021/jm050635e	38.3	8.1	[125I]Ono2005-11
Ono2005-20	IC1=CC(C(C=C(C2=CC=C(O)C=C2)O3)=O)=C3C=C1	10.1021/jm050635e	77.2	9.2	[125I]Ono2005-11
ThT	OC1=CC=C([N+](C)=C(C2=CC=C(N(C)C)C=C2)S3)C3=C1	10.1021/jm050635e	>1000		[125I]Ono2005-11
CR	CC1=CC(C2=CC=C(/N=N/C3=CC(S(=O)([O-])=O)=C(C=CC=C4)C4=C3N)C(C)=C2)=CC=C1/N=N/C5=CC(S(=O)([O-])=O)=C(C=CC=C6)C6=C5N	10.1021/jm050635e	>1000		[125I]Ono2005-11
Ono2008-12	IC(C=C1)=CC=C1C2=NOC(C3=CC=C(N)C=C3)=N2	10.1016/j.bmc.2008.05.054	14.2	1.4	[125I]IMPY
Ono2008-13	IC(C=C1)=CC=C1C2=NOC(C3=CC=C(NC)C=C3)=N2	10.1016/j.bmc.2008.05.054	14.3	3.6	[125I]IMPY
Ono2008-14	IC(C=C1)=CC=C1C2=NOC(C3=CC=C(N(C)C)C=C3)=N2	10.1016/j.bmc.2008.05.054	15.4	1.4	[125I]IMPY
Ono2008-15	IC(C=C1)=CC=C1C2=NOC(C3=CC=C(OC)C=C3)=N2	10.1016/j.bmc.2008.05.054	4.3	2.1	[125I]IMPY
Ono2008-16	IC(C=C1)=CC=C1C2=NOC(C3=CC=C(O)C=C3)=N2	10.1016/j.bmc.2008.05.054	47.1	4.1	[125I]IMPY

Compound	SMILES	DOI	K_i / nM	Error / nM	K_i against
Watanabe2009-03 (1,3,4-DPOD-DM)	IC(C=C1)=CC=C1C2=NN=C(C3=CC=C(N(C)C)C=C3)O2	10.1016/j.bmc.2009.07.020	20.1	2.5	[^{125}I]IMPY
Watanabe2009-04	IC(C=C1)=CC=C1C2=NN=C(C3=CC=C(OC)C=C3)O2	10.1016/j.bmc.2009.07.020	46.1	12.6	[^{125}I]IMPY
Watanabe2009-06	IC(C=C1)=CC=C1C2=NN=C(C3=CC=C(O)C=C3)O2	10.1016/j.bmc.2009.07.020	229.6	47.3	[^{125}I]IMPY
Watanabe2009-07	IC(C=C1)=CC=C1C2=NN=C(C3=CC=C(OCCO)C=C3)O2	10.1016/j.bmc.2009.07.020	282.2	61.4	[^{125}I]IMPY
Watanabe2009-08	IC(C=C1)=CC=C1C2=NN=C(C3=CC=C(OCCOCCO)C=C3)O2	10.1016/j.bmc.2009.07.020	348.6	51.7	[^{125}I]IMPY
Watanabe2009-09	IC(C=C1)=CC=C1C2=NN=C(C3=CC=C(OCCOCCOCCO)C=C3)O2	10.1016/j.bmc.2009.07.020	257.7	34.8	[^{125}I]IMPY
Cui2012-24	CNC(C=C1)=CC=C1C2=NC3=CC(OCCOCCOCCF)=CC=C3O2	10.1021/jm300251n	9.3	2.2	[^{125}I]IMPY
Cui2012-32	CN(C)C(C=C1)=CC=C1C2=NC3=CC(OCCOCCOCCF)=CC=C3O2	10.1021/jm300251n	3.9	0.7	[^{125}I]IMPY
Cui2011-03	BrC1=CC=C(OC(C(C2=CC=C([N+]([O-])=O)C=C2)=O)=C3)C3=C1	10.1016/j.bmc.2011.04.049	>1000		[^{125}I]IMPY
Cui2011-04	IC1=CC=C(OC(C(C2=CC=C([N+]([O-])=O)C=C2)=O)=C3)C3=C1	10.1016/j.bmc.2011.04.049	>1000		[^{125}I]IMPY
Cui2011-05	BrC1=CC=C(OC(C(C2=CC=C(N)C=C2)=O)=C3)C3=C1	10.1016/j.bmc.2011.04.049	156.6	23.3	[^{125}I]IMPY
Cui2011-06	IC1=CC=C(OC(C(C2=CC=C(N)C=C2)=O)=C3)C3=C1	10.1016/j.bmc.2011.04.049	133.2	10.4	[^{125}I]IMPY
Cui2011-07	BrC1=CC=C(OC(C(C2=CC=C(NC)C=C2)=O)=C3)C3=C1	10.1016/j.bmc.2011.04.049	44.9	9.7	[^{125}I]IMPY
Cui2011-08	IC1=CC=C(OC(C(C2=CC=C(NC)C=C2)=O)=C3)C3=C1	10.1016/j.bmc.2011.04.049	34.5	7.3	[^{125}I]IMPY
Cui2011-09	BrC1=CC=C(OC(C(C2=CC=C(N(C)C)C=C2)=O)=C3)C3=C1	10.1016/j.bmc.2011.04.049	8.8	3.9	[^{125}I]IMPY
Cui2011-10	IC1=CC=C(OC(C(C2=CC=C(N(C)C)C=C2)=O)=C3)C3=C1	10.1016/j.bmc.2011.04.049	6.6	1	[^{125}I]IMPY
Re-BAT-Bp-1		10.1021/jm201513c	149.6	34.4	[^{125}I]IMPY
Re-BAT-Bp-2		10.1021/jm201513c	32.8	5.8	[^{125}I]IMPY
Re-BAT-Bp-3		10.1021/jm201513c	13.6	1.6	[^{125}I]IMPY
Ono2011b-10	FCCOCCOCCOC1=CC=C(OC(C2=CN=C(NC)C=C2)=C3)C3=C1	10.1021/jm200057u	2.41	0.11	[^{125}I]IMPY
Ono2011b-21	FCCOCCOCCOC1=CC=C(OC(C2=CC=C(NC)C=C2)=C3)C3=C1	10.1021/jm200057u	3.85	0.22	[^{125}I]IMPY
FPYBF-1	FCCOCCOCCOC1=CC=C(OC(C2=CN=C(N(C)C)C=C2)=C3)C3=C1	10.1021/jm200057u	0.95	0.21	[^{125}I]IMPY
FPHBF-1	FCCOCCOCCOC1=CC=C(OC(C2=CC=C(N(C)C)C=C2)=C3)C3=C1	10.1021/jm200057u	2	0.5	[^{125}I]IMPY
PiB	OC1=CC=C(N=C(C2=CC=C(NC)C=C2)S3)C3=C1	10.1021/jm200057u	9	1.31	[^{125}I]IMPY
Cheng2010-05	FC1=CC=C(OC(C2=CC=C(N)C=C2)=C3)C3=C1	10.1021/ml100082x	0.9	0.2	[^{125}I]IMPY

Compound	SMILES	DOI	K_i / nM	Error / nM	K_i against
Cheng2010-06	FC1=CC=C(OC(C2=CC=C(NC)C=C2)=C3)C3=C1	10.1021/ml100082x	0.53	0.05	[^{125}I]IMPY
Cheng2010-07	FC1=CC=C(OC(C2=CC=C(N(C)C)C=C2)=C3)C3=C1	10.1021/ml100082x	0.26	0.01	[^{125}I]IMPY
Cheng2010-17	CN(C)C(C=C1)=CC=C1C2=CC3=CC(OCCOCCOCCF)=CC=C3O2	10.1021/ml100082x	2	0.5	[^{125}I]IMPY
Cheng2010-21	CN(C)C(C=C1)=CC=C1C2=CC3=CC(OCC(CO)CF)=CC=C3O2	10.1021/ml100082x	1	0.3	[^{125}I]IMPY
FPYBF-1	FCCOCCOCCOC1=CC=C(OC(C2=CN=C(N(C)C)C=C2)=C3)C3=C1	10.1016/j.bmcl.2010.08.016	0.9		[^{125}I]IMPY
Cui2011c-12	IC1=CC(NC(C2=CC=C(N(C)C)C=C2)=N3)=C3C=C1	10.1016/j.nucmedbio.2010.09.012	9.8	1.2	[^{125}I]IMPY
Cui2011c-13	IC1=CC(NC(C2=CC=C(NC)C=C2)=N3)=C3C=C1	10.1016/j.nucmedbio.2010.09.012	40.3	4.5	[^{125}I]IMPY
Cui2011c-14	IC1=CC(NC(C2=CC=C(N)C=C2)=N3)=C3C=C1	10.1016/j.nucmedbio.2010.09.012	315.1	11.2	[^{125}I]IMPY
Cui2011c-15	IC1=CC(NC(C2=CC=C(OC)C=C2)=N3)=C3C=C1	10.1016/j.nucmedbio.2010.09.012	190.1	6.4	[^{125}I]IMPY
Cui2011c-16	IC1=CC(NC(C2=CC=C(O)C=C2)=N3)=C3C=C1	10.1016/j.nucmedbio.2010.09.012	901.5	22.1	[^{125}I]IMPY
IMPY	IC1=CN2C(C=C1)=NC(C3=CC=C(N(C)C)C=C3)=C2	10.1016/j.nucmedbio.2010.09.012	10.5	1	[^{125}I]IMPY
PiB	OC1=CC=C(N=C(C2=CC=C(NC[3H])C=C2)S3)C3=C1	10.1021/jm101129a	13		[^{3}H]PiB
Yousefi2011-05	CNC(C=C1)=CC=C1C2=CN(C3=CC=C(OC)C=C3S4)C4=N2	10.1021/jm101129a	5.8		[^{3}H]PiB
Cui2011d-03	BrC1=CC(N=CC(C2=CC=C(N(C)C)C=C2)=N3)=C3C=C1	10.1016/j.bmcl.2011.05.079	2.6	0.2	[^{125}I]IMPY
Cui2011d-05	IC1=CC(N=CC(C2=CC=C(N(C)C)C=C2)=N3)=C3C=C1	10.1016/j.bmcl.2011.05.079	4.1	0.7	[^{125}I]IMPY
Cui2011d-11	IC1=CC(N=CC(C2=CC=C(N)C=C2)=N3)=C3C=C1	10.1016/j.bmcl.2011.05.079	10.7	1.1	[^{125}I]IMPY
Cui2011d-12	IC1=CC(N=CC(C2=CC=C(NC)C=C2)=N3)=C3C=C1	10.1016/j.bmcl.2011.05.079	7.7	1.4	[^{125}I]IMPY
Cui2011-02	O=C(/C=C/C1=CC=CC=C1)/C=C/C2=CC=C(N(C)C)C=C2	10.1021/jm101404k	1.2	0.2	[^{125}I]IMPY
Cui2011-03	O=C(/C=C/C1=CC=C(F)C=C1)/C=C/C2=CC=C(N(C)C)C=C2	10.1021/jm101404k	3.7	0.9	[^{125}I]IMPY
Cui2011-04	O=C(/C=C/C1=CC=C(Cl)C=C1)/C=C/C2=CC=C(N(C)C)C=C2	10.1021/jm101404k	1.1	0.2	[^{125}I]IMPY
Cui2011-05	O=C(/C=C/C1=CC=C(Br)C=C1)/C=C/C2=CC=C(N(C)C)C=C2	10.1021/jm101404k	2.8	0.5	[^{125}I]IMPY
Cui2011-06	O=C(/C=C/C1=CC=C(I)C=C1)/C=C/C2=CC=C(N(C)C)C=C2	10.1021/jm101404k	0.9	0.2	[^{125}I]IMPY
Cui2011-07	O=C(/C=C/C1=CC=C(O)C=C1)/C=C/C2=CC=C(N(C)C)C=C2	10.1021/jm101404k	6.8	1.6	[^{125}I]IMPY
Cui2011-08	O=C(/C=C/C1=CC=C(OC)C=C1)/C=C/C2=CC=C(N(C)C)C=C2	10.1021/jm101404k	0.7	0.1	[^{125}I]IMPY
Cui2011-09	O=C(/C=C/C1=CC=C([N+]([O-])=O)C=C1)/C=C/C2=CC=C(N(C)C)C=C2	10.1021/jm101404k	5.2	1.1	[^{125}I]IMPY
Cui2011-10	O=C(/C=C/C1=CC=C(N)C=C1)/C=C/C2=CC=C(N(C)C)C=C2	10.1021/jm101404k	3.7	0.4	[^{125}I]IMPY

Compound	SMILES	DOI	K_i / nM	Error / nM	K_i against
Cui2011-11	O=C(/C=C/C1=CC=C(NC)C=C1)/C=C/C2=CC=C(N(C)C)C=C2	10.1021/jm101404k	2.9	0.5	[^{125}I]IMPY
Cui2011-12	O=C(/C=C/C1=CC=C(N(C)C)C=C1)/C=C/C2=CC=C(N(C)C)C=C2	10.1021/jm101404k	2.7	0.6	[^{125}I]IMPY
Cui2011-13	O=C(/C=C/C1=CC=C(C(O)=O)C=C1)/C=C/C2=CC=C(N(C)C)C=C2	10.1021/jm101404k	78.1	8.5	[^{125}I]IMPY
Cui2011-14	O=C(/C=C/C1=CC=C(C(F)(F)F)C=C1)/C=C/C2=CC=C(N(C)C)C=C2	10.1021/jm101404k	0.8	0.2	[^{125}I]IMPY
Cui2011-15	O=C(/C=C/C1=CC=C(C)C=C1)/C=C/C2=CC=C(N(C)C)C=C2	10.1021/jm101404k	1.3	0.2	[^{125}I]IMPY
Cui2011-16	O=C(/C=C/C1=CC=C(N(CC)(CC))C=C1)/C=C/C2=CC=C(N(C)C)C=C2	10.1021/jm101404k	15.7	3.9	[^{125}I]IMPY
Cui2011-17	O=C(/C=C/C1=CC=C(N(C2=CC=CC=C2)C3=CC=CC=C3)C=C1)/C=C/C4=CC=C(N(C)C)C=C4	10.1021/jm101404k	131.2	20.3	[^{125}I]IMPY
Cui2011-18	O=C(/C=C/C1=CC=C(OCC2=CC=CC=C2)C=C1)/C=C/C3=CC=C(N(C)C)C=C3	10.1021/jm101404k	2	0.2	[^{125}I]IMPY
Cui2011-19	O=C(/C=C/C1=CC=CC(F)=C1)/C=C/C2=CC=C(N(C)C)C=C2	10.1021/jm101404k	1.2	0.1	[^{125}I]IMPY
Cui2011-20	O=C(/C=C/C1=CC=CC(Br)=C1)/C=C/C2=CC=C(N(C)C)C=C2	10.1021/jm101404k	1.9	0.3	[^{125}I]IMPY
Cui2011-21	O=C(/C=C/C1=CC=CC(I)=C1)/C=C/C2=CC=C(N(C)C)C=C2	10.1021/jm101404k	5.5	1.7	[^{125}I]IMPY
Cui2011-22	O=C(/C=C/C1=CC=CC([N+]([O-])=O)=C1)/C=C/C2=CC=C(N(C)C)C=C2	10.1021/jm101404k	4	1.4	[^{125}I]IMPY
Cui2011-23	O=C(/C=C/C1=CC=CC(N)=C1)/C=C/C2=CC=C(N(C)C)C=C2	10.1021/jm101404k	13.7	3.4	[^{125}I]IMPY
Cui2011-24	O=C(/C=C/C1=CC=CC(NC)=C1)/C=C/C2=CC=C(N(C)C)C=C2	10.1021/jm101404k	8.1	1.4	[^{125}I]IMPY
Cui2011-25	O=C(/C=C/C1=CC=CC(N(C)C)=C1)/C=C/C2=CC=C(N(C)C)C=C2	10.1021/jm101404k	8	0.6	[^{125}I]IMPY
Cui2011-26	O=C(/C=C/C1=CC=CC=C1OCC2=CC=CC=C2)/C=C/C3=CC=C(N(C)C)C=C3	10.1021/jm101404k	18.6	8.1	[^{125}I]IMPY
Cui2011-27	O=C(/C=C/C1=CC=CC=C1Cl)/C=C/C2=CC=C(N(C)C)C=C2	10.1021/jm101404k	0.9	0.1	[^{125}I]IMPY
Cui2011-28	O=C(/C=C/C1=CC=CC=C1O)/C=C/C2=CC=C(N(C)C)C=C2	10.1021/jm101404k	28.2	5.1	[^{125}I]IMPY
Cui2011-29	O=C(/C=C/C1=CC=CC=C1[N+]([O-])=O)/C=C/C2=CC=C(N(C)C)C=C2	10.1021/jm101404k	18.8	3	[^{125}I]IMPY
Cui2011-30	O=C(/C=C/C1=CC=CC=C1N)/C=C/C2=CC=C(N(C)C)C=C2	10.1021/jm101404k	8.4	3	[^{125}I]IMPY
Cui2011-31	O=C(/C=C/C1=CC=CC=C1NC)/C=C/C2=CC=C(N(C)C)C=C2	10.1021/jm101404k	2636.5	140.8	[^{125}I]IMPY
Cui2011-32	O=C(/C=C/C1=C(Cl)C=CC=C1Cl)/C=C/C2=CC=C(N(C)C)C=C2	10.1021/jm101404k	18.5	0.9	[^{125}I]IMPY
Cui2011-33	O=C(/C=C/C1=C(Cl)C=CC=C1F)/C=C/C2=CC=C(N(C)C)C=C2	10.1021/jm101404k	9.2	1	[^{125}I]IMPY
Cui2011-34	O=C(/C=C/C1=CC(C(F)(F)F)=CC(C(F)(F)F)=C1)/C=C/C2=CC=C(N(C)C)C=C2	10.1021/jm101404k	4	0.5	[^{125}I]IMPY
Cui2011-35	O=C(/C=C/C1=CC=C(OC)C(OC)=C1)/C=C/C2=CC=C(N(C)C)C=C2	10.1021/jm101404k	5.4	0.9	[^{125}I]IMPY
Cui2011-36	O=C(/C=C/C1=CC(OC)=CC(OC)=C1)/C=C/C2=CC=C(N(C)C)C=C2	10.1021/jm101404k	4.7	2.1	[^{125}I]IMPY

Compound	SMILES	DOI	K_i / nM	Error / nM	K_i against
Cui2011-37	O=C(/C=C/C1=CC=C(OC)C=C1OC)/C=C/C2=CC=C(N(C)C)C=C2	10.1021/jm101404k	2.8	0.8	[^{125}I]IMPY
Cui2011-38	O=C(/C=C/C1=CC(OC)=C(OC)C(OC)=C1)/C=C/C2=CC=C(N(C)C)C=C2	10.1021/jm101404k	5.2	0.6	[^{125}I]IMPY
Cui2011-39	O=C(/C=C/C1=C(OC)C=C(OC)C=C1OC)/C=C/C2=CC=C(N(C)C)C=C2	10.1021/jm101404k	39.1	4.8	[^{125}I]IMPY
Cui2011-40	O=C(/C=C/C1=CC=C(OC2=CC=CC=C2)C(OC)=C1)/C=C/C3=CC=C(N(C)C)C=C3	10.1021/jm101404k	4.1	0.4	[^{125}I]IMPY
Cui2011-41	O=C(/C=C/C1=CC(Br)=CC=C1O)/C=C/C2=CC=C(N(C)C)C=C2	10.1021/jm101404k	16.2	5.1	[^{125}I]IMPY
Cui2011-42	O=C(/C=C/C1=CC=CC(OC2=CC=CC=C2)=C1)/C=C/C3=CC=C(N(C)C)C=C3	10.1021/jm101404k	3.8	1.1	[^{125}I]IMPY
Cui2011-43	O=C(/C=C/C1=CC([N+]([O-])=O)=CC=C1OCC2=CC=CC=C2)/C=C/C3=CC=C(N(C)C)C=C3	10.1021/jm101404k	33.3	3.8	[^{125}I]IMPY
Cui2011-44	O=C(/C=C/C1=CC=C(OC(C2=CC=CC=C2)(C3=CC=CC=C3)C4=CC=CC=C4)C=C1)/C=C/C5=CC=C(N(C)C)C=C5	10.1021/jm101404k	2.8	0.4	[^{125}I]IMPY
Cui2011-45	O=C(/C=C/C1=CN=C(Cl)C=C1)/C=C/C2=CC=C(N(C)C)C=C2	10.1021/jm101404k	2.2	0.3	[^{125}I]IMPY
Cui2011-46	O=C(/C=C/C1=CN=C(Br)C=C1)/C=C/C2=CC=C(N(C)C)C=C2	10.1021/jm101404k	2.3	0.1	[^{125}I]IMPY
Cui2011-47	O=C(/C=C/C1=CN=C(I)C=C1)/C=C/C2=CC=C(N(C)C)C=C2	10.1021/jm101404k	4.8	1.3	[^{125}I]IMPY
Cui2011-48	O=C(/C=C/C1=CC=CC=N1)/C=C/C2=CC=C(N(C)C)C=C2	10.1021/jm101404k	23	1.6	[^{125}I]IMPY
Cui2011-49	O=C(/C=C/C1=CC=CN1)/C=C/C2=CC=C(N(C)C)C=C2	10.1021/jm101404k	5.8	1.2	[^{125}I]IMPY
Cui2011-50	O=C(/C=C/C1=CC=CO1)/C=C/C2=CC=C(N(C)C)C=C2	10.1021/jm101404k	8.5	0.8	[^{125}I]IMPY
Cui2011-51	O=C(/C=C/C1=CC=CS1)/C=C/C2=CC=C(N(C)C)C=C2	10.1021/jm101404k	5.3	0.5	[^{125}I]IMPY
Cui2011-52	O=C(/C=C/C1=CC=C(Br)S1)/C=C/C2=CC=C(N(C)C)C=C2	10.1021/jm101404k	3.8	0.4	[^{125}I]IMPY
Cui2011-53	O=C(/C=C/C1=CC=C(C2=CC=C(Br)S2)S1)/C=C/C3=CC=C(N(C)C)C=C3	10.1021/jm101404k	11.7	1.1	[^{125}I]IMPY
Cui2011-54	O=C(/C=C/C1=CC=C(OCO2)C2=C1)/C=C/C3=CC=C(N(C)C)C=C3	10.1021/jm101404k	1.8	0.2	[^{125}I]IMPY
Cui2011-55	O=C(/C=C/C1=CC=C(NC=C2)C2=C1)/C=C/C3=CC=C(N(C)C)C=C3	10.1021/jm101404k	4.2	0.4	[^{125}I]IMPY
Cui2011-56	O=C(/C=C/C1=CC=C(C=C(OC)C=C2)C2=C1)/C=C/C3=CC=C(N(C)C)C=C3	10.1021/jm101404k	2.5	0.2	[^{125}I]IMPY
Cui2011-57	O=C(/C=C/c1cccc1)/C=C/C2=CC=C(N(C)C)C=C2.[][Fe][].c3cccc3	10.1021/jm101404k	21.1	1.2	[^{125}I]IMPY
Cui2011-58	O=C(/C=C/C1=CC=CC=C1)/C=C/C2=CC=CC=C2	10.1021/jm101404k	242.5	42.8	[^{125}I]IMPY
Cui2011-59	O=C(/C=C/C1=CC=C(Cl)C=C1)/C=C/C2=CC=C(Cl)C=C2	10.1021/jm101404k	9	1.2	[^{125}I]IMPY
Cui2011-60	O=C(/C=C/C1=CC=C(OC)C=C1)/C=C/C2=CC=C(OC)C=C2	10.1021/jm101404k	6.5	1.2	[^{125}I]IMPY
Cui2011-61	O=C(/C1=C/C2=CC=CC=C2)/C(CCC1)=C/C3=CC=CC=C3	10.1021/jm101404k	704.3	90.3	[^{125}I]IMPY

Compound	SMILES	DOI	K_i / nM	Error / nM	K_i against
Cui2011-62	O=C(/C1=C/C2=CC=C(Cl)C=C2)/C(CCC1)=C/C3=CC=C(Cl)C=C3	10.1021/jm101404k	11.4	0.8	[^{125}I]IMPY
Cui2011-63	O=C(/C1=C/C2=CC=C(OC)C=C2)/C(CCC1)=C/C3=CC=C(OC)C=C3	10.1021/jm101404k	24.8	2.5	[^{125}I]IMPY
Cui2011-64	O=C(/C1=C/C2=CC=C(N(C)C)C=C2)/C(CCC1)=C/C3=CC=C(N(C)C)C=C3	10.1021/jm101404k	1	0.3	[^{125}I]IMPY
Cui2011-70	O=C(/C=C/C1=CC=C(I)C=C1)/C=C/C2=CC=C(N)C=C2	10.1021/jm101404k	7	2.2	[^{125}I]IMPY
Cui2011-71	O=C(/C=C/C1=CC=C(I)C=C1)/C=C/C2=CC=C(NC)C=C2	10.1021/jm101404k	2.8	0.5	[^{125}I]IMPY
Cui2011-81	O=C(/C1=C/C2=CC=C(N(C)C)C=C2)/C(CCC1)=C/C3=CC=C(OCCF)C=C3	10.1021/jm101404k	5.6	2.5	[^{125}I]IMPY
Cui2011-82	O=C(/C1=C/C2=CC=C(N(C)C)C=C2)/C(CCC1)=C/C3=CC=C(OCCOCCF)C=C3	10.1021/jm101404k	4.4	1.3	[^{125}I]IMPY
Cui2011-83	O=C(/C1=C/C2=CC=C(N(C)C)C=C2)/C(CCC1)=C/C3=CC=C(OCCOCCOCCF)C=C3	10.1021/jm101404k	6.9	1.4	[^{125}I]IMPY
Cui2011-85	O=C(/C1=C/C2=CC=C(NC)C=C2)/C(CCC1)=C/C3=CC=C(OCCOCCOCCF)C=C3	10.1021/jm101404k	8.6	1.3	[^{125}I]IMPY
Ono2007b-04	NC1=CC=C(C(/C=C/C2=CC=C(I)C=C2)=O)C=C1	10.1016/j.bmc.2007.06.055	248	56	[^{125}I]DMIC
Ono2007b-07	CNC1=CC=C(C(/C=C/C2=CC=C(I)C=C2)=O)C=C1	10.1016/j.bmc.2007.06.055	23.9	3.6	[^{125}I]DMIC
Ono2007b-10	CN(C)C1=CC=C(C(/C=C/C2=CC=C(I)C=C2)=O)C=C1	10.1016/j.bmc.2007.06.055	13.3	1.9	[^{125}I]DMIC
Ono2007b-14	**O=C(/C=C/C1=CC=C(N(C)C)C=C1)C2=CC=C(I)S2**	10.1016/j.bmc.2007.06.055	3.9	0.4	[^{125}I]DMIC
Ono2007b-16	O=C(/C=C/C1=CC=CC=C1)C2=CC=C(I)C=C2	10.1016/j.bmc.2007.06.055	151	16	[^{125}I]DMIC
Ono2007b-17	O=C(/C=C/C1=CC=CO1)C2=CC=C(I)C=C2	10.1016/j.bmc.2007.06.055	908	212	[^{125}I]DMIC
Ono2007b-18	O=C(/C=C/C1=COC=C1)C2=CC=C(I)C=C2	10.1016/j.bmc.2007.06.055	125	9.2	[^{125}I]DMIC
Ono2007b-19	O=C(/C=C/C1=CC=CS1)C2=CC=C(I)C=C2	10.1016/j.bmc.2007.06.055	102	16	[^{125}I]DMIC
Ono2007b-20	O=C(/C=C/C1=CSC=C1)C2=CC=C(I)C=C2	10.1016/j.bmc.2007.06.055	93	11	[^{125}I]DMIC
Ono2007b-21	O=C(/C=C/C1=NC=CN1)C2=CC=C(I)C=C2	10.1016/j.bmc.2007.06.055	797	316	[^{125}I]DMIC
Ono2007b-22	O=C(/C=C/C1=NC=CS1)C2=CC=C(I)C=C2	10.1016/j.bmc.2007.06.055	>10000		[^{125}I]DMIC
Ono2007b-23	O=C(/C=C/C1=CC=C(N(C)C)O1)C2=CC=C(I)C=C2	10.1016/j.bmc.2007.06.055	1132	344	[^{125}I]DMIC
Ono2007b-24	O=C(/C=C/C1=CC=C(N(C)C)S1)C2=CC=C(I)C=C2	10.1016/j.bmc.2007.06.055	113	10	[^{125}I]DMIC
Ono2007b-25	O=C(/C=C/C1=CC=C(N(C)C)S1)C2=CC=C(I)S2	10.1016/j.bmc.2007.06.055	137	3.4	[^{125}I]DMIC
Ono2007b-26	O=C(/C=C/C1=CC=C(N(C)C)O1)C2=CC=C(I)S2	10.1016/j.bmc.2007.06.055	1608	85	[^{125}I]DMIC
Ono2007b-27	O=C(/C=C/C1=CC=C(N(C)C)C=C1)C2=CC=C(Br)O2	10.1016/j.bmc.2007.06.055	126	13	[^{125}I]DMIC
Ono2007b-28	O=C(/C=C/C1=CC=C(N(C)C)S1)C2=CC=C(Br)O2	10.1016/j.bmc.2007.06.055	2648	222	[^{125}I]DMIC

Compound	SMILES	DOI	K_i / nM	Error / nM	K_i against
Ono2007b-29	O=C(/C=C/C1=CC=C(N(C)C)O1)C2=CC=C(Br)O2	10.1016/j.bmc.2007.06.055	>10000		[^{125}I]DMIC
Ono2007b-31	O=C(/C=C/C1=CC=C(N)C=C1)C2=CC=C(I)S2	10.1016/j.bmc.2007.06.055	121	40	[^{125}I]DMIC
Ono2007b-32	O=C(/C=C/C1=CC=C(Br)S1)C2=CC=C(N)C=C2	10.1016/j.bmc.2007.06.055	476	48	[^{125}I]DMIC
Ono2007b-34	O=C(/C=C/C1=CC=C(NC)C=C1)C2=CC=C(I)S2	10.1016/j.bmc.2007.06.055	14.1	0.6	[^{125}I]DMIC
Ono2007b-35	O=C(/C=C/C1=CC=C(Br)S1)C2=CC=C(NC)C=C2	10.1016/j.bmc.2007.06.055	198	49	[^{125}I]DMIC
Ono2007b-36	O=C(/C=C/C1=CC=C(Br)S1)C2=CC=C(N(C)C)C=C2	10.1016/j.bmc.2007.06.055	106	7.1	[^{125}I]DMIC
CR	CC1=CC(C2=CC=C(/N=N/C3=CC(S(=O)([O-])=O)=C(C=CC=C4)C4=C3N)C(C)=C2)=CC=C1/N=N/C5=CC(S(=O)([O-])=O)=C(C=CC=C6)C6=C5N	10.1016/j.bmc.2007.06.055	>10000		[^{125}I]DMIC
ThT	OC1=CC=C([N+](C)=C(C2=CC=C(N(C)C)C=C2)S3)C3=C1	10.1016/j.bmc.2007.06.055	>10000		[^{125}I]DMIC
AIC	IC1=CC=C(C(/C=C/C2=CC=C(N)C=C2)=O)C=C1	10.1016/j.bmc.2007.06.055	105	12	[^{125}I]DMIC
IMC	IC1=CC=C(C(/C=C/C2=CC=C(NC)C=C2)=O)C=C1	10.1016/j.bmc.2007.06.055	6.3	1.6	[^{125}I]DMIC
DMIC	IC1=CC=C(C(/C=C/C2=CC=C(N(C)C)C=C2)=O)C=C1	10.1016/j.bmc.2007.06.055	2.9	0.3	[^{125}I]DMIC
Jia2016-S5	IC(C=C1)=CC=C1OC[C@H](O)COC(C=C2)=CC=C2OC	10.1021/acschemneuro.6b00155	34	2.7	[^{125}I]IMPY
Jia2016-R5	IC1=CC=C(OC[C@@H](O)COC2=CC=C(OC)C=C2)C=C1	10.1021/acschemneuro.6b00155	25	1.3	[^{125}I]IMPY
Jia2016-S8	IC(C=C1)=CC=C1OC[C@H](O)COC(C=C2)=CC=C2N(C)C	10.1021/acschemneuro.6b00155	45	13	[^{125}I]IMPY
Jia2016-R8	IC1=CC=C(OC[C@@H](O)COC2=CC=C(N(C)C)C=C2)C=C1	10.1021/acschemneuro.6b00155	15.8	11.5	[^{125}I]IMPY
IMPY	IC1=CN2C(C=C1)=NC(C3=CC=C(N(C)C)C=C3)=C2	10.1021/acschemneuro.6b00155	25.3	2.6	[^{125}I]IMPY
Ono2007d-04	IC1=CC=C(C(/C=C/C2=CC=C(N)C=C2)=O)C=C1	10.1016/j.bmc.2007.07.052	104.7	12	[^{125}I]DMIC
Ono2007d-07	IC1=CC=C(C(/C=C/C2=CC=C(NC)C=C2)=O)C=C1	10.1016/j.bmc.2007.07.052	6.3	1.6	[^{125}I]DMIC
Ono2007d-14	IC1=CC=C(C(/C=C/C2=CC=C(OC)C=C2)=O)C=C1	10.1016/j.bmc.2007.07.052	6.3	1.7	[^{125}I]DMIC
Ono2007d-16	IC1=CC=C(C(/C=C/C2=CC=C(O)C=C2)=O)C=C1	10.1016/j.bmc.2007.07.052	21.4	1.4	[^{125}I]DMIC
ThT	OC1=CC=C([N+](C)=C(C2=CC=C(N(C)C)C=C2)S3)C3=C1	10.1016/j.bmc.2007.07.052	>10000		[^{125}I]DMIC
CR	CC1=CC(C2=CC=C(/N=N/C3=CC(S(=O)([O-])=O)=C(C=CC=C4)C4=C3N)C(C)=C2)=CC=C1/N=N/C5=CC(S(=O)([O-])=O)=C(C=CC=C6)C6=C5N	10.1016/j.bmc.2007.07.052	>10000		[^{125}I]DMIC
Watanabe2010a-05	IC1=CC=C(NC(C2=CC=C(NC)C=C2)=C3)C3=C1	10.1016/j.bmc.2010.05.013	27	0.18	[^{125}I]IMPY
Watanabe2010a-11	IC1=CC=C(NC(C2=CC=C(N(C)C)C=C2)=C3)C3=C1	10.1016/j.bmc.2010.05.013	4.24	0.71	[^{125}I]IMPY
Watanabe2010a-14	IC1=CC=C(NC(C2=CC=C(OC)C=C2)=C3)C3=C1	10.1016/j.bmc.2010.05.013	20.2	5.15	[^{125}I]IMPY

Compound	SMILES	DOI	K_i / nM	Error / nM	K_i against
Watanabe2010a-15	IC1=CC=C(NC(C2=CC=C(O)C=C2)=C3)C3=C1	10.1016/j.bmc.2010.05.013	32.9	2.93	[^{125}I]IMPY
Watanabe2010a-16	IC1=CC=C(NC(C2=CC=C(OCCO)C=C2)=C3)C3=C1	10.1016/j.bmc.2010.05.013	25.9	5.13	[^{125}I]IMPY
Watanabe2010a-22	IC1=CC=C(N(C2=CC=C(N(C)C)C=C2)C=C3)C3=C1	10.1016/j.bmc.2010.05.013	>10000		[^{125}I]IMPY
Lee2011a-01	FC1=CC=C(N=C(C2=CN=C(N)C=C2)S3)C3=C1	10.1016/j.bmc.2011.03.029	26.2		[^{125}I]TZDM
Lee2011a-02	FC1=CC=C(N=C(C2=CN=C(NC)C=C2)S3)C3=C1	10.1016/j.bmc.2011.03.029	5.5	0.2	[^{125}I]TZDM
Lee2011a-03	FC1=CC=C(N=C(C2=CN=C(N(C)C)C=C2)S3)C3=C1	10.1016/j.bmc.2011.03.029	5.9	0.3	[^{125}I]TZDM
PiB	CNc1ccc(-c2nc3ccc(O)cc3s2)cc1	10.1016/j.bmc.2011.03.029	5.8	1.6	[^{125}I]TZDM
Fuchigami2013a-05	IC1=CC=C(C(NC(CCCN(CC)CC)C)=C(C=C(OC)C=C2)C2=N3)C3=C1	10.1016/j.ejmech.2012.12.020	353	81.5	[^{125}I]IMPY
Fuchigami2013a-12	IC1=CC=C(C(N(C)C)=C(C=C(OC)C=C2)C2=N3)C3=C1	10.1016/j.ejmech.2012.12.020	121	80.2	[^{125}I]IMPY
Fuchigami2013a-13	IC1=CC=C(C(NC)=C(C=C(OC)C=C2)C2=N3)C3=C1	10.1016/j.ejmech.2012.12.020	13.8	10.8	[^{125}I]IMPY
Fuchigami2013a-14	IC1=CC=C(C(N)=C(C=C(OC)C=C2)C2=N3)C3=C1	10.1016/j.ejmech.2012.12.020	83.6	30.7	[^{125}I]IMPY
Fuchigami2013a-25	IC1=CC=C(C(OC)=C(C=C(OC)C=C2)C2=N3)C3=C1	10.1016/j.ejmech.2012.12.020	28.9	7.97	[^{125}I]IMPY
Fuchigami2013a-26	IC1=CC=C(C(OC)=C(C=C(N(C)C)C=C2)C2=N3)C3=C1	10.1016/j.ejmech.2012.12.020	39.2	18.1	[^{125}I]IMPY
Fuchigami2013a-27	IC1=CC=C(C(OC)=C(C=CC=C2)C2=N3)C3=C1	10.1016/j.ejmech.2012.12.020	>10000		[^{125}I]IMPY
IMPY	IC1=CN2C(C=C1)=NC(C3=CC=C(N(C)C)C=C3)=C2	10.1016/j.ejmech.2012.12.020	8	1.1	[^{125}I]IMPY
PQ-1	FCCOC1=CC2=C(N=CC(C3=CC=C(N)C=C3)=N2)C=C1	10.1021/ml4000707	1180	370	[^{125}I]IMPY
PQ-2	FCCOC1=CC2=C(N=CC(C3=CC=C(NC)C=C3)=N2)C=C1	10.1021/ml4000707	758	83.8	[^{125}I]IMPY
PQ-3	FCCOC1=CC2=C(N=CC(C3=CC=C(N(C)C)C=C3)=N2)C=C1	10.1021/ml4000707	111	13.2	[^{125}I]IMPY
PQ-4	C1=CC2=C(N=CC(C3=CC=C(N)C=C3)=N2)C=C1OCCF	10.1021/ml4000707	242	29	[^{125}I]IMPY
PQ-5	C1=CC2=C(N=CC(C3=CC=C(NC)C=C3)=N2)C=C1OCCF	10.1021/ml4000707	15.7	1.28	[^{125}I]IMPY
PQ-6	C1=CC2=C(N=CC(C3=CC=C(N(C)C)C=C3)=N2)C=C1OCCF	10.1021/ml4000707	0.895	0.141	[^{125}I]IMPY
AV-45	FCCOCCOCCOC(C=C1)=NC=C1/C=C/C(C=C2)=CC=C2NC	10.1021/ml4000707	12.8	2.1	[^{125}I]IMPY
IMPY	IC1=CN2C(C=C1)=NC(C3=CC=C(N(C)C)C=C3)=C2	10.1021/ml4000707	7.21	1.23	[^{125}I]IMPY
Fu2013-01a	CN(CCF)C(C=C1)=CC=C1C2=CC3=CC=CC=C3N2	10.1016/j.bmc.2013.04.028	28.4	7.4	[^{125}I]IMPY
Fu2013-02a	CN(CCCF)C(C=C1)=CC=C1C2=CC3=CC=CC=C3N2	10.1016/j.bmc.2013.04.028	31.5	11.3	[^{125}I]IMPY
Fu2013-03a	CN(CCF)C(C=C1)=CC=C1C2=CC3=CC=CC=C3N(C)2	10.1016/j.bmc.2013.04.028	116.8	35.9	[^{125}I]IMPY

Compound	SMILES	DOI	K_i / nM	Error / nM	K_i against
Fu2013-04a	CN(CCCF)C(C=C1)=CC=C1C2=CC3=CC=CC=C3N(C)2	10.1016/j.bmc.2013.04.028	79.7	31.2	[^{125}I]IMPY
Fu2013-05a	N(CCF)C(C=C1)=CC=C1C2=CC3=CC=CC=C3N(C)2	10.1016/j.bmc.2013.04.028	1030.1	375.2	[^{125}I]IMPY
Fu2013-06a	N(CCCF)C(C=C1)=CC=C1C2=CC3=CC=CC=C3N(C)2	10.1016/j.bmc.2013.04.028	1097.8	171.6	[^{125}I]IMPY
Watanabe2013b-03	CN(C)c1ccc(-c2nnc(-c3ccc(I)cc3)o2)cn1	10.1039/C3MD00189J	24.8	2.81	[^{125}I]IMPY
Watanabe2013b-06	CN(C)c1ccc(-c2nnc(-c3cccc(I)c3)o2)cn1	10.1039/C3MD00189J	13.6	3.71	[^{125}I]IMPY
1,3,4-DPOD-DM	CN(C)c1ccc(-c2nnc(-c3ccc(I)cc3)o2)cc1	10.1039/C3MD00189J	3.91	0.98	[^{125}I]IMPY
IMPY	CN(C)c1ccc(-c2cn3cc(I)ccc3n2)cc1	10.1039/C3MD00189J	3.05	0.46	[^{125}I]IMPY
BOB-4	COc1ccc(OCc2ccc(I)cc2)cc1	10.1021/jm5004396	24.3	6.8	[^{125}I]Yang2014a-04
Yang2014a-05	COc1cccc(OCc2ccc(I)cc2)c1	10.1021/jm5004396	130.6	4.8	[^{125}I]Yang2014a-04
Yang2014a-06	COc1ccccc1OCc1ccc(I)cc1	10.1021/jm5004396	5164.8	410.6	[^{125}I]Yang2014a-04
Yang2014a-07	Oc1ccc(OCc2ccc(I)cc2)cc1	10.1021/jm5004396	113.4	23.8	[^{125}I]Yang2014a-04
Yang2014a-08	Oc1cccc(OCc2ccc(I)cc2)c1	10.1021/jm5004396	385.6	114.2	[^{125}I]Yang2014a-04
Yang2014a-09	Oc1ccccc1OCc1ccc(I)cc1	10.1021/jm5004396	2831.2	517	[^{125}I]Yang2014a-04
Yang2014a-10	Fc1ccc(OCc2ccc(I)cc2)cc1	10.1021/jm5004396	107.1	15.4	[^{125}I]Yang2014a-04
Yang2014a-11	Clc1ccc(OCc2ccc(I)cc2)cc1	10.1021/jm5004396	18.8	2.2	[^{125}I]Yang2014a-04
Yang2014a-12	Brc1ccc(OCc2ccc(I)cc2)cc1	10.1021/jm5004396	12	1	[^{125}I]Yang2014a-04
Yang2014a-13	Ic1ccc(COc2ccc(I)cc2)cc1	10.1021/jm5004396	21.9	2.1	[^{125}I]Yang2014a-04
Yang2014a-14	Ic1ccc(COc2ccccc2)cc1	10.1021/jm5004396	79.4	5.2	[^{125}I]Yang2014a-04
Yang2014a-15	CC(C)(C)c1ccc(OCc2ccc(I)cc2)cc1	10.1021/jm5004396	117.6	17.7	[^{125}I]Yang2014a-04
Yang2014a-16	Nc1ccc(OCc2ccc(I)cc2)cc1	10.1021/jm5004396	409.2	45	[^{125}I]Yang2014a-04
Yang2014a-17	Nc1cccc(OCc2ccc(I)cc2)c1	10.1021/jm5004396	1534.7	159.7	[^{125}I]Yang2014a-04
Yang2014a-18	Nc1ccccc1OCc1ccc(I)cc1	10.1021/jm5004396	1028	49	[^{125}I]Yang2014a-04
Yang2014a-19	CNc1ccc(OCc2ccc(I)cc2)cc1	10.1021/jm5004396	48.2	4.3	[^{125}I]Yang2014a-04
Yang2014a-20	CNc1cccc(OCc2ccc(I)cc2)c1	10.1021/jm5004396	593.6	78.2	[^{125}I]Yang2014a-04
Yang2014a-21	CNc1ccccc1OCc1ccc(I)cc1	10.1021/jm5004396	20662	2653	[^{125}I]Yang2014a-04
Yang2014a-22	CN(C)c1ccc(OCc2ccc(I)cc2)cc1	10.1021/jm5004396	17.6	1.6	[^{125}I]Yang2014a-04

Compound	SMILES	DOI	K_i / nM	Error / nM	K_i against
Yang2014a-23	CN(C)c1cccc(OCc2ccc(I)cc2)c1	10.1021/jm5004396	894.2	88	[125I]Yang2014a-04
Yang2014a-24	COc1ccc(COc2ccc(I)cc2)cc1	10.1021/jm5004396	49.4	3	[125I]Yang2014a-04
Yang2014a-25	COc1ccc(SCc2ccc(I)cc2)cc1	10.1021/jm5004396	530.2	109.4	[125I]Yang2014a-04
IMPY	CN(C)c1ccc(-c2cn3cc(I)ccc3n2)cc1	10.1021/jm5004396	32.2	2.1	[125I]BOB-4
PIB	CNc1ccc(-c2nc3ccc(O)cc3s2)cc1	10.1021/jm5004396	38.8	2.6	[125I]BOB-4
Yang2015a-05a	CN(C)c1ccc(COc2ccc(OCCF)cc2)cc1	10.1016/j.ejmech.2015.09.028	25.7	2.6	[125I]BOB-4
Yang2015a-05b	CN(C)c1ccc(COc2ccc(OCCOCCF)cc2)cc1	10.1016/j.ejmech.2015.09.028	191.1	38.4	[125I]BOB-4
Yang2015a-05c	CN(C)c1ccc(COc2ccc(OCCOCCOCCF)cc2)cc1	10.1016/j.ejmech.2015.09.028	357.7	22.2	[125I]BOB-4
Yang2015a-05d	COc1ccc(COc2ccc(OCCF)cc2)cc1	10.1016/j.ejmech.2015.09.028	118.5	34.6	[125I]BOB-4
Yang2015a-05e	COc1ccc(COc2ccc(OCCOCCF)cc2)cc1	10.1016/j.ejmech.2015.09.028	437.9	36.7	[125I]BOB-4
Yang2015a-05f	COc1ccc(COc2ccc(OCCOCCOCCF)cc2)cc1	10.1016/j.ejmech.2015.09.028	770.1	95	[125I]BOB-4
Yang2015a-09a	CN(C)c1ccc(OCc2ccc(OCCF)cc2)cc1	10.1016/j.ejmech.2015.09.028	21	4.9	[125I]BOB-4
Yang2015a-09b	COc1ccc(OCc2ccc(OCCF)cc2)cc1	10.1016/j.ejmech.2015.09.028	152.6	31.6	[125I]BOB-4
AV-45	CNc1ccc(/C=C/c2ccc(OCCOCCOCCF)nc2)cc1	10.1016/j.ejmech.2015.09.028	11.4	1.1	[125I]BOB-4
Yang2015b-07a	Ic1ccc(COc2ccc(OCCF)cc2)cc1	10.1038/srep12084	19.5	7.1	[125I]BOB-4
Yang2015b-12a	Ic1ccc(OCc2ccc(OCCF)cc2)cc1	10.1038/srep12084	23.9	7.9	[125I]BOB-4
Yu2012-4a	CNC1=CC=C(N=C(C2=CC=C(OCC[18F])C=C2)C=N3)C3=C1	10.1016/j.ejmech.2012.08.031	10		[125I]IMPY
Yu2012-4b	CNC1=CC=C(N=C(C2=CC=C(OCCOCCOCC[18F])C=C2)C=N3)C3=C1	10.1016/j.ejmech.2012.08.031	5.3		[125I]IMPY
Ono2013-07	IC1=CC(C=C(C2=CC=C(N)N=C2)O3)=C3C=C1	10.1371/journal.pone.0074104	10.3	1.48	[125I]IMPY
Ono2013-08	IC1=CC(C=C(C2=CC=C(NC)N=C2)O3)=C3C=C1	10.1371/journal.pone.0074104	2.94	0.22	[125I]IMPY
Ono2013-09	IC1=CC(C=C(C2=CC=C(N(C)C)N=C2)O3)=C3C=C1	10.1371/journal.pone.0074104	2.36	0.53	[125I]IMPY
Cui2010-06	C1=CC(SC(C2=CC=C(C3=CC=CS3)S2)=N4)=C4C=C1	10.1016/j.bmc.2010.02.002	3.94	0.06	[125I]TZDM
Cui2010-07	BrC1=CC(SC(C2=CC=C(C3=CC=CS3)S2)=N4)=C4C=C1	10.1016/j.bmc.2010.02.002	0.57	0.08	[125I]TZDM
Cui2010-08	COC1=CC(SC(C2=CC=C(C3=CC=CS3)S2)=N4)=C4C=C1	10.1016/j.bmc.2010.02.002	0.6	0.06	[125I]TZDM
Cui2010-09	COC1=CC(SC(C2=CC=C(C3=CC=C(Br)S3)S2)=N4)=C4C=C1	10.1016/j.bmc.2010.02.002	1.28	0.08	[125I]TZDM
Cui2010-10	COC1=CC(SC(C2=CC=C(C3=CC=C(I)S3)S2)=N4)=C4C=C1	10.1016/j.bmc.2010.02.002	0.61	0.09	[125I]TZDM

Compound	SMILES	DOI	K_i / nM	Error / nM	K_i against
Cui2010-13	IC1=CC(SC(C2=CC=C(C3=CC=CS3)S2)=N4)=C4C=C1	10.1016/j.bmc.2010.02.002	0.31	0.05	[^{125}I]TZDM
Chun2008-1a	CCOC(C1=CC(OC(C2=CC=C(N(C)C)C=C2)=N3)=C3C=C1)=O	10.5012/bkcs.2008.29.9.1765	0.61		[^{125}I]TZDM
Chun2008-1b	OC(C1=CC(OC(C2=CC=C(N(C)C)C=C2)=N3)=C3C=C1)=O	10.5012/bkcs.2008.29.9.1765	1.63		[^{125}I]TZDM
Chun2008-1c	CN(C)C(C=C1)=CC=C1C2=NC(C=C3)=C(O2)C=C3/C=C/C(O)=O	10.5012/bkcs.2008.29.9.1765	15.3		[^{125}I]TZDM
Chun2008-1d	CN(C)C(C=C1)=CC=C1C2=NC(C=C3)=C(O2)C=C3/C(C)=C/C(O)=O	10.5012/bkcs.2008.29.9.1765	1.18		[^{125}I]TZDM
Chun2008-1e	CN(C)C(C=C1)=CC=C1C2=NC(C=C3)=C(O2)C=C3/C=C(C#N)/C#N	10.5012/bkcs.2008.29.9.1765	0.8		[^{125}I]TZDM
Chun2008-1f	CN(C)C(C=C1)=CC=C1C2=NC(C=C3)=C(O2)C=C3/C(C)=C(C#N)/C#N	10.5012/bkcs.2008.29.9.1765	0.47		[^{125}I]TZDM
Li2016-06	FC1=C(CNC2=CC=CC=C2)C=CC(CNC3=CC=CC=C3)=C1	10.1021/acs.jmedchem.6b01063	2140	430	[^{125}I]IMPY
Li2016-07	FC1=C(CNC2=CC=C(C)C=C2)C=CC(CNC3=CC=C(C)C=C3)=C1	10.1021/acs.jmedchem.6b01063	71.1	3.6	[^{125}I]IMPY
Li2016-08	FC1=C(CNC2=CC=C(OC)C=C2)C=CC(CNC3=CC=C(OC)C=C3)=C1	10.1021/acs.jmedchem.6b01063	38.5	5.2	[^{125}I]IMPY
Li2016-09	FC1=C(CNC2=CC=C(Cl)C=C2)C=CC(CNC3=CC=C(Cl)C=C3)=C1	10.1021/acs.jmedchem.6b01063	240	45	[^{125}I]IMPY
Li2016-10	FC1=C(CNC2=CC=C(OCC)C=C2)C=CC(CNC3=CC=C(OCC)C=C3)=C1	10.1021/acs.jmedchem.6b01063	14.9	5.1	[^{125}I]IMPY
Li2016-11	FC1=C(CNC2=CC=C(CC)C=C2)C=CC(CNC3=CC=C(CC)C=C3)=C1	10.1021/acs.jmedchem.6b01063	18.5	1.2	[^{125}I]IMPY
Li2016-12	FC1=C(CNC2=CC=C(SC)C=C2)C=CC(CNC3=CC=C(SC)C=C3)=C1	10.1021/acs.jmedchem.6b01063	6.8	0.6	[^{125}I]IMPY
Li2016-13	FC1=C(CNC2=CC=CC=C2OC)C=CC(CNC3=C(OC)C=CC=C3)=C1	10.1021/acs.jmedchem.6b01063	>10000		[^{125}I]IMPY
AV-45		10.1021/acs.jmedchem.6b01063	8.9	1.5	[^{125}I]IMPY
IMPY	IC1=CN2C(C=C1)=NC(C3=CC=C(N(C)C)C=C3)=C2	10.1021/acs.jmedchem.6b01063	19.8	1.6	[^{125}I]IMPY
Li2014-07	NC1=CC=C(/C=C/C(/C=C/C2=CC=C(F)C=C2)=O)C=C1	10.1016/j.ejmech.2014.07.070	17.1	4.3	[^{125}I]IMPY
Li2014-08	CNC1=CC=C(/C=C/C(/C=C/C2=CC=C(F)C=C2)=O)C=C1	10.1016/j.ejmech.2014.07.070	6.4	3.2	[^{125}I]IMPY
Li2014-09	CN(C)C1=CC=C(/C=C/C(/C=C/C2=CC=C(F)C=C2)=O)C=C1	10.1016/j.ejmech.2014.07.070	3	0.6	[^{125}I]IMPY
Li2014-10	NC1=CC=C(/C=C/C(/C=C/C2=CN=C(F)C=C2)=O)C=C1	10.1016/j.ejmech.2014.07.070	135.2	58.1	[^{125}I]IMPY
Li2014-11	CNC1=CC=C(/C=C/C(/C=C/C2=CN=C(F)C=C2)=O)C=C1	10.1016/j.ejmech.2014.07.070	36.5	23.9	[^{125}I]IMPY
Li2014-12	CN(C)C1=CC=C(/C=C/C(/C=C/C2=CN=C(F)C=C2)=O)C=C1	10.1016/j.ejmech.2014.07.070	8.5	2	[^{125}I]IMPY
Li2014-13	NC1=CC=C(/C=C/C(/C=C/C2=CN=C(OCC)C=C2)=O)C=C1	10.1016/j.ejmech.2014.07.070	65.8	17.8	[^{125}I]IMPY
Li2014-14	CNC1=CC=C(/C=C/C(/C=C/C2=CN=C(OCC)C=C2)=O)C=C1	10.1016/j.ejmech.2014.07.070	14.2	5.2	[^{125}I]IMPY
Li2014-15	CN(C)C1=CC=C(/C=C/C(/C=C/C2=CN=C(OCC)C=C2)=O)C=C1	10.1016/j.ejmech.2014.07.070	7.6	3.9	[^{125}I]IMPY

Compound	SMILES	DOI	K_i / nM	Error / nM	K_i against
IMPY	IC1=CN2C(C=C1)=NC(C3=CC=C(N(C)C)C=C3)=C2	10.1016/j.ejmech.2014.07.070	11.5	2.5	[125I]IMPY
FA dimer	OC(/C=C/C(C=C1OC)=CC=C1OCCOC2=CC=C(/C=C/C(O)=O)C=C2OC)=O	10.1016/j.bmcl.2007.04.079	0.6		[125I]TZDM
Byeon2007-1a	OC(/C=C/C(C=C1OC)=CC=C1N(C)CCN(C)C2=CC=C(/C=C/C(O)=O)C=C2OC)=O	10.1016/j.bmcl.2007.04.079	0.72		[125I]TZDM
Byeon2007-1b	OC(/C=C/C(C=C1OC)=CC=C1N(CC2)CCN2C3=CC=C(/C=C/C(O)=O)C=C3OC)=O	10.1016/j.bmcl.2007.04.079	1.36		[125I]TZDM
Byeon2007-2a	O=C(O)/C=C/C1=CC(OC)=C(C=C1)OCCOC2=C(OC)C=C(N(C)C)C=C2	10.1016/j.bmcl.2007.04.079	1		[125I]TZDM
Byeon2007-2b	O=C(O)/C=C/C1=CC(OC)=C(C=C1)OCCOC2=C(OC)C=C(N(CC(O)=O)CC(O)=O)C=C2	10.1016/j.bmcl.2007.04.079	0.57		[125I]TZDM
Byeon2007-2c	O=C(O)/C=C/C1=CC(OC)=C(C=C1Br)OCCOC2=C(OC)C=C(N(C)C)C=C2	10.1016/j.bmcl.2007.04.079	0.83		[125I]TZDM
Byeon2007-2d	O=C(O)/C=C/C1=CC(OC)=C(C(Br)=C1)OCCOC2=C(OC)C=C(N(C)C)C=C2	10.1016/j.bmcl.2007.04.079	1.32		[125I]TZDM
Byeon2007-3a	C1(NCCNC2=NC(C=CC=C3)=C3S2)=NC4=C(C=CC=C4)S1	10.1016/j.bmcl.2007.04.079	0.71		[125I]TZDM
Byeon2007-3b	FC(C=C1)=CC2=C1N=C(S2)NCCNC3=NC(C=CC(F)=C4)=C4S3	10.1016/j.bmcl.2007.04.079	0.75		[125I]TZDM
Byeon2007-3c	BrC(C=C1)=CC2=C1N=C(S2)NCCNC3=NC(C=CC(Br)=C4)=C4S3	10.1016/j.bmcl.2007.04.079	1.11		[125I]TZDM
Byeon2007-3d	ClC(C=C1)=CC2=C1N=C(S2)NCCNC3=NC(C=CC(Cl)=C4)=C4S3	10.1016/j.bmcl.2007.04.079	0.96		[125I]TZDM
Byeon2007-3e	CS(C1=CC(S2)=C(C=C1)N=C2NCCNC3=NC4=C(C=C(S(C)(=O)=O)C=C4)S3)(=O)=O	10.1016/j.bmcl.2007.04.079	>10		[125I]TZDM
Byeon2007-3f	CC(C=C1)=CC2=C1N=C(S2)NCCNC3=NC(C=CC(C)=C4)=C4S3	10.1016/j.bmcl.2007.04.079	0.81		[125I]TZDM
Byeon2007-3g	NC(C=C1)=CC2=C1N=C(S2)NCCNC3=NC(C=CC(N)=C4)=C4S3	10.1016/j.bmcl.2007.04.079	0.69		[125I]TZDM
Byeon2007-4a	C1(N(C)CCN(C)C2=NC(C=CC=C3)=C3S2)=NC4=C(C=CC=C4)S1	10.1016/j.bmcl.2007.04.079	0.53		[125I]TZDM
Byeon2007-4b	FC(C=C1)=CC2=C1N=C(S2)N(C)CCN(C)C3=NC(C=CC(F)=C4)=C4S3	10.1016/j.bmcl.2007.04.079	1.37		[125I]TZDM
Byeon2007-4c	BrC(C=C1)=CC2=C1N=C(S2)N(C)CCN(C)C3=NC(C=CC(Br)=C4)=C4S3	10.1016/j.bmcl.2007.04.079	0.55		[125I]TZDM
Byeon2007-4d	ClC(C=C1)=CC2=C1N=C(S2)N(C)CCN(C)C3=NC(C=CC(Cl)=C4)=C4S3	10.1016/j.bmcl.2007.04.079	0.64		[125I]TZDM
Byeon2007-4e	CS(C1=CC(S2)=C(C=C1)N=C2N(C)CCN(C)C3=NC4=C(C=C(S(C)(=O)=O)C=C4)S3)(=O)=O	10.1016/j.bmcl.2007.04.079	0.55		[125I]TZDM
Byeon2007-4f	CC(C=C1)=CC2=C1N=C(S2)N(C)CCN(C)C3=NC(C=CC(C)=C4)=C4S3	10.1016/j.bmcl.2007.04.079	0.61		[125I]TZDM
Byeon2007-4g	NC(C=C1)=CC2=C1N=C(S2)N(C)CCN(C)C3=NC(C=CC(N)=C4)=C4S3	10.1016/j.bmcl.2007.04.079	0.69		[125I]TZDM
PiB	CNc1ccc(-c2nc3ccc(O)cc3s2)cc1	10.1016/j.bmcl.2007.04.079	0.77		[125I]TZDM
FA	OC1=C(OC)C=C(/C=C/C(O)=O)C=C1	10.1016/j.bmcl.2007.04.079	0.77		[125I]TZDM
Watanabe2015-07	IC1=CC=C(OC(C2=CN=C(N)C=C2)=N3)C3=C1	10.1039/c4ra10742j	138	18	[125I]IMPY
Watanabe2015-08	IC1=CC=C(OC(C2=CN=C(NC)C=C2)=N3)C3=C1	10.1039/c4ra10742j	26.7	2.54	[125I]IMPY

Compound	SMILES	DOI	K_i / nM	Error / nM	K_i against
Watanabe2015-09	IC1=CC=C(OC(C2=CN=C(N(C)C)C=C2)=N3)C3=C1	10.1039/c4ra10742j	6.9	1.14	[^{125}I]IMPY
IMPY	IC1=CN2C(C=C1)=NC(C3=CC=C(N(C)C)C=C3)=C2	10.1039/c4ra10742j	3.05	0.46	[^{125}I]IMPY
Fuchigami2015-13	IC1=CC(C(C=C(/C=C/C2=CC=CC=C2)O3)=O)=C3C=C1	10.1016/j.bmcl.2015.05.048	198	48	[^{125}I]Fuchigami2015-20
Fuchigami2015-14	IC1=CC(C(C=C(/C=C/C2=CC=C(OC)C=C2)O3)=O)=C3C=C1	10.1016/j.bmcl.2015.05.048	21.9	5.1	[^{125}I]Fuchigami2015-20
Fuchigami2015-15	IC1=CC(C(C=C(/C=C/C2=CC(OC)=C(OC)C=C2)O3)=O)=C3C=C1	10.1016/j.bmcl.2015.05.048	45.5	10.7	[^{125}I]Fuchigami2015-20
Fuchigami2015-16	IC1=CC(C(C=C(/C=C/C2=CC=C(O)C=C2)O3)=O)=C3C=C1	10.1016/j.bmcl.2015.05.048	121	20.8	[^{125}I]Fuchigami2015-20
Fuchigami2015-17	IC1=CC(C(C=C(/C=C/C2=CC=C(OCCO)C=C2)O3)=O)=C3C=C1	10.1016/j.bmcl.2015.05.048	446	100	[^{125}I]Fuchigami2015-20
Fuchigami2015-18	IC1=CC(C(C=C(/C=C/C2=CC=C(OCCOCCO)C=C2)O3)=O)=C3C=C1	10.1016/j.bmcl.2015.05.048	415	64.5	[^{125}I]Fuchigami2015-20
Fuchigami2015-19	IC1=CC(C(C=C(/C=C/C2=CC=C(OCCOCCOCCO)C=C2)O3)=O)=C3C=C1	10.1016/j.bmcl.2015.05.048	553	135	[^{125}I]Fuchigami2015-20
Fuchigami2015-20	IC1=CC(C(C=C(/C=C/C2=CC=C(N(C)C)C=C2)O3)=O)=C3C=C1	10.1016/j.bmcl.2015.05.048	21.8	3.35	[^{125}I]Fuchigami2015-20
Lee2008-18a	O=C1N(C2=CC=C(N)C=C2)CC3=C1C=CC(OCCOCCF)=C3	10.1016/j.bmcl.2008.05.019	0.61		[^{125}I]TZDM
Lee2008-18b	O=C1N(C2=CC=C(NC)C=C2)CC3=C1C=CC(OCCOCCF)=C3	10.1016/j.bmcl.2008.05.019	0.91		[^{125}I]TZDM
Lee2008-18c	O=C1N(C2=CC=C(N(C)C)C=C2)CC3=C1C=CC(OCCOCCF)=C3	10.1016/j.bmcl.2008.05.019	0.3		[^{125}I]TZDM
Lee2008-18d	O=C1N(C2=CC=C(N)C=C2)CC3=C1C=CC(OCCOCCOCCF)=C3	10.1016/j.bmcl.2008.05.019	0.4		[^{125}I]TZDM
Lee2008-18e	O=C1N(C2=CC=C(NC)C=C2)CC3=C1C=CC(OCCOCCOCCF)=C3	10.1016/j.bmcl.2008.05.019	0.51		[^{125}I]TZDM
Lee2008-18f	O=C1N(C2=CC=C(N(C)C)C=C2)CC3=C1C=CC(OCCOCCOCCF)=C3	10.1016/j.bmcl.2008.05.019	0.71		[^{125}I]TZDM
Lee2008-19a	O=C1N(C2=CC=C(N)C=C2)C(=O)C3=C1C=CC(OCCOCCF)=C3	10.1016/j.bmcl.2008.05.019	0.59		[^{125}I]TZDM
Lee2008-19b	O=C1N(C2=CC=C(NC)C=C2)C(=O)C3=C1C=CC(OCCOCCF)=C3	10.1016/j.bmcl.2008.05.019	0.6		[^{125}I]TZDM
Lee2008-19c	O=C1N(C2=CC=C(N(C)C)C=C2)C(=O)C3=C1C=CC(OCCOCCF)=C3	10.1016/j.bmcl.2008.05.019	0.69		[^{125}I]TZDM
Lee2008-19d	O=C1N(C2=CC=C(N)C=C2)C(=O)C3=C1C=CC(OCCOCCOCCF)=C3	10.1016/j.bmcl.2008.05.019	0.74		[^{125}I]TZDM
Lee2008-19e	O=C1N(C2=CC=C(NC)C=C2)C(=O)C3=C1C=CC(OCCOCCOCCF)=C3	10.1016/j.bmcl.2008.05.019	0.78		[^{125}I]TZDM
Lee2008-19f	O=C1N(C2=CC=C(N(C)C)C=C2)C(=O)C3=C1C=CC(OCCOCCOCCF)=C3	10.1016/j.bmcl.2008.05.019	0.85		[^{125}I]TZDM
Chang2006-2a	COC(C=C1)=CC=C1C2=CC3=CC=CC=C3S2	10.1016/j.nucmedbio.2006.06.006	0.52	0.14	[^{125}I]Chang2006-5
Chang2006-2b	OC(C=C1)=CC=C1C2=CC3=CC=CC=C3S2	10.1016/j.nucmedbio.2006.06.006	3.72	0.66	[^{125}I]Chang2006-5
Chang2006-2c	FCCOC(C=C1)=CC=C1C2=CC3=CC=CC=C3S2	10.1016/j.nucmedbio.2006.06.006	0.87	0.24	[^{125}I]Chang2006-5
Chang2006-2d	FCCCOC(C=C1)=CC=C1C2=CC3=CC=CC=C3S2	10.1016/j.nucmedbio.2006.06.006	0.73	0.44	[^{125}I]Chang2006-5

Compound	SMILES	DOI	K_i / nM	Error / nM	K_i against
Chang2006-3b	NC(C=C1)=CC=C1C2=CC3=CC=CC=C3S2	10.1016/j.nucmedbio.2006.06.006	6.5	1.88	[^{125}I]Chang2006-5
Chang2006-3c	CNC(C=C1)=CC=C1C2=CC3=CC=CC=C3S2	10.1016/j.nucmedbio.2006.06.006	0.72	0.16	[^{125}I]Chang2006-5
Chang2006-3d	CN(C)C(C=C1)=CC=C1C2=CC3=CC=CC=C3S2	10.1016/j.nucmedbio.2006.06.006	0.63	0.29	[^{125}I]Chang2006-5
Chang2006-3e	FCCNC(C=C1)=CC=C1C2=CC3=CC=CC=C3S2	10.1016/j.nucmedbio.2006.06.006	0.98	0.01	[^{125}I]Chang2006-5
Chang2006-3f	FCCCNC(C=C1)=CC=C1C2=CC3=CC=CC=C3S2	10.1016/j.nucmedbio.2006.06.006	0.77	0.41	[^{125}I]Chang2006-5
Chang2006-4	CNC(C=C1)=CC=C1C2=NC3=CC=CC=C3S2	10.1016/j.nucmedbio.2006.06.006	16.33	1.58	[^{125}I]Chang2006-5
Cui2012-2-28	CNC(N=C1)=CC=C1C2=NC3=CC(OCCOCCF)=CC=C3O2	10.1021/jm300973k	101.6	15.3	[^{125}I]IMPY
Cui2012-2-29	CNC(N=C1)=CC=C1C2=NC3=CC(OCCOCCOCCF)=CC=C3O2	10.1021/jm300973k	76.9	15.5	[^{125}I]IMPY
Cui2012-2-30	CN(C)C(N=C1)=CC=C1C2=NC3=CC(OCCOCCF)=CC=C3O2	10.1021/jm300973k	7.3	0.8	[^{125}I]IMPY
Cui2012-2-31	CN(C)C(N=C1)=CC=C1C2=NC3=CC(OCCOCCOCCF)=CC=C3O2	10.1021/jm300973k	9.9	0.5	[^{125}I]IMPY
Cui2012-2-32	CNC(N=C1)=CC=C1C2=NC3=CC(OCCF)=CC=C3O2	10.1021/jm300973k	8	3.2	[^{125}I]IMPY
Cui2012-2-33	CN(C)C(N=C1)=CC=C1C2=NC3=CC(OCCF)=CC=C3O2	10.1021/jm300973k	2.7	0.7	[^{125}I]IMPY
Cui2012-2-40	CNC(N=C1)=CC=C1C2=NC3=CC=C(OCCOCCF)C=C3S2	10.1021/jm300973k	29.7	7.1	[^{125}I]IMPY
Cui2012-2-41	CNC(N=C1)=CC=C1C2=NC3=CC=C(OCCOCCOCCF)C=C3S2	10.1021/jm300973k	9.3	0.1	[^{125}I]IMPY
Cui2012-2-42	CN(C)C(N=C1)=CC=C1C2=NC3=CC=C(OCCOCCF)C=C3S2	10.1021/jm300973k	4.6	0.6	[^{125}I]IMPY
Cui2012-2-43	CN(C)C(N=C1)=CC=C1C2=NC3=CC=C(OCCOCCOCCF)C=C3S2	10.1021/jm300973k	5.8	1.8	[^{125}I]IMPY
Cui2012-2-44	CNC(N=C1)=CC=C1C2=NC3=CC=C(OCCF)C=C3S2	10.1021/jm300973k	10.1	2.4	[^{125}I]IMPY
Cui2012-2-45	CN(C)C(N=C1)=CC=C1C2=NC3=CC=C(OCCF)C=C3S2	10.1021/jm300973k	2.7	0.6	[^{125}I]IMPY

Non-FBH Database

Table 4. Database containing other ligands that have been assayed against Aβ(1-42). The "K_d / nM" column lists the dissociation constant measured of the compound in a direct binding assay. The "K_i / nM" column lists the dissociation constant measured of the compound in a competition assay. The "Error / nM" column gives the error in either the K_d or K_i measurement reported in the publication. The "K_i against" column lists the reporting ligand used in the competition assay, if any. The "Two-site model" column identifies whether the reported binding constant was fitted using a two-site model.

Compound	SMILES	DOI	K_d / nM	K_i / nM	Error / nM	K_i against	Two-site model?
BTDSB	OC1=CC=C(/C=C/C(C2=NSN=C23)=CC=C3/C=C/C4=CC(C(O)=O)=C(O)C=C4)C=C1C(O)=O	10.1002/chem.201800501	93		5		
NSB	OC1=CC=C(/C=C/C2=CC(C=CC(/C=C/C3=CC(C(O)=O)=C(O)C=C3)=C4)=C4C=C2)C=C1C(O)=O	10.1002/chem.201800501	264		13		
QSB	OC1=CC=C(/C=C/C(C2=C3N=CC=N2)=CC=C3/C=C/C4=CC(C(O)=O)=C(O)C=C4)C=C1C(O)=O	10.1002/chem.201800501	124		7		
ThT	OC1=CC=C([N+](C)=C(C2=CC=C(N(C)C)C=C2)S3)C3=C1	10.1002/chem.201800501	665		66		
TSB	OC(C(C(O)=O)=C1)=CC=C1/C=C/C2=CC=C(/C=C/C3=CC(C(O)=O)=C(O)C=C3)S2	10.1002/chem.201800501	13		1		
X-34	OC1=CC=C(/C=C/C2=CC=C(/C=C/C3=CC(C(O)=O)=C(O)C=C3)C=C2)C=C1C(O)=O	10.1002/chem.201800501	299		21		
Sutharsan11	O=C(OCCOCCOCCOC)/C(C#N)=C/C1=CC(C=C2)=C(C=C1)C=C2N3CCCCC3	10.1002/cmdc.200900440	2500				
Sutharsan14	CN(C)C1=CC=C(/C=C(C#N)/C(OCC(CO)O)=O)C=C1	10.1002/cmdc.200900440	3300				
Sutharsan19	CN(C)C1=CC=C(/C=C/C2=CC=C(/C=C(C#N)/C(OCCOCCOCCOC)=O)C=C2)C=C1	10.1002/cmdc.200900440	1400				
Sutharsan8a	CN(C)C1=CC=C(/C=C(C#N)/C(OCCOCCOCCOC)=O)C=C1	10.1002/cmdc.200900440	2600				
Sutharsan8b	CN(C)C1=CC=C(/C=C(C#N)/C(OCCOCCOCCOC)=O)C(OC)=C1	10.1002/cmdc.200900440	5300				
Sutharsan8c	O=C(OCCOCCOCCOC)/C(C#N)=C/C(C=C1)=CC=C1N(CC)CC	10.1002/cmdc.200900440	4800				

Compound	SMILES	DOI	K_d / nM	K_i / nM	Error / nM	K_i against	Two-site model?
Sutharsan8d	O=C(OCCOCCOCCOC)/C(C#N)=C/C(C=C1)=CC=C1N(CCCC)CCCC	10.1002/cmdc.200900440	4400				
Py1SA	OC(C=C1)=C(C(O)=O)C=C1/C=C/C2=C3C4=C(C=CC5=C4C(C=C3)=CC=C5)C=C2	10.1002/cphc.201800823	310		29		
Py2SA	OC(C=C1)=C(C(O)=O)C=C1/C=C/C2=CC(C=CC3=C4C(C=C5)=CC=C3)=C4C5=C2	10.1002/cphc.201800823	48				Site 1
Py2SA	OC(C=C1)=C(C(O)=O)C=C1/C=C/C2=CC(C=CC3=C4C(C=C5)=CC=C3)=C4C5=C2	10.1002/cphc.201800823	360				Site 2
BF-227	[18F]CCOC1=CC=C2C(OC(/C=C/C3=CN=C(N(C)C)S3)=N2)=C1	10.1007/s00259-012-2261-2	1.72		0.83		Site 1
BF-227	[18F]CCOC1=CC=C2C(OC(/C=C/C3=CN=C(N(C)C)S3)=N2)=C1	10.1007/s00259-012-2261-2	56.1		25.1		Site 2
FDDNP	C/C(C1=CC(C=C2)=C(C=C1)C=C2N(C)CC[18F])=C(C#N)/C#N	10.1007/s00259-012-2261-2	5.52		1.97		
PiB	OC1=CC=C2C(SC(C3=CC=C(N[11CH3])C=C3)=N2)=C1	10.1007/s00259-012-2261-2	0.84		0.18		Site 1
PiB	OC1=CC=C2C(SC(C3=CC=C(N[11CH3])C=C3)=N2)=C1	10.1007/s00259-012-2261-2	60.6		8.32		Site 2
THK-523	[18F]CCOC1=CC=C(N=C(C2=CC=C(N)C=C2)C=C3)C3=C1	10.1007/s00259-012-2261-2	30.3		3.91		
[11C]MODAG-001	CN(C)C1=CC=C(C2=NNC(C3=CC=NC(Br)=C3)=C2)C=C1	10.1007/s00259-020-05133-x	20		10		
[125I]Ono2007-12	[125I]C1=CC(C(/C(O2)=C/C3=CC=C(N)C=C3)=O)=C2C=C1	10.1016/j.bbrc.2007.06.162	7.9		1.3		
Ono2007-12	IC1=CC(C(/C(O2)=C/C3=CC=C(N)C=C3)=O)=C2C=C1	10.1016/j.bbrc.2007.06.162		2.69	0.16	$[^{125}I]$Ono2007-12	
Ono2007-13	IC1=CC(C(/C(O2)=C/C3=CC=C(NC)C=C3)=O)=C2C=C1	10.1016/j.bbrc.2007.06.162		1.24	0.11	$[^{125}I]$Ono2007-12	
Ono2007-14	IC1=CC(C(/C(O2)=C/C3=CC=C(N(C)C)C=C3)=O)=C2C=C1	10.1016/j.bbrc.2007.06.162		6.82	0.48	$[^{125}I]$Ono2007-12	

Compound	SMILES	DOI	K_d / nM	K_i / nM	Error / nM	K_i against	Two-site model?
BZ-OB	F[B-]1(OC(/C=C/C2=CC(C=CC(N(C)C)=C3)=C3O2)=CC(CC(C)C)=[O+]1)F	10.1016/j.bioactmat.2021.10.047	21.56		9.45		
NAP-OB	F[B-]1(OC(/C=C/C2=CC(C=CC(N(C)C)=C3)=C3C=C2)=CC(CC(C)C)=[O+]1)F	10.1016/j.bioactmat.2021.10.047	0.62		0.33		
DBA-SLOH	OCC[N+](C=C1)=C2C(C=CC=C2)=C1/C=C/C(C=C3)=CC=C3N(CCCC)CCCC	10.1016/j.biomaterials.2016.03.047	3090				
DMA-SLOH	CN(C)C1=CC=C(/C=C/C2=C(C=CC=C3)C3=[N+](CCO)C=C2)C=C1	10.1016/j.biomaterials.2016.03.047	7780				
DPA-SLM	C[N+](C=C1)=C2C(C=CC=C2)=C1/C=C/C(C=C3)=CC=C3N(C4=CC=CC=C4)C5=CC=CC=C5	10.1016/j.biomaterials.2016.03.047	17350				
CQ	CCN(CC)C1=CC(OC(C(/C=C/C2=[N+](C)C(C=CC=C3)=C3C=C2)=C4)=O)=C4C=C1	10.1016/j.bios.2017.06.030	86		6.3		
[125I]DMIC	[125I]C1=CC=C(C(/C=C/C2=CC=C(N(C)C)C=C2)=O)C=C1	10.1016/j.bmc.2007.07.052	4.2		1.1		
Ono2009b-08a	O=C(C=C(C1=CC=C(N(C)C)C=C1)O2)C3=C2C=CC(OCCF)=C3	10.1016/j.bmc.2009.01.025		5.3	0.8	[125I]DMFV	
Ono2009b-08b	O=C(C=C(C1=CC=C(N(C)C)C=C1)O2)C3=C2C=CC(OCCOCCF)=C3	10.1016/j.bmc.2009.01.025		14.4	2.5	[125I]DMFV	
Ono2009b-08c	O=C(C=C(C1=CC=C(N(C)C)C=C1)O2)C3=C2C=CC(OCCOCCOCCF)=C3	10.1016/j.bmc.2009.01.025		19.3	4	[125I]DMFV	
Ono2009b-12	O=C(C=C(C1=CC=C(N)C=C1)O2)C3=C2C=CC(OCCF)=C3	10.1016/j.bmc.2009.01.025		234.3	63.5	[125I]DMFV	
Ono2009b-13	O=C(C=C(C1=CC=C(NC)C=C1)O2)C3=C2C=CC(OCCF)=C3	10.1016/j.bmc.2009.01.025		99	11.8	[125I]DMFV	
Ono2009b-15b	O=C(C=C(C1=CC=C(N)C=C1)O2)C3=C2C=CC(OCCOCCF)=C3	10.1016/j.bmc.2009.01.025		321.1	74.4	[125I]DMFV	
Ono2009b-15c	O=C(C=C(C1=CC=C(N)C=C1)O2)C3=C2C=CC(OCCOCCOCCF)=C3	10.1016/j.bmc.2009.01.025		234	60.6	[125I]DMFV	
Ono2009b-17b	O=C(C=C(C1=CC=C(NC)C=C1)O2)C3=C2C=CC(OCCOCCF)=C3	10.1016/j.bmc.2009.01.025		54.5	10.3	[125I]DMFV	

Compound	SMILES	DOI	K_d / nM	K_i / nM	Error / nM	K_i against	Two-site model?
Ono2009b-17c	O=C(C=C(C1=CC=C(NC)C=C1)O2)C3=C2C=CC(OCCOCCOCCF)=C3	10.1016/j.bmc.2009.01.025		45.1	5.8	[^{125}I]DMFV	
Ono2009b-21	FC1=CC(C(C=C(C2=CC=C(N)C=C2)O3)=O)=C3C=C1	10.1016/j.bmc.2009.01.025		260.5	43.3	[^{125}I]DMFV	
Ono2009b-22	FC1=CC(C(C=C(C2=CC=C(NC)C=C2)O3)=O)=C3C=C1	10.1016/j.bmc.2009.01.025		110	47.4	[^{125}I]DMFV	
Ono2009b-23	FC1=CC(C(C=C(C2=CC=C(N(C)C)C=C2)O3)=O)=C3C=C1	10.1016/j.bmc.2009.01.025		73.9	5.3	[^{125}I]DMFV	
PiB	OC1=CC=C(N=C(C2=CC=C(NC)C=C2)S3)C3=C1	10.1016/j.bmc.2009.08.032				ThT	
PP-BTA-1	CN(C)C1=CC=C2C(SC(/C=C(C#N)/C#N)=N2)=C1	10.1016/j.bmc.2009.08.032				ThT	
PP-BTA-2	CN(C)C1=CC=C2C(SC(/C=C/C3=CC=C(/C=C(C#N)/C#N)C=C3)=N2)=C1	10.1016/j.bmc.2009.08.032				ThT	
[125I]TZDM	[125I]C1=CC(SC(C2=CC=C(N(C)C)C=C2)=N3)=C3C=C1	10.1016/j.bmc.2011.03.029	0.45		0.032		
Morais2011a-01E	CN(CCF)C(C=C1)=CC=C1/C=C/C2=NC3=C(S2)C=CC=C3	10.1016/j.bmc.2011.09.065	10670		1550		
Morais2011a-01Z	CN(CCF)C(C=C1)=CC=C1/C=C\C2=NC3=C(S2)C=CC=C3	10.1016/j.bmc.2011.09.065	23960		3570		
Morais2011a-02E	CN(CCF)C(C=C1)=CC=C1/C=C/C2=NC3=C(O2)C=CC=C3	10.1016/j.bmc.2011.09.065	4480		380		
Morais2011a-02Z	CN(CCF)C(C=C1)=CC=C1/C=C\C2=NC3=C(O2)C=CC=C3	10.1016/j.bmc.2011.09.065	5990		560		
Morais2011a-03E	CN(CCF)C(C=C1)=CC=C1/C=C/C2=NC3=C(N2)C=CC=C3	10.1016/j.bmc.2011.09.065	3630		180		
Morais2011a-04E	CN(CCF)C(C=C1)=CC=C1/C=C/C2=NC3=C(N2C)C=CC=C3	10.1016/j.bmc.2011.09.065	5190		570		
Morais2011a-04Z	CN(CCF)C(C=C1)=CC=C1/C=C\C2=NC3=C(N2C)C=CC=C3	10.1016/j.bmc.2011.09.065	3520		170		
ThT	OC1=CC=C([N+](C)=C(C2=CC=C(N(C)C)C=C2)S3)C3=C1	10.1016/j.bmc.2011.09.065	2560		290		

Compound	SMILES	DOI	K_d / nM	K_i / nM	Error / nM	K_i against	Two-site model?
SBIM-1	IC1=CC(NC(/C=C/C2=CC=C(N)C=C2)=N3)=C3C=C1	10.1016/j.bmc.2013.02.054	900		270		
SBIM-2	IC1=CC(NC(/C=C/C2=CC=C(NC)C=C2)=N3)=C3C=C1	10.1016/j.bmc.2013.02.054	420		50		
SBIM-3	IC1=CC(NC(/C=C/C2=CC=C(N(C)C)C=C2)=N3)=C3C=C1	10.1016/j.bmc.2013.02.054	820		10		
BI-1	IC(C=C1)=CC=C1/C=C/C=C/C2=NC3=CC=CC=C3N2CC4=CC=CC=C4	10.1016/j.bmc.2017.10.010	179		59.1		
BI-2	IC(C=C1)=CC=C1/C=C/C=C/C2=NC3=CC=CC=C3N2C	10.1016/j.bmc.2017.10.010	727		227		
BI-3	IC(C=C1)=CC=C1/C=C/C=C/C2=NC3=CC=CC=C3N2CC4=CC=C(OC)C=C4	10.1016/j.bmc.2017.10.010	271		67.3		
[18F]DMFC	FC1=CC=C(C(/C=C/C2=CC=C(N(C)C)C=C2)=O)C=C1	10.1016/j.bmc.2018.05.001	4.47				
[18F]FMC	FC1=CC=C(C(/C=C/C2=CC=C(NC)C=C2)=O)C=C1	10.1016/j.bmc.2018.05.001	6.5				
Lv2018-30	C[n+]1c2c(c(N)c3ccccc31)C(=O)/C(=C/c1cccc3ccccc13)CC2.[I-]	10.1016/j.bmc.2018.08.007	16000				
Resveratrol	Oc1ccc(/C=C/c2cc(O)cc(O)c2)cc1	10.1016/j.bmc.2018.08.007		38500			
Resveratrol	Oc1ccc(/C=C/c2cc(O)cc(O)c2)cc1	10.1016/j.bmc.2018.08.007	38500				
CG	OC(C=C1)=C(C(O)=O)C=C1/N=N/C2=CC=C(C3=CC=C(/N=N/C4=CC(C(O)=O)=C(O)C=C4)C=C3)C=C2	10.1016/j.bmcl.2011.08.003		0.43	0.068	[125I]IMSB	
curcumin	OC1=C(OC)C=C(/C=C/C(/C=C(O)/C=C/C2=CC(OC)=C(O)C=C2)=O)C=C1	10.1016/j.bmcl.2011.08.003		3.57	0.025	[125I]IMSB	
Lee2011-01	O=C(/C=C(O)/C=C/C1=CC=C(OCCF)C(OC)=C1)/C=C/C(C=C2OC)=CC=C2OC	10.1016/j.bmcl.2011.08.003		2.12	0.097	[125I]IMSB	
Lee2011-02	O=C(/C=C(O)/C=C/C1=CC=C(OCCCF)C(OC)=C1)/C=C/C(C=C2OC)=CC=C2OC	10.1016/j.bmcl.2011.08.003		2.69	0.033	[125I]IMSB	

Compound	SMILES	DOI	K_d / nM	K_i / nM	Error / nM	K_i against	Two-site model?
Lee2011-03	OC1=CC=C(/C=C/C(/C=C(O)/C=C/C2=CC=C(OCCOCCF)C(OC)=C2)=O)C=C1OC	10.1016/j.bmcl.2011.08.003		4.65	0.267	[^{125}I]IMSB	
Lee2011-04	OC1=CC=C(/C=C/C(/C=C(O)/C=C/C2=CC=C(OCCOCCOCCF)C(OC)=C2)=O)C=C1OC	10.1016/j.bmcl.2011.08.003		4.44	0.313	[^{125}I]IMSB	
Lee2011-05	COC1=CC=C(/C=C/C(/C=C(O)/C=C/C2=CC=C(OCCOCCF)C(OC)=C2)=O)C=C1OC	10.1016/j.bmcl.2011.08.003		3.01	0.087	[^{125}I]IMSB	
Watanabe2012-05	IC1=CC(/C(C(N2)=O)=C\C3=CC=C(NC)C=C3)=C2C=C1	10.1016/j.bmcl.2012.06.086		5057	158	ThS	
Watanabe2012-06	IC1=CC(/C(C(N2)=O)=C\C3=CC=C(N(C)C)C=C3)=C2C=C1	10.1016/j.bmcl.2012.06.086		4672	748	ThS	
Watanabe2012-13	IC1=CC(C(/C(N2)=C/C3=CC=C(NC)C=C3)=O)=C2C=C1	10.1016/j.bmcl.2012.06.086		116	19	ThS	
Watanabe2012-14	IC1=CC(C(/C(N2)=C/C3=CC=C(N(C)C)C=C3)=O)=C2C=C1	10.1016/j.bmcl.2012.06.086		31	6	ThS	
PT-1	CC(OC(/C=C/C1=CC=C(N(C)C)S1)=C\2)=CC2=C(C#N)/C#N	10.1016/j.bmcl.2015.08.081	54.3				
[125I]Fuchigami2013a-05	[125I]C1=CC=C(C(NC(CCCN(CC)CC)C)=C(C=C(OC)C=C2)C2=N3)C3=C1	10.1016/j.ejmech.2012.12.020	48.4		18.3		
2PmNO-2	CN(C)C1=CC=C(/C=C/C=C/C(O2)=NC(N=CC=C3)=[N+]3[B-]2(F)F)C=C1	10.1016/j.ejmech.2021.113968	164.97		13.47		
4PmNO-2	CN(C)C1=CC=C(/C=C/C=C/C(O2)=NC(C=CN=C3)=[N+]3[B-]2(F)F)C=C1	10.1016/j.ejmech.2021.113968	23.64		1.08		
PdzNO-2	CN(C)C1=CC=C(/C=C/C=C/C(O2)=NC(C=CC=N3)=[N+]3[B-]2(F)F)C=C1	10.1016/j.ejmech.2021.113968	20.45		1.49		
PrzNO-2	CN(C)C1=CC=C(/C=C/C=C/C(O2)=NC(C=NC=C3)=[N+]3[B-]2(F)F)C=C1	10.1016/j.ejmech.2021.113968	>1000				
PyNO-2	CN(C)C1=CC=C(/C=C/C(O2)=NC(C=CC=C3)=[N+]3[B-]2(F)F)C=C1	10.1016/j.ejmech.2021.113968	31.46		5.33		
Yue2022-10a	CN(C)C1=CN=C(C2=CC=C(C=O)C=N2)C=N1	10.1016/j.ejmech.2022.114715	nb				

Compound	SMILES	DOI	K_d / nM	K_i / nM	Error / nM	K_i against	Two-site model?
Yue2022-10b	CN(C)C1=CN=C(C2=CC=C(/C=C/C=O)C=N2)C=N1	10.1016/j.ejmech.2022.114715	36.2		30.4		
Yue2022-1b	CN(C)C1=CC=C(C2=CC=C(/C=C/C=O)C=C2)C=C1	10.1016/j.ejmech.2022.114715	4		0.4		
Yue2022-1c	CN(C)C1=CC=C(C2=CC=C(/C=C/C=C/C=O)C=C2)C=C1	10.1016/j.ejmech.2022.114715	2.5		0.2		
Yue2022-2a	CN(C)C1=NC=C(C2=CC=C(C=O)C=C2)C=C1	10.1016/j.ejmech.2022.114715	nb				
Yue2022-2b	CN(C)C1=NC=C(C2=CC=C(/C=C/C=O)C=C2)C=C1	10.1016/j.ejmech.2022.114715	26.5		4.4		
Yue2022-2c	CN(C)C1=NC=C(C2=CC=C(/C=C/C=C/C=O)C=C2)C=C1	10.1016/j.ejmech.2022.114715	nb				
Yue2022-3a	CN(C)C1=CN=C(C2=CC=C(C=O)C=C2)C=C1	10.1016/j.ejmech.2022.114715	nb				
Yue2022-3b	CN(C)C1=CN=C(C2=CC=C(/C=C/C=O)C=C2)C=C1	10.1016/j.ejmech.2022.114715	37.3		20.6		
Yue2022-4a	CN(C)C1=CC=C(C2=NC=C(C=O)C=C2)C=C1	10.1016/j.ejmech.2022.114715	nb				
Yue2022-4b	CN(C)C1=CC=C(C2=NC=C(/C=C/C=O)C=C2)C=C1	10.1016/j.ejmech.2022.114715	8.5		2.5		
Yue2022-5a	CN(C)C1=NC=C(C2=CC=C(C=O)C=C2)C=N1	10.1016/j.ejmech.2022.114715	nb				
Yue2022-5b	CN(C)C1=NC=C(C2=CC=C(/C=C/C=O)C=C2)C=N1	10.1016/j.ejmech.2022.114715	188.6		75.8		
Yue2022-6a	CN(C)C1=NC=C(C2=CC=C(C=O)C=C2)N=C1	10.1016/j.ejmech.2022.114715	nb				
Yue2022-6b	CN(C)C1=NC=C(C2=CC=C(/C=C/C=O)C=C2)N=C1	10.1016/j.ejmech.2022.114715	337.1		82.7		
Yue2022-7a	CN(C)C1=CC=C(C2=CC=C(C=O)C=N2)C=N1	10.1016/j.ejmech.2022.114715	nb				
Yue2022-7b	CN(C)C1=CC=C(C2=CC=C(/C=C/C=O)C=N2)C=N1	10.1016/j.ejmech.2022.114715	14.2		1.9		
Yue2022-8a	CN(C)C1=CN=C(C2=CC=C(C=O)C=N2)C=C1	10.1016/j.ejmech.2022.114715	nb				
Yue2022-8b	CN(C)C1=CN=C(C2=CC=C(/C=C/C=O)C=N2)C=C1	10.1016/j.ejmech.2022.114715	43.1		5.9		
Yue2022-9a	CN(C)C1=NC=C(C2=CC=C(C=O)C=N2)C=N1	10.1016/j.ejmech.2022.114715	nb				
Yue2022-9b	CN(C)C1=NC=C(C2=CC=C(/C=C/C=O)C=N2)C=N1	10.1016/j.ejmech.2022.114715	153		13.2		

Compound	SMILES	DOI	K_d / nM	K_i / nM	Error / nM	K_i against	Two-site model?
BF-227	[18F]CCOC1=CC=C2C(OC(/C=C/C3=CN=C(N(C)C)S3)=N2)=C1	10.1016/j.ejphar.2009.06.042	1.31				Site 1
BF-227	[18F]CCOC1=CC=C2C(OC(/C=C/C3=CN=C(N(C)C)S3)=N2)=C1	10.1016/j.ejphar.2009.06.042	80				Site 2
Cous-1	O=C1OC2=CC(N(C)C)=CC=C2C=C1/C=C/C(C3=CC=C(C4=CC=C(N(C)C)C=C4)S3)=O	10.1016/j.jlumin.2022.119661	24600				
Cous-4	O=C1OC2=CC(N(CC)CC)=CC=C2C=C1/C=C/C(C3=CC=C(C4=CC=C(N(CC)CC)C=C4)S3)=O	10.1016/j.jlumin.2022.119661	59310				
ThR	O=C1/C(C=C(S(=O)([O-])=O)C2=C1C=CC=C2)=N\N([H])C(C=C3)=CC=C3C4=NC5=CC=C(C)C(S(=O)([O-])=O)=C5S4	10.1016/j.nbd.2007.07.018	2500		400		
ThR	O=C1/C(C=C(S(=O)([O-])=O)C2=C1C=CC=C2)=N\N([H])C(C=C3)=CC=C3C4=NC5=CC=C(C)C(S(=O)([O-])=O)=C5S4	10.1016/j.nbd.2007.07.018		>2000		ThS	
Honson1b	CC1=CC=C(N=C(NC(C2CCN(S(C3=CC=CC4=NSN=C43)(=O)=O)CC2)=O)S5)C5=C1	10.1016/j.nbd.2007.07.018		>2000		ThS	
Honson1c	CC1=CC=C(N=C(NC(C2CN(S(=O)(C3=CC=CC4=NSN=C43)=O)CCC2)=O)S5)C5=C1	10.1016/j.nbd.2007.07.018		>2000		ThS	
Honson1d	COC1=CC=C(N=C(/N=N/C2=CC=C(N(C)C)C=C2)S3)C3=C1	10.1016/j.nbd.2007.07.018		13	2	ThS	
Honson2a	OC1=CC=C2C(NC3=C2C=CC=C3)=C1/N=N\C4=CC=C(C(N)=N)C=C4	10.1016/j.nbd.2007.07.018		200	110	ThS	
Honson2b (Evan's blue)	CC1=CC(C2=CC=C(C(C)=C2)/N=N/C3=CC=C(C4=C3O)C(S(=O)([O-])=O)=CC(S(=O)([O-])=O)=C4N)=CC=C1/N=N/C5=CC=C(C6=C5O)C(S(=O)([O-])=O)=CC(S(=O)([O-])=O)=C6N	10.1016/j.nbd.2007.07.018		180	59	ThS	
Honson3a	CN(CC1)CCN1C2=NC(C=CC=C3)=C3N=C2C(C(OCCCCCC)=O)C#N	10.1016/j.nbd.2007.07.018		150	59	ThS	

Compound	SMILES	DOI	K_d / nM	K_i / nM	Error / nM	K_i against	Two-site model?
Honson3b	COC(C=CC=C1)=C1N2CCN(C3=NC(C=CC=C4)=C4N=C3C(C(OC=C)=O)C#N)CC2	10.1016/j.nbd.2007.07.018		190	39	ThS	
Honson3c	N#CC(C(NCCC1=CC=CC=C1)=O)C2=NC3=C(C=CC=C3)N=C2N4CCCCC4	10.1016/j.nbd.2007.07.018		>2000		ThS	
Honson3d	N#CC(C(OC1CCCCC1)=O)C2=NC3=C(C=CC=C3)N=C2N4CCN(CC)CC4	10.1016/j.nbd.2007.07.018		720	290	ThS	
Honson4a	NC(C=C1)=CC=C1/C(C2=CC=C(N)C=C2)=C3C=CC(C=C/3)=N	10.1016/j.nbd.2007.07.018		110	23	ThS	
Honson4b	CN(C)C(C=C1)=CC=C1/C(C2=CC=C(N(C)C)C=C2)=C3C=C/C(C=C\3)=[N+](C)/C	10.1016/j.nbd.2007.07.018		29	4	ThS	
Honson4c	CN(C)C(C=C1)=CC=C1/C(C2=C(O)C(S(=O)([O-])=O)=CC3=C2C=CC(S(=O)([O-])=O)=C3)=C4C=C/C(C=C\4)=[N+](C)/C	10.1016/j.nbd.2007.07.018		>2000		ThS	
Honson5a	OC1=C(C[C@@](C(C)=O)(O)C[C@@H]2O[C@H]3C[C@H](N)[C@@H](O)[C@H](C)O3)C2=C(O)C(C(C4=C5C=CC=C4OC)=O)=C1C5=O	10.1016/j.nbd.2007.07.018		110	24	ThS	
Honson5b	OC1=C(C[C@](C(CO)=O)(O)C[C@H]2O[C@H]3C[C@@H](N)[C@H](O)[C@@H](C)O3)C2=C(O)C(C(C4=C5C=CC=C4OC)=O)=C1C5=O	10.1016/j.nbd.2007.07.018		320	160	ThS	
Honson6a	O=C1/C(C2=CC=CC=C2N1)=C/C3=CC=C(N(C)C)C=C3	10.1016/j.nbd.2007.07.018		140	23	ThS	
Honson6b	O=C1/C(C2=CC=CC=C2N1)=C/C3=C(OC)C=C(OC)C=C3OC	10.1016/j.nbd.2007.07.018		>2000		ThS	
[125I]Chang 2006-5	CNC(C([125I])=C1)=CC=C1C2=NC3=CC=CC=C3S2	10.1016/j.nucmedbio.2006.06.006	0.44		0.25		
T2	CN(C)C1=CC(OC)=C(/C=C/C=C/C2=NC(C=CC=C3)=C3N=C2)C(OC)=C1	10.1016/j.snb.2023.133406	349.9				
QNO-AD-1	CCCN(C1=CC=CC=C1N=C2/C=C/C3=CC=C(C4=CC=C(N(C)C)C=C4)S3)C2=O	10.1021/acs.analchem.2c01046	16.88		1.726		

Compound	SMILES	DOI	K_d / nM	K_i / nM	Error / nM	K_i against	Two-site model?
QNO-AD-2	CCCN(C1=CC=CC=C1N=C2/C=C/C3=CC=C(C4=CC=C(N(C)C)C=C4)O3)C2=O	10.1021/acs.analchem.2c01046	16.82		1.61		
QNO-AD-3	CN(C1=CC=CC=C1N=C2/C=C/C3=CC=C(C4=CC=C(N(C)C)C=C4)S3)C2=O	10.1021/acs.analchem.2c01046	23.84		1.703		
QNO-AD-4	CN(C1=CC=CC=C1N=C2/C=C/C3=CC=C(C4=CC=C(N(C)C)C=C4)O3)C2=O	10.1021/acs.analchem.2c01046	20.3		2.346		
FPQLN	CN(C)C1=CC=C2C(C=CC(/C=C(C#N)/C3=CC=C(OCCF)C=C3)=N2)=C1	10.1021/acs.analchem.2c02532		36	6	ThT	
FPQXN	CN(C)C1=CC=C2C(N=CC(/C=C(C#N)/C3=CC=C(OCCF)C=C3)=N2)=C1	10.1021/acs.analchem.2c02532		2	0.3	ThT	
MPHN	CN(C)C1=CC=C(/C=C(C#N)/C2=NC(C=CC=C3)=C3N2)C=C1	10.1021/acs.analchem.2c02532		46	18	ThT	
MQXN	CN(C)C1=CC=C2C(N=CC(/C=C(C#N)/C3=NC(C=CC=C4)=C4N3)=N2)=C1	10.1021/acs.analchem.2c02532		5	0.4	ThT	
NPNAN	CN(C)C1=CC=C2C(C=CC(/C=C(C#N)/C3=CC=C([N+]([O-])=O)C=C3)=C2)=C1	10.1021/acs.analchem.2c02532		0.4	0.02	ThT	
NPQLN	CN(C)C1=CC=C2C(C=CC(/C=C(C#N)/C3=CC=C([N+]([O-])=O)C=C3)=N2)=C1	10.1021/acs.analchem.2c02532		1	0.06	ThT	
NPQXN	CN(C)C1=CC=C2C(N=CC(/C=C(C#N)/C3=CC=C([N+]([O-])=O)C=C3)=N2)=C1	10.1021/acs.analchem.2c02532		2	0.1	ThT	
OPQLN	CN(C)C1=CC=C2C(C=CC(/C=C(C#N)/C3=CC=C(O)C=C3)=N2)=C1	10.1021/acs.analchem.2c02532		79	6	ThT	
OPQXN	CN(C)C1=CC=C2C(N=CC(/C=C(C#N)/C3=CC=C(O)C=C3)=N2)=C1	10.1021/acs.analchem.2c02532		15	1	ThT	
ThT	CN(C)C(C=C1)=CC=C1C2=[N+](C)C3=CC=C(O)C=C3S2	10.1021/acs.analchem.2c02532	117		19		
TNAN	CN(C)C1=CC=C2C(C=CC(/C=C(C#N)/C3=NC(C=CC=C4)=C4S3)=C2)=C1	10.1021/acs.analchem.2c02532		0.8	0.04	ThT	
TPHN-1	CN(C)C1=CC=C(/C=C(C#N)/C2=NC(C=CC=C3)=C3S2)C=C1	10.1021/acs.analchem.2c02532		4	0.2	ThT	

Compound	SMILES	DOI	K_d / nM	K_i / nM	Error / nM	K_i against	Two-site model?
TPHN-2	CN(C)C(C=C1)=CC=C1/C=C/C=C(C#N)/C2=NC(C=CC=C3)=C3S2	10.1021/acs.analchem.2c02532		1	0.08	ThT	
TPYN-1	CN(C)C1=CC=C(/C=C(C#N)/C2=NC(C=CC=C3)=C3S2)C=N1	10.1021/acs.analchem.2c02532		26	14	ThT	
TPYN-2	CN(C)C(N=C1)=CC=C1/C=C/C=C(C#N)/C2=NC(C=CC=C3)=C3S2	10.1021/acs.analchem.2c02532		2	0.3	ThT	
TQL2N	CN(C)C1=CC=C2C(C=CC(/C=C(C#N)/C3=NC(C=CC=C4)=C4S3)=C2)=N1	10.1021/acs.analchem.2c02532		11	1	ThT	
TQL6N	CN(C)C1=CC=C2C(C=CC(/C=C(C#N)/C3=NC(C=CC=C4)=C4S3)=N2)=C1	10.1021/acs.analchem.2c02532		1	0.02	ThT	
TQLO	COC1=CC=C2C(C=CC(/C=C(C#N)/C3=NC(C=CC=C4)=C4S3)=N2)=C1	10.1021/acs.analchem.2c02532		120	21	ThT	
TQX	N#C/C(C1=NC(C=CC=C2)=C2S1)=C\C3=NC4=CC=CC=C4N=C3	10.1021/acs.analchem.2c02532		25	8	ThT	
TQX7N	CN(C)C1=CC=C(N=CC(/C=C(C#N)/C2=NC(C=CC=C3)=C3S2)=N4)C4=C1	10.1021/acs.analchem.2c02532		11	1	ThT	
TQXN-1	CN(C)C1=CC=C2C(N=CC(/C=C(C#N)/C3=NC(C=CC=C4)=C4S3)=N2)=C1	10.1021/acs.analchem.2c02532		2	0.9	ThT	
TQXN-2	CN(C)C1=CC=C2C(N=CC(/C=C/C=C(C#N)/C3=NC(C=CC=C4)=C4S3)=N2)=C1	10.1021/acs.analchem.2c02532		0.9	0.03	ThT	
PAD-1	CC(OC(/C=C/C1=CC=C(N(C)C)C=C1)=C\2)=CC2=C(C#N)\C#N	10.1021/acs.analchem.5b00017	58.9				
Fu2016-8a	N#C/C(C#N)=C/C1=CC(C=CC(N(C)CCO)=C2)=C2C=C1	10.1021/acs.analchem.5b04441	>10000				
Fu2016-8b	N#C/C(C#N)=C/C=C/C1=CC(C=CC(N(C)CCO)=C2)=C2C=C1	10.1021/acs.analchem.5b04441	38.5		6.9		
Fu2016-8c	N#C/C(C#N)=C/C=C/C=C/C1=CC(C=CC(N(C)CCO)=C2)=C2C=C1	10.1021/acs.analchem.5b04441	14.5		3.9		
Fu2016-9a	N#C/C(C#N)=C/C1=CC(C=CC(N(CCO)CCO)=C2)=C2C=C1	10.1021/acs.analchem.5b04441	>10000				
Fu2016-9b	N#C/C(C#N)=C/C=C/C1=CC(C=CC(N(CCO)CCO)=C2)=C2C=C1	10.1021/acs.analchem.5b04441	197		43.6		

Compound	SMILES	DOI	K_d / nM	K_i / nM	Error / nM	K_i against	Two-site model?
Fu2016-9c	N#C/C(C#N)=C/C=C/C=C/C1=CC(C=CC(N(CCO)CCO)=C2)=C2C=C1	10.1021/acs.analchem.5b04441	19.9		4.5		
Zhou10a	CN(C)C1=CC=C2C(C=CC(/C=C(C(OCCOCCOCCOC)=O)\C#N)=C2)=C1	10.1021/acs.analchem.8b01712	467.33		136.5		
Zhou10b	CN(C)C1=CC=C2C(C=CC(/C=C/C=C(C(OCCOCCOCCOC)=O)\C#N)=C2)=C1	10.1021/acs.analchem.8b01712	32.93		7.63		
Zhou10c	CN(C)C1=CC=C2C(C=CC(/C=C/C=C/C=C(C(OCCOCCOCCOC)=O)\C#N)=C2)=C1	10.1021/acs.analchem.8b01712	6.49		1.06		
Zhou10d	CN(C)C1=CC=C2C(C=CC(/C=C/C=C/C=C/C=C(C(OCCOCCOCCOC)=O)\C#N)=C2)=C1	10.1021/acs.analchem.8b01712	15.93		3.81		
Zhou11a	CN(C)C1=CC=C2C(C=CC(/C=C(C(OCCO)=O)\C#N)=C2)=C1	10.1021/acs.analchem.8b01712	144.77		4.25		
Zhou11b	CN(C)C1=CC=C2C(C=CC(/C=C/C=C(C(OCCO)=O)\C#N)=C2)=C1	10.1021/acs.analchem.8b01712	46.49		6.1		
Zhou11c	CN(C)C1=CC=C2C(C=CC(/C=C/C=C/C=C(C(OCCO)=O)\C#N)=C2)=C1	10.1021/acs.analchem.8b01712	52.16		9.6		
Zhou11d	CN(C)C1=CC=C2C(C=CC(/C=C/C=C/C=C/C=C(C(OCCO)=O)\C#N)=C2)=C1	10.1021/acs.analchem.8b01712	8.24		1.65		
Zhou12a	CN(C)C1=CC=C2C(C=CC(/C=C(C(OCCOCCO)=O)\C#N)=C2)=C1	10.1021/acs.analchem.8b01712	152.4		1.31		
Zhou12b	CN(C)C1=CC=C2C(C=CC(/C=C/C=C(C(OCCOCCO)=O)\C#N)=C2)=C1	10.1021/acs.analchem.8b01712	62.43		8.4		
Zhou12c	CN(C)C1=CC=C2C(C=CC(/C=C/C=C/C=C(C(OCCOCCO)=O)\C#N)=C2)=C1	10.1021/acs.analchem.8b01712	51.32		11.29		
Zhou12d	CN(C)C1=CC=C2C(C=CC(/C=C/C=C/C=C/C=C(C(OCCOCCO)=O)\C#N)=C2)=C1	10.1021/acs.analchem.8b01712	7.28		3.54		
Zhou13a	CN(C)C1=CC=C2C(C=CC(/C=C(C(OCCOCCOCCO)=O)\C#N)=C2)=C1	10.1021/acs.analchem.8b01712	259.53		50.63		
Zhou13b	CN(C)C1=CC=C2C(C=CC(/C=C/C=C(C(OCCOCCOCCO)=O)\C#N)=C2)=C1	10.1021/acs.analchem.8b01712	49.16		12.6		

Compound	SMILES	DOI	K_d / nM	K_i / nM	Error / nM	K_i against	Two-site model?
Zhou13c	CN(C)C1=CC=C2C(C=CC(/C=C/C=C/C=C(C(OCCOCCOCCO)=O)\C#N)=C2)=C1	10.1021/acs.analchem.8b01712	44.57		10.81		
Zhou13d	CN(C)C1=CC=C2C(C=CC(/C=C/C=C/C=C/C=C(C(OCCOCCOCCO)=O)\C#N)=C2)=C1	10.1021/acs.analchem.8b01712	35.03		12.1		
Zhou7a	CN(C)C1=CC=C2C(C=CC(/C=C(C(OC)=O)\C#N)=C2)=C1	10.1021/acs.analchem.8b01712	359.13		77.21		
Zhou7b	CN(C)C1=CC=C2C(C=CC(/C=C/C=C(C(OC)=O)\C#N)=C2)=C1	10.1021/acs.analchem.8b01712	39.44		12.32		
Zhou7c	CN(C)C1=CC=C2C(C=CC(/C=C/C=C/C=C(C(OC)=O)\C#N)=C2)=C1	10.1021/acs.analchem.8b01712	10.34		1.05		
Zhou7d	CN(C)C1=CC=C2C(C=CC(/C=C/C=C/C=C/C=C(C(OC)=O)\C#N)=C2)=C1	10.1021/acs.analchem.8b01712	8.11		0.65		
Zhou8a	CN(C)C1=CC=C2C(C=CC(/C=C(C(OCCOC)=O)\C#N)=C2)=C1	10.1021/acs.analchem.8b01712	164.5		10.22		
Zhou8b	CN(C)C1=CC=C2C(C=CC(/C=C/C=C(C(OCCOC)=O)\C#N)=C2)=C1	10.1021/acs.analchem.8b01712	30.92		6.38		
Zhou8c	CN(C)C1=CC=C2C(C=CC(/C=C/C=C/C=C(C(OCCOC)=O)\C#N)=C2)=C1	10.1021/acs.analchem.8b01712	30.97		5.37		
Zhou8d	CN(C)C1=CC=C2C(C=CC(/C=C/C=C/C=C/C=C(C(OCCOC)=O)\C#N)=C2)=C1	10.1021/acs.analchem.8b01712	23.1		10.43		
Zhou9a	CN(C)C1=CC=C2C(C=CC(/C=C(C(OCCOCCOC)=O)\C#N)=C2)=C1	10.1021/acs.analchem.8b01712	415.17		93.57		
Zhou9b	CN(C)C1=CC=C2C(C=CC(/C=C/C=C(C(OCCOCCOC)=O)\C#N)=C2)=C1	10.1021/acs.analchem.8b01712	39.99		12.84		
Zhou9c	CN(C)C1=CC=C2C(C=CC(/C=C/C=C/C=C(C(OCCOCCOC)=O)\C#N)=C2)=C1	10.1021/acs.analchem.8b01712	24.59		4.89		
Zhou9d	CN(C)C1=CC=C2C(C=CC(/C=C/C=C/C=C/C=C(C(OCCOCCOC)=O)\C#N)=C2)=C1	10.1021/acs.analchem.8b01712	15.37		3.4		
QAD-1	F[B-]1(F)N2C(C(CCCN3C(C=CC=C4)=C4NCC3)=C5[N+]1=C(C)C=C5C)=C(C)C=	10.1021/acs.bioconjchem.8b00623	27				

Compound	SMILES	DOI	K_d / nM	K_i / nM	Error / nM	K_i against	Two-site model?
	C2/C=C/C6=CC=C(N(C)C)S6						
Chu2015-[18F]46a	O=C1N(CC2=CC=C(OCC[18F])C=C2)C3=CC=CC=C3/C1=C/C=C/C4=CC=C([N+]([O-])=O)C=C4	10.1021/acs.jmedchem.5b00571	271				
Chu2015-05	O=C1NC2=CC=CC=C2/C1=C\C3=CC=C(N(C)C)C=C3	10.1021/acs.jmedchem.5b00571		91.6	18.5	ThT	
Chu2015-06	O=C1NC2=NC=CC=C2/C1=C/C3=CC=C(N(C)C)C=C3	10.1021/acs.jmedchem.5b00571		40	22.3	ThT	
Chu2015-07	O=C1NC2=NC=CC=C2/C1=C\C3=CC=C(N(C)C)C=C3	10.1021/acs.jmedchem.5b00571		94.8	22.4	ThT	
Chu2015-08	O=C1N(C)C2=CC=CC=C2/C1=C\C3=CC=C(N(C)C)C=C3	10.1021/acs.jmedchem.5b00571		155.1	8.8	ThT	
Chu2015-09	O=C1N(CC2=CC=CC=C2)C3=CC=CC=C3/C1=C\C4=CC=C(N(C)C)C=C4	10.1021/acs.jmedchem.5b00571		260.4	78.5	ThT	
Chu2015-10	O=C1N(CC2=CC=C(OC)C=C2)C3=CC=CC=C3/C1=C\C4=CC=C(N(C)C)C=C4	10.1021/acs.jmedchem.5b00571		251.5	52	ThT	
Chu2015-11	O=C1N(CC2=CC=NC=C2)C3=CC=CC=C3/C1=C\C4=CC=C(N(C)C)C=C4	10.1021/acs.jmedchem.5b00571		87.1	34.1	ThT	
Chu2015-12	O=C1N(C(OCC)=O)C2=CC=CC=C2/C1=C\C3=CC=C(N(C)C)C=C3	10.1021/acs.jmedchem.5b00571		139.9	70.1	ThT	
Chu2015-13	O=C1N(CC2=CC=CC=C2)C3=NC=CC=C3/C1=C\C4=CC=C(N(C)C)C=C4	10.1021/acs.jmedchem.5b00571		190.2	84.8	ThT	
Chu2015-14	O=C1N(CC2=CC=C(OC)C=C2)C3=NC=CC=C3/C1=C\C4=CC=C(N(C)C)C=C4	10.1021/acs.jmedchem.5b00571		113.3	2.6	ThT	
Chu2015-18	O=C1NC2=CC=CC=C2/C1=C\C=C\C3=CC=CC=C3	10.1021/acs.jmedchem.5b00571		266.8	136.5	ThT	
Chu2015-19	O=C1NC2=CC=CC=C2/C1=C\C=C\C3=CC=C(OC)C=C3	10.1021/acs.jmedchem.5b00571		125.8	42.6	ThT	
Chu2015-20	O=C1NC2=CC=CC=C2/C1=C/C=C/C3=CC=C(N(C)C)C=C3	10.1021/acs.jmedchem.5b00571		27.6	4.8	ThT	
Chu2015-21	O=C1NC2=NC=CC=C2/C1=C/C=C/C3=CC=C(N(C)C)C=C3	10.1021/acs.jmedchem.5b00571		15.3	5.5	ThT	

Compound	SMILES	DOI	K_d / nM	K_i / nM	Error / nM	K_i against	Two-site model?
Chu2015-22	O=C1NC2=CC=C(O)C=C2/C1=C/C=C/C3=CC=C(N(C)C)C=C3	10.1021/acs.jmedchem.5b00571		37.1	13.6	ThT	
Chu2015-23	O=C1N(CC2=CC=CC=C2)C3=CC=CC=C3/C1=C/C=C/C4=CC=CC=C4	10.1021/acs.jmedchem.5b00571		703.3	214.7	ThT	
Chu2015-24	O=C1N(CC2=CC=CC=C2)C3=CC=CC=C3/C1=C\C=C\C4=CC=C(OC)C=C4	10.1021/acs.jmedchem.5b00571		741	254.6	ThT	
Chu2015-25	O=C1N(C)C2=CC=CC=C2/C1=C\C=C\C3=CC=C(N(C)C)C=C3	10.1021/acs.jmedchem.5b00571		56	14.8	ThT	
Chu2015-26	O=C1N(CC2=CC=CC=C2)C3=CC=CC=C3/C1=C\C=C\C4=CC=C(N(C)C)C=C4	10.1021/acs.jmedchem.5b00571		357.9	161.7	ThT	
Chu2015-27	O=C1N(CC2=CC=C(OC)C=C2)C3=CC=CC=C3/C1=C\C=C\C4=CC=C(N(C)C)C=C4	10.1021/acs.jmedchem.5b00571		129.6	41.4	ThT	
Chu2015-28	O=C1N(CC2=CC=C([N+]([O-])=O)C=C2)C3=CC=CC=C3/C1=C\C=C\C4=CC=C(N(C)C)C=C4	10.1021/acs.jmedchem.5b00571		92.4	21.9	ThT	
Chu2015-29	O=C1N(CC2=CC=NC=C2)C3=CC=CC=C3/C1=C\C=C\C4=CC=C(N(C)C)C=C4	10.1021/acs.jmedchem.5b00571		45.9	19.4	ThT	
Chu2015-30	O=C1N(C(OCC)=O)C2=CC=CC=C2/C1=C\C=C\C3=CC=C(N(C)C)C=C3	10.1021/acs.jmedchem.5b00571		84	35	ThT	
Chu2015-31	O=C1N(CC2=CC=CC=C2)C3=NC=CC=C3/C1=C\C=C\C4=CC=C(N(C)C)C=C4	10.1021/acs.jmedchem.5b00571		85.1	23	ThT	
Chu2015-32	O=C1N(CC2=CC=C(OC)C=C2)C3=NC=CC=C3/C1=C\C=C\C4=CC=C(N(C)C)C=C4	10.1021/acs.jmedchem.5b00571		107	18.9	ThT	
Chu2015-33	O=C1N(CC2=CC=C(N)C=C2)C3=CC=CC=C3/C1=C\C=C\C4=CC=C(N(C)C)C=C4	10.1021/acs.jmedchem.5b00571		98.91	12.1	ThT	
Chu2015-35	O=C1N(C2=CC=CC=C2)C3=CC=CC=C3/C1=C\C4=CC=C(N(C)C)C=C4	10.1021/acs.jmedchem.5b00571		179.2	71.3	ThT	
Chu2015-36	O=C1N(C2=CC=CC=C2)C3=CC=CC=C3/C1=C/C=C/C4=CC=C(N(C)C)C=C4	10.1021/acs.jmedchem.5b00571		286.8	142.2	ThT	
Chu2015-38a	O=C1NC2=CC=CC=C2/C1=C/C3=CC=C([N+]([O-])=O)C=C3	10.1021/acs.jmedchem.5b00571		325.2	73.5	ThT	

Compound	SMILES	DOI	K_d / nM	K_i / nM	Error / nM	K_i against	Two-site model?
Chu2015-38b	O=C1NC2=CC=CC=C2/C1=C\C3=CC=C([N+]([O-])=O)C=C3	10.1021/acs.jmedchem.5b00571		439.2	162.8	ThT	
Chu2015-39a	O=C1N(CC2=CC=CC=C2)C3=CC=CC=C3/C1=C/C4=CC=C([N+]([O-])=O)C=C4	10.1021/acs.jmedchem.5b00571		459.5	49.9	ThT	
Chu2015-39b	O=C1N(CC2=CC=CC=C2)C3=CC=CC=C3/C1=C\C4=CC=C([N+]([O-])=O)C=C4	10.1021/acs.jmedchem.5b00571		694.7	72	ThT	
Chu2015-41a	O=C1NC2=CC=CC=C2/C1=C/C=C/C3=CC=C([N+]([O-])=O)C=C3	10.1021/acs.jmedchem.5b00571		36.2	12.9	ThT	
Chu2015-41b	O=C1NC2=CC=CC=C2/C1=C\C=C\C3=CC=C([N+]([O-])=O)C=C3	10.1021/acs.jmedchem.5b00571		60	19.1	ThT	
Chu2015-42a	O=C1N(CC2=CC=CC=C2)C3=CC=CC=C3/C1=C/C=C/C4=CC=C([N+]([O-])=O)C=C4	10.1021/acs.jmedchem.5b00571		214.2	52.1	ThT	
Chu2015-42b	O=C1N(CC2=CC=CC=C2)C3=CC=CC=C3/C1=C\C=C\C4=CC=C([N+]([O-])=O)C=C4	10.1021/acs.jmedchem.5b00571		235.4	143	ThT	
Chu2015-43a	O=C1N(CC2=CC=C(OC)C=C2)C3=CC=CC=C3/C1=C/C=C/C4=CC=C([N+]([O-])=O)C=C4	10.1021/acs.jmedchem.5b00571		130.8	64.4	ThT	
Chu2015-43b	O=C1N(CC2=CC=C(OC)C=C2)C3=CC=CC=C3/C1=C\C=C\C4=CC=C([N+]([O-])=O)C=C4	10.1021/acs.jmedchem.5b00571		138.5	40.2	ThT	
Chu2015-44a	O=C1N(CC2=CC(OC)=CC=C2)C3=CC=CC=C3/C1=C/C=C/C4=CC=C([N+]([O-])=O)C=C4	10.1021/acs.jmedchem.5b00571		109.7	0.4	ThT	
Chu2015-44b	O=C1N(CC2=CC(OC)=CC=C2)C3=CC=CC=C3/C1=C\C=C\C4=CC=C([N+]([O-])=O)C=C4	10.1021/acs.jmedchem.5b00571		264.8	64.4	ThT	
Chu2015-45a	O=C1N(CC2=CC(OC)=C(OC)C=C2)C3=CC=CC=C3/C1=C/C=C/C4=CC=C([N+]([O-])=O)C=C4	10.1021/acs.jmedchem.5b00571		73.6	27.3	ThT	
Chu2015-45b	O=C1N(CC2=CC(OC)=C(OC)C=C2)C3=CC=CC=C3/C1=C\C=C\C4=CC=C([N+]([O-])=O)C=C4	10.1021/acs.jmedchem.5b00571		129.5	16	ThT	

Compound	SMILES	DOI	K_d / nM	K_i / nM	Error / nM	K_i against	Two-site model?
Chu2015-46a	O=C1N(CC2=CC=C(OCCF)C=C2)C3=CC=CC=C3/C1=C/C=C/C4=CC=C([N+]([O-])=O)C=C4	10.1021/acs.jmedchem.5b00571		142.4	36.9	ThT	
Chu2015-46b	O=C1N(CC2=CC=C(OCCF)C=C2)C3=CC=CC=C3/C1=C\C=C\C4=CC=C([N+]([O-])=O)C=C4	10.1021/acs.jmedchem.5b00571		125.3	40.1	ThT	
Chu2015-47	O=C1NC2=CC=CC=C2/C1=C\C=C\C3=CC=C(N)C=C3	10.1021/acs.jmedchem.5b00571		50.8	18.5	ThT	
ThT	OC1=CC=C([N+](C)=C(C2=CC=C(N(C)C)C=C2)S3)C3=C1	10.1021/acs.jmedchem.5b00571	32				
Fu3a	CN(C)C1=CC(C=CC(/C=C(C#N)/C#N)=C2)=C2C=C1	10.1021/acs.jmedchem.5b00861	44.8		15.3		
Fu3b	CN(C)C1=CC(C=CC(/C=C/C=C(C#N)/C#N)=C2)=C2C=C1	10.1021/acs.jmedchem.5b00861	8.8		1.5		
Fu3c	CN(C)C1=CC(C=CC(/C=C/C=C/C=C(C#N)/C#N)=C2)=C2C=C1	10.1021/acs.jmedchem.5b00861	1.9		1.1		
BTDSB	OC1=CC=C(/C=C/C(C2=NSN=C23)=CC=C3/C=C/C4=CC(C(O)=O)=C(O)C=C4)C=C1C(O)=O	10.1021/acs.jmedchem.8b01681	83		6		
DF-9	OC1=CC=C(/C=C/C2=CC=C(/C=C/C3=CC=C(O)C=C3)C=C2)C=C1	10.1021/acs.jmedchem.8b01681	137		10		
X-34	OC1=CC=C(/C=C/C2=CC=C(/C=C/C3=CC(C(O)=O)=C(O)C=C3)C=C2)C=C1C(O)=O	10.1021/acs.jmedchem.8b01681	149		8		
Zhang2019-3a	C(/C=C/C1=CC=CC=C1)(C2=NSN=C23)=CC=C3/C=C/C4=CC=CC=C4	10.1021/acs.jmedchem.8b01681	66		8		
Zhang2019-3b	OC(C=C1)=CC=C1/C=C/C2=CC=C(C3=NSN=C32)/C=C/C4=CC=CC=C4	10.1021/acs.jmedchem.8b01681	39		1		
Zhang2019-3c	OC(C=C1)=CC=C1/C=C/C(C2=NSN=C23)=CC=C3/C=C/C4=CC=C(O)C=C4	10.1021/acs.jmedchem.8b01681	20		2		
Zhang2019-3d	OC(C=C1)=C(O)C=C1/C=C/C(C2=NSN=C23)=CC=C3/C=C/C4=CC=C(O)C(O)=C4	10.1021/acs.jmedchem.8b01681	39		4		
Zhang2019-3e	OC(C=C1)=C(O)C=C1/C=C/C(C2=NSN=C23)=CC=	10.1021/acs.jmedchem.8b01681	31		4		

Compound	SMILES	DOI	K_d / nM	K_i / nM	Error / nM	K_i against	Two-site model?
	C3/C=C/C4=CC=C(O)C=C4						
Zhang2019-3f	OC1=CC(O)=CC(/C=C/C(C2=NSN=C23)=CC=C3/C=C/C4=CC(O)=CC(O)=C4)=C1	10.1021/acs.jmedchem.8b01681	95		1		
Zhang2019-3g	OC(C=C1)=CC=C1/C=C/C2=CC=C(C3=NSN=C32)/C=C/C4=CC(O)=CC(O)=C4	10.1021/acs.jmedchem.8b01681	73		7		
Zhang2019-3h	OC1=CC(/C=C/C2=CC=C(C3=NSN=C32)/C=C/C4=CC(O)=CC=C4)=CC=C1	10.1021/acs.jmedchem.8b01681	116		10		
Zhang2019-3i	OC(C=C1)=C(O)C=C1/C=C/C(C2=NSN=C23)=CC=C3/C=C/C4=CC=CC(O)=C4	10.1021/acs.jmedchem.8b01681	77		1		
Zhang2019-3j	OC(C=CC=C1)=C1/C=C/C(C2=NSN=C23)=CC=C3/C=C/C4=CC=CC=C4O	10.1021/acs.jmedchem.8b01681	29		5		
Zhang2019-4	OC(C=C1)=CC=C1C(C2=NSN=C23)=CC=C3C4=CC=C(O)C=C4	10.1021/acs.jmedchem.8b01681	511		29		
Zhang2019-5	OC(C=C1)=CC=C1C#CC(C=C2)=CC=C2C(C3=NSN=C34)=CC=C4C5=CC=C(C#CC6=CC=C(O)C=C6)C=C5	10.1021/acs.jmedchem.8b01681	36		3		
Zhang2019-6	OC(C=C1)=CC=C1C(C=C2)=CC=C2C(C3=NSN=C34)=CC=C4C5=CC=C(C6=CC=C(O)C=C6)C=C5	10.1021/acs.jmedchem.8b01681	40		4		
Zhang2019-7	OC(C=C1)=CC=C1C2=CC=C(S2)C(C3=NSN=C34)=CC=C4C5=CC=C(C6=CC=C(O)C=C6)S5	10.1021/acs.jmedchem.8b01681	10		1		
[18F]2FBox	CN(S(C1=C(F)C=CC=C1)(=O)=O)C(C=C2)=CC=C2/C=C/C3=NC4=CC=CC=C4O3	10.1021/acs.molpharmaceut.8b00229	145.3				
[18F]4FBox	CN(S(C1=CC=C(F)C=C1)(=O)=O)C(C=C2)=CC=C2/C=C/C3=NC4=CC=CC=C4O3	10.1021/acs.molpharmaceut.8b00229	7.7				
[18F]BF227	FCCOC1=CC=C2C(OC(/C=C/C3=CN=C(N(C)C)S3)=N2)=C1	10.1021/acs.molpharmaceut.8b00229	0.82		1.08		Site 1
[18F]BF227	FCCOC1=CC=C2C(OC(/C=C/C3=CN=C(N(C)C)S3)=N2)=C1	10.1021/acs.molpharmaceut.8b00229	125.2		29.05		Site 2

Compound	SMILES	DOI	K_d / nM	K_i / nM	Error / nM	K_i against	Two-site model?
RM-27	CN(C)C(C=C1)=CC=C1/C=C/C=C/C2=[N+](CC)C(C=CC=C3)=C3S2	10.1021/acschemneuro.2c00361	94.176		1.36		
RM-28	CN(CCO)C(C=C1)=CC=C1/C=C/C=C/C2=[N+](CC)C(C=CC=C3)=C3S2	10.1021/acschemneuro.2c00361	175.69		4.87		
Seo3g	CN(C)C1=CC(OC)=C(/C=C/C=C/C=C(C#N)/C(OCC2=CC=NC=C2)=O)C(OC)=C1	10.1021/acschemneuro.6b00174	500				
Seo3h	CN(C)C1=CC(OC)=C(/C=C/C=C/C=C(C#N)/C(OCC2=CC=CO2)=O)C(OC)=C1	10.1021/acschemneuro.6b00174	1020				
Dao4a1	CN1C2=CC=CC=C2SC3=C1C=CC(/C=C4C(N(CC(OCC)=O)C(S/4)=S)=O)=C3	10.1021/acschemneuro.6b00380	7.5		0.4		
Dao4a2	CN1C2=CC=CC=C2SC3=C1C=CC(/C=C4C(N(CC(OC(C)(C)C)=O)C(S/4)=S)=O)=C3	10.1021/acschemneuro.6b00380	41.5		3.5		
Dao4b1	CN1C2=CC(Cl)=CC=C2SC3=C1C=CC(/C=C4C(N(CC(OCC)=O)C(S/4)=S)=O)=C3	10.1021/acschemneuro.6b00380	74.2		5.4		
Dao4b2	CN1C2=CC(Cl)=CC=C2SC3=C1C=CC(/C=C4C(N(CC(OC(C)(C)C)=O)C(S/4)=S)=O)=C3	10.1021/acschemneuro.6b00380	117.3		8.7		
Dao4c2	CN1C2=CC(N(CC)CC)=CC=C2SC3=C1C=CC(/C=C4C(N(CC(OC(C)(C)C)=O)C(S/4)=S)=O)=C3	10.1021/acschemneuro.6b00380	237.6		6.2		
Dao5a	CN1C2=CC=CC=C2SC3=C1C=CC(/C=C4C(N(CC(O)=O)C(S/4)=S)=O)=C3	10.1021/acschemneuro.6b00380	146.9		12.6		
Dao5b	CN1C2=CC(Cl)=CC=C2SC3=C1C=CC(/C=C4C(N(CC(O)=O)C(S/4)=S)=O)=C3	10.1021/acschemneuro.6b00380	695.8		14.9		
Dao5c	CN1C2=CC(N(CC)CC)=CC=C2SC3=C1C=CC(/C=C4C(N(CC(O)=O)C(S/4)=S)=O)=C3	10.1021/acschemneuro.6b00380	204.8		20.1		
Dao6	CN1C2=CC=CC=C2SC3=C1C=CC(/C=C4C(N(CC(ON(C)C)=O)C(S/4)=S)=O)=C3	10.1021/acschemneuro.6b00380	96.4		8.3		
Dao7a1	CN1C2=CC=CC=C2SC3=C1C=CC(/C=C4C(N(CCC(OCC)=O)C(S/4)=S)=O)=C3	10.1021/acschemneuro.6b00380	28.5		2.5		

Compound	SMILES	DOI	K_d / nM	K_i / nM	Error / nM	K_i against	Two-site model?
Dao7a2	CN1C2=CC=CC=C2SC3=C1C=CC(/C=C4C(N(CCC(OC(C)(C)C)=O)C(S/4)=S)=O)=C3	10.1021/acschemneuro.6b00380	14.9		1.3		
Dao8	CN1C2=CC=CC=C2SC3=C1C=CC(/C=C4C(N(CCC(O)=O)C(S/4)=S)=O)=C3	10.1021/acschemneuro.6b00380	212.5		24.3		
ThT	OC1=CC=C([N+](C)=C(C2=CC=C(N(C)C)C=C2)S3)C3=C1	10.1021/acschemneuro.6b00380	1942		32.1		
PP-BTA-3	CN(C)C1=CC(N=C(/C=C/C=C(C#N)/C#N)S2)=C2C=C1	10.1021/acschemneuro.6b00450	148		18.1		
PP-BTA-4	CN(C)C1=CC(N=C(/C=C/C=C/C=C(C#N)/C#N)S2)=C2C=C1	10.1021/acschemneuro.6b00450	40.1		3.9		
PP-BTA-5	CN(C)C1=CC(N=C(/C=C/C=C/C=C/C=C(C#N)/C#N)S2)=C2C=C1	10.1021/acschemneuro.6b00450	57.3		8.7		
ThT	OC1=CC=C([N+](C)=C(C2=CC=C(N(C)C)C=C2)S3)C3=C1	10.1021/acschemneuro.6b00450	430.4		33.5		
[125I]ISQ	[125I]C1=CC=C(N=C2)C(N=C2/C=C/C3=CC=C(C=C3)N(C)C)=C1	10.1021/acsmedchemlett.2c00279	8.53				
SQ1	FCCOC1=CC=C(N=CC(/C=C/C2=CC=C(N(C)C)C=C2)=N3)C3=C1	10.1021/acsmedchemlett.2c00279		83.6	59.2	[^{125}I]ISQ	
SQ10	CN(C)C(C=C1)=CC=C1/C=C/C2=NC3=CC=C(N4C=CN=N4)C=C3C=C2	10.1021/acsmedchemlett.2c00279		21	2.2	[^{125}I]ISQ	
SQ11	FCCOC1=CC=C(C=C2)C(N=C2/C=C/C3=CC=C(C=C3)[N+]([O-])=O)=C1	10.1021/acsmedchemlett.2c00279		>1000		[^{125}I]ISQ	
SQ12	FCCOC(C=C1C=C2)=CC=C1N=C2/C=C/C3=CC=C(C=C3)[N+]([O-])=O	10.1021/acsmedchemlett.2c00279		31.7	11.3	[^{125}I]ISQ	
SQ13	CN(C)C(C=C1)=CC=C1/C=C/C2=NC3=CC(OCCOCCOCCF)=CC=C3C=C2	10.1021/acsmedchemlett.2c00279		>1000		[^{125}I]ISQ	
SQ14	CN(C)C(C=C1)=CC=C1/C=C/C2=NC3=CC=C(OCCOCCOCCF)C=C3C=C2	10.1021/acsmedchemlett.2c00279		9.56	1.57	[^{125}I]ISQ	
SQ15	CN(C)C(C=C1)=CC=C1/C=C/C2=NC3=CC(OCC(O)CF)=CC=C3C=C2	10.1021/acsmedchemlett.2c00279		>1000		[^{125}I]ISQ	
SQ16	CN(C)C(C=C1)=CC=C1/C=C/C2=NC3=CC=C(OCC(O)CF)C=C3C=C2	10.1021/acsmedchemlett.2c00279		200	22.8	[^{125}I]ISQ	

Compound	SMILES	DOI	K_d / nM	K_i / nM	Error / nM	K_i against	Two-site model?
SQ2	CN(C)C(C=C1)=CC=C1/C=C/C2=NC3=CC=C(OCCF)C=C3N=C2	10.1021/acsmedchemlett.2c00279		11.7	0.97	[^{125}I]ISQ	
SQ3	CN(C)C(C=C1)=CC=C1/C=C/C2=NC3=CC(OCCF)=CC=C3C=C2	10.1021/acsmedchemlett.2c00279		230	49.1	[^{125}I]ISQ	
SQ4	CN(C)C(C=C1)=CC=C1/C=C/C2=NC3=CC=C(OCCF)C=C3C=C2	10.1021/acsmedchemlett.2c00279		7.69	0.86	[^{125}I]ISQ	
SQ5	CN(C)C(C=C1)=CC=C1/C=C/C2=NC3=CC=CC(OCCF)=C3C=C2	10.1021/acsmedchemlett.2c00279		6.26	3.83	[^{125}I]ISQ	
SQ6	CN(C)C(C=C1)=CC=C1/C=C/C2=NC3=C(OCCF)C=CC=C3C=C2	10.1021/acsmedchemlett.2c00279		>1000		[^{125}I]ISQ	
SQ7	FCCOC1=CC=C2C=CC(/C=C/C3=CC=C(N=C3)N(C)C)=NC2=C1	10.1021/acsmedchemlett.2c00279		>1000		[^{125}I]ISQ	
SQ8	CN(C)C(N=C1)=CC=C1/C=C/C2=NC3=CC=C(OCCF)C=C3C=C2	10.1021/acsmedchemlett.2c00279		47.8	4.84	[^{125}I]ISQ	
SQ9	CN(C)C(C=C1)=CC=C1/C=C/C2=NC3=CC(N4N=NC=C4)=CC=C3C=C2	10.1021/acsmedchemlett.2c00279		>1000		[^{125}I]ISQ	
Zhang17-2	OC1=C(O)C=CC(/C=C/C2=CC=CC=C2)=C1	10.1021/acsomega.7b00535	4300		200		
Zhang17-3	OC1=C(O)C=CC(/C=C/C2=CC(C=CC=C3)=C3C=C2)=C1	10.1021/acsomega.7b00535	2200		400		
Zhang17-4	OC1=C(O)C=CC(/C=C/C2=CC(C=C(C=CC=C3)C3=C4)=C4C=C2)=C1	10.1021/acsomega.7b00535	1600		100		
Zhang17-5	OC1=C(O)C=CC(/C=C/C2=CC(C=CC3=C4C(C=C5)=CC=C3)=C4C5=C2)=C1	10.1021/acsomega.7b00535	1300		100		
Hseih2018b-01	O=C1NC2=CC=CC=C2/C1=C/C=C/C3=CC=C(OC)C=C3	10.1021/acsomega.7b01897		125.8	42.6	ThT	
Hseih2018b-02	O=C1NC2=CC=CC=C2/C1=C/C=C/C3=CC=C(N(C)C)C=C3	10.1021/acsomega.7b01897		27.6	4.8	ThT	
Hseih2018b-03	O=C1NC2=NC=CC=C2/C1=C/C=C/C3=CC=C(N(C)C)C=C3	10.1021/acsomega.7b01897		15.3	5.5	ThT	
Hseih2018b-04	O=C(/C=C/C1=CC=C(OC)C=C1)C2=NC3=CC=CC=C3S2	10.1021/acsomega.7b01897		353	29.7	ThT	

Compound	SMILES	DOI	K_d / nM	K_i / nM	Error / nM	K_i against	Two-site model?
Hseih2018b-05	O=C(/C=C/C1=CC=C(N(C)C)C=C1)C2=NC3=CC=CC=C3S2	10.1021/acsomega.7b01897		906	29.7	ThT	
Hseih2018b-06	O=C(/C=C/C1=CC=C([N+]([O-])=O)C=C1)C2=NC3=CC=CC=C3S2	10.1021/acsomega.7b01897		89	26.9	ThT	
Hseih2018b-07	O=C(/C=C/C1=CC=C(OC)C=C1)C2=NC=CS2	10.1021/acsomega.7b01897		>500		ThT	
Hseih2018b-07	O=C(/C=C/C1=CC=C(OC)C=C1)C2=NC=CS2	10.1021/acsomega.7b01897		>500		ThT	
Hseih2018b-08	O=C(/C=C/C1=CC=C(N(C)C)C=C1)C2=NC=CS2	10.1021/acsomega.7b01897		505	49.5	ThT	
Hseih2018b-09	O=C(/C=C/C1=CC=C([N+]([O-])=O)C=C1)C2=NC=CS2	10.1021/acsomega.7b01897		404	80.6	ThT	
Hseih2018b-10	COC(C=C1)=CC=C1C2=NOC(C3=NC=CS3)=C2	10.1021/acsomega.7b01897		>1000		ThT	
Hseih2018b-10	COC(C=C1)=CC=C1C2=NOC(C3=NC=CS3)=C2	10.1021/acsomega.7b01897		>1000		ThT	
Hseih2018b-11	COC(C=C1)=CC=C1/C=C/C2=NOC(C3=NC=CS3)=C2	10.1021/acsomega.7b01897		91.5	58.7	ThT	
Hseih2018b-12	COC(C=C1)=CC=C1C2=NNC(C3=NC=CS3)=C2	10.1021/acsomega.7b01897		>1000		ThT	
Hseih2018b-12	COC(C=C1)=CC=C1C2=NNC(C3=NC=CS3)=C2	10.1021/acsomega.7b01897		>1000		ThT	
Hseih2018b-13	COC(C=C1)=CC=C1/C=C/C2=NNC(C3=NC=CS3)=C2	10.1021/acsomega.7b01897		327	76.4	ThT	
SLM	C[N+](C=C1)=C2C(C=CC=C2)=C1/C=C/C3=CC4=C(C=C3)N(CCOCCOC)C5=C4C=CC=C5	10.1021/acsomega.8b00475	11400				
[125I]AAU	[125I]C1=CC(C(/C(O2)=C/C3=CC=C(N)C=C3)=O)=C2C=C1	10.1021/bc8003292	4.2				
Maya2009-09	IC1=CC(C(/C(O2)=C/C3=CC=C(OC)C=C3)=O)=C2C=C1	10.1021/bc8003292		2.89	0.42	[^{125}I]AAU	
Maya2009-14	IC1=CC(C(/C(O2)=C/C3=CC=C(O)C=C3)=O)=C2C=C1	10.1021/bc8003292		1.28	0.29	[^{125}I]AAU	
Maya2009-15	IC1=CC(C(/C(O2)=C/C3=CC=C(OCCO)C=C3)=O)=C2C=C1	10.1021/bc8003292		1.05	0.06	[^{125}I]AAU	

Compound	SMILES	DOI	K_d / nM	K_i / nM	Error / nM	K_i against	Two-site model?
Maya2009-16	IC1=CC(C(/C(O2)=C/C3=CC=C(OCCOCCO)C=C3)=O)=C2C=C1	10.1021/bc8003292		3.36	0.29	[^{125}I]AAU	
Maya2009-17	IC1=CC(C(/C(O2)=C/C3=CC=C(OCCOCCOCCO)C=C3)=O)=C2C=C1	10.1021/bc8003292		2.56	0.31	[^{125}I]AAU	
RH1	O=C1/C(SC(N1CC(OCC)=O)=S)=C/C2=CC=C(C3=CC(I)=CC=C3)O2	10.1021/cn200002t		752	128	ThS	
TH1	O=C1/C(NC(N1CC(OCC)=O)=S)=C/C2=CC=C(C3=CC(I)=CC=C3)O2	10.1021/cn200002t		864	147	ThS	
TH2	O=C1/C(NC(N1CC2=CNC=N2)=S)=C/C3=CC=C(C4=CC(I)=CC=C4)O3	10.1021/cn200002t		469	60	ThS	
ANCA-11	O=C(OCCOCCOCCOC)/C(C#N)=C/C1=CC(C=C2)=C(C=C1)C=C2N3CCCCC3	10.1021/cn200018v	1400		200		
Chang14	O=C(OCCOCCOCCOC)/C(C#N)=C/C1=CC(C=C2)=C(C=C1)C=C2N3CCN(C)CC3	10.1021/cn200018v	4600		1300		
Chang15	O=C(OCCOCCOCCOC)/C(C#N)=C/C1=CC(C=C2)=C(C=C1)C=C2N3CCOCC3	10.1021/cn200018v	13800		3100		
Chang16	O=C(OCCOCCOCCOC)/C(C#N)=C/C1=CC(C=C2)=C(C=C1)C=C2NCCN3CCOCC3	10.1021/cn200018v	6700		2100		
Chang17	O=C(OCCOCCOCCOCCOC)/C(C#N)=C/C1=CC(C=C2)=C(C=C1)C=C2N3CCCCC3	10.1021/cn200018v	1600		900		
Chang19	O=C(OCC(CO)O)/C(C#N)=C/C1=CC(C=C2)=C(C=C1)C=C2N3CCCCC3	10.1021/cn200018v	1600		200		
BMAOI 14	OC1=CC=C(/C=C/C(/C(CC2=CN(CCCCCCNC(CCNC(CCNC(CO[C@@H]3CC4=CC[C@]([C@@](CC[C@@H]5[C@@H](CCCC(C)C)C)([H])[C@]5(C)CC6)([H])[C@@]6([H])[C@@]4(C)CC3)=O)=O)=O)N=N2)=C(O)/C=C/C7=CC=C(O)C(OC)=C7)=O)C=C1OC	10.1021/cn200122j	830				
BAP-1	F[B-]1(F)N2C(C=C3[N+]1=CC=C3)=C(C)C=C2/C=C/C4=CC=C(N(C)C)C=C4	10.1021/cn3000058	44.1				

Compound	SMILES	DOI	K_d / nM	K_i / nM	Error / nM	K_i against	Two-site model?
LNS	[H]N1C(S(CC2=NC=CC(OCC(F)(F)[18F])=C2C)=O)=NC3=CC=CC=C31	10.1021/cn500103u	11				
NML	CN1C(S(CC2=NC=CC(OCC(F)(F)[18F])=C2C)=O)=NC3=CC=CC=C31	10.1021/cn500103u	8.2				
IMSB	O=C(O)C1=CC(/C=C/C2=CC=C(/C=C/C3=CC(C(O)=O)=C(OC)C=C3)C(I)=C2)=CC=C1OC	10.1021/ja0167147	0.73				
Cui2a	CN(C)C1=CC=C(/C=C(C#N)/C#N)C=C1	10.1021/ja4052922	1950		189		
Cui2b	CN(C)C1=CC=C(/C=C/C=C(C#N)/C#N)C=C1	10.1021/ja4052922	35.8		2.5		
Cui2c	CN(C)C1=CC=C(/C=C/C=C/C=C(C#N)/C#N)C=C1	10.1021/ja4052922	26.9		3		
Kim05	CN(C1=CC2=C(C=C1)C=C3C(OCC(/C=C(C#N)/C#N)=C3)=C2)C	10.1021/jacs.5b03548	44.6		4.2		
QM-FN-SO3	CN(C)C(C=C1)=CC=C1C2=CC=C(S2)/C=C/C3=C/C(C4=C(N3CCCS(=O)([O-])=O)C=CC=C4)=C(C#N)/C#N	10.1021/jacs.8b12820	170				
[125]ISB	OC1=CC=C(/C=C/C2=CC=C(/C=C/C3=CC(C(O)=O)=C(O)C=C3)C([125I])=C2)C=C1C(O)=O	10.1021/jm010045q	0.15				
[125I]IMSB	O=C(O)C1=CC(/C=C/C2=CC=C(/C=C/C3=CC(C(O)=O)=C(OC)C=C3)C([125I])=C2)=CC=C1OC	10.1021/jm010045q	0.73		0.05		
[125I]TZPI	IC1=CC(SC(C2=CC=C(N3CCN(C)CC3)C=C2)=N4)=C4C=C1	10.1021/jm010045q	0.15		0.03		
CG	OC(C=C1)=C(C(O)=O)C=C1/N=N/C2=CC=C(C3=CC=C(/N=N/C4=CC(C(O)=O)=C(O)C=C4)C=C3)C=C2	10.1021/jm010045q		0.4	0.1	[^{125}I]IMSB	
IMSB	O=C(O)C1=CC(/C=C/C2=CC=C(/C=C/C3=CC(C(O)=O)=C(OC)C=C3)C(I)=C2)=CC=C1OC	10.1021/jm010045q		0.8	0.2	[^{125}I]IMSB	
ThT	CN(C)C(C=C1)=CC=C1C2=[N+](C)C3=CC=C(O)C=C3S2	10.1021/jm010045q		>4000		[^{125}I]IMSB	
TZDM	IC1=CC(SC(C2=CC=C(N(C)C)C=C2)=N3)=C3C=C1	10.1021/jm010045q		>5000		[^{125}I]IMSB	

Compound	SMILES	DOI	K_d / nM	K_i / nM	Error / nM	K_i against	Two-site model?
TZDM	IC1=CC(SC(C2=CC=C(N(C)C)C=C2)=N3)=C3C=C1	10.1021/jm010045q	0.14		0.05		
TZPI	IC1=CC(SC(C2=CC=C(N3CCN(C)CC3)C=C2)=N4)=C4C=C1	10.1021/jm010045q		>2000		$[^{125}I]$IMSB	
Zhuang16a	BrC1=CC(SC(C2=CC=C(N(C)C)C=C2)=N3)=C3C=C1	10.1021/jm010045q		>3000		$[^{125}I]$IMSB	
Zhuang16b	BrC1=CC(SC(C2=CC=C(N3CCN(C)CC3)C=C2)=N4)=C4C=C1	10.1021/jm010045q		>2400		$[^{125}I]$IMSB	
[125I]Ono2005-11	[125I]C1=CC(C(C=C(C2=CC=C(N(C)C)C=C2)O3)=O)=C3C=C1	10.1021/jm050635e	17.4		5.7		
[125I]BOB-4	COc1ccc(OCc2ccc([125I])cc2)cc1	10.1021/jm5004396	0.56		0.18		
[18F]FPPDB	CN(C)c1ccc(/N=N/c2nc3ccc(OCCOCCOCC[18F])cc3s2)cc1	10.1021/ml200230e	45.4				
FPPDB	CN(C)c1ccc(/N=N/c2nc3ccc(OCCOCCOCCF)cc3s2)cc1	10.1021/ml200230e		20		ThS	
ZW800-1C	O=C(O)CCC1=CC=C(C2=C(/C=C/C3=[N+](CCC[N+](C)(C)C)C4=C(C=C(S(=O)(O)=O)C=C4)C3)CCC/C2=C\C=C5N(CCC[N+](C)(C)C)C(C=C(S(=O)(O)=O)C=C6)=C6C\5(C)C)C=C1	10.1038/s41551-023-01003-7	78.9		12.3		
TC	CCN(CC)C1=CC=C2C(OC(C(/C=C/C3=[N+](C)C(C=CC=C4)=C4S3)=C2)=O)=C1	10.1038/srep23668	58.43				
TP	C[N+](C(C=CC=C1)=C1S2)=C2/C=C/C3=CC=C4C(C56)=C3C=CC5=CC=CC6=CC4	10.1038/srep23668	>1000				
Fluselenamyl	FCCOC1=CC(N=C(/C=C\C2=CN=C(N(C)C)N=C2)[Se]3)=C3C=C1	10.1038/srep35636	1.6				
Matsumura07	IC1=CC(SC(/N=N/C2=CC=C(N)C=C2)=N3)=C3C=C1	10.1039/c1md00034a		6.4	0.57	ThS	
Matsumura08	IC1=CC(SC(/N=N/C2=CC=C(NC)C=C2)=N3)=C3C=C1	10.1039/c1md00034a		5.08	0.45	ThS	
Matsumura09	IC1=CC(SC(/N=N/C2=CC=C(N(C)C)C=C2)=N3)=C3C=C1	10.1039/c1md00034a		8.42	0.08	ThS	

Compound	SMILES	DOI	K_d / nM	K_i / nM	Error / nM	K_i against	Two-site model?
SB-13	CNC1=CC=C(/C=C/C2=CC=C(O)C=C2)C=C1	10.1039/c1md00034a		81.2	1.53	ThS	
ThS	C[N+]1=C(C2=CC=C(N(C)C)C=C2)SC3=CC(C4=[N+](C)C5=CC=C(C)C(S(=O)([O-])=O)=C5S4)=CC=C31	10.1039/c1md00034a	1000				
T1	O=C(OCCOCCOCCOCC[N+]1=CC=C(C2=CC(C3=C(C4=C(C)SC(C5=CC=NC=C5)=C4)CCC3)=C(C)S2)C=C1)/C(C#N)=C/C6=CC(C=C7)=C(C=C6)C=C7N8CCCCC8	10.1039/c4cc07656g	1300				
T2	O=C(OCCOCCOCCOCC[N+]1=CC=C(C2=CC(C3=C(C4=C(C)SC(C5=CC=[N+](CCOCCOCCOCCOC(/C(C#N)=C/C6=CC(C=C7)=C(C=C6)C=C7N8CCCCC8)=O)C=C5)=C4)CCC3)=C(C)S2)C=C1)/C(C#N)=C/C9=CC(C=C%10)=C(C=C9)C=C%10N%11CCCCC%11	10.1039/c4cc07656g	1100				
EAU-1	F[B-]1(N2C(C(/C=C/C3=CC=C(N(C)C)C=C3)=C4[N+]1=C(C)C=C4)=CC=C2C)F	10.1039/C4RA07754G	322.8		119.5		
EAU-2	F[B-]1(N2C(C(/C=C/C3=CC=C(N(C)C)C=C3)=C4[N+]1=C(C)C=C4)=CC=C2/C=C/C5=CC=C(OC)C=C5)F	10.1039/C4RA07754G	226.2		127.1		
EAU-3	F[B-]1(N2C(C(/C=C/C3=CC=C(N(C)C)C=C3)=C4[N+]1=C(/C=C/C5=CC=C(OC)C=C5)C=C4)=CC=C2/C=C/C6=CC=C(OC)C=C6)F	10.1039/C4RA07754G	320.1		161.1		
EAU-4	F[B-]1(N2C(C(COCCOCCOC)=C3[N+]1=C(C)C=C3C)=C(C)C=C2/C=C/C4=CC=C(N(C)C)C=C4)F	10.1039/C4RA07754G	48.6		9.7		
EAU-5	F[B-]1(N2C(C(COCCOCCOC)=C3[N+]1=C(/C=C/C4=CC=C(N(C)C)C=C4)C=C3C)=C(C)C=C2/C=C/C5=CC=C(N(C)C)C=C5)F	10.1039/C4RA07754G	96.9		41.9		

Compound	SMILES	DOI	K_d / nM	K_i / nM	Error / nM	K_i against	Two-site model?
BBTO-1	CN(C)C1=CC=C(/C=C2C(NC(NC\2=O)=O)=O)C=C1	10.1039/c5cc03662c		333.1	38.7		
BBTO-2	CN(C)C1=CC=C(/C=C/C=C2C(NC(NC\2=O)=O)=O)C=C1	10.1039/c5cc03662c		165.2	23.1		
BBTO-3	CN(C)C1=CC=C(/C=C/C=C/C=C2C(NC(NC\2=O)=O)=O)C=C1	10.1039/c5cc03662c		22.1	5.6		
BBTOM-2	CN(C)C1=CC=C(/C=C/C=C2C(N(C)C(N(C)C\2=O)=O)=O)C=C1	10.1039/c5cc03662c		119.9	22.3		
BBTOM-3	CN(C)C1=CC=C(/C=C/C=C/C=C2C(N(C)C(N(C)C\2=O)=O)=O)C=C1	10.1039/c5cc03662c		13.7	4.6		
BBTOM-4	CN(C)C1=CC=C(/C=C/C=C/C=C/C=C2C(N(C)C(N(C)C\2=O)=O)=O)C=C1	10.1039/c5cc03662c		28.8	9.3		
BBTOM-5	CN(C)C1=CC=C(/C=C/C=C/C=C/C=C/C=C2C(N(C)C(N(C)C\2=O)=O)=O)C=C1	10.1039/c5cc03662c		21.3	6.3		
BBTS-2	CN(C)C1=CC=C(/C=C/C=C2C(NC(NC\2=S)=O)=S)C=C1	10.1039/c5cc03662c		121	16.7		
BBTS-3	CN(C)C1=CC=C(/C=C/C=C/C=C2C(NC(NC\2=S)=O)=S)C=C1	10.1039/c5cc03662c		14.7	8.4		
DTM-0	CN(C)C1=CC=C(/C=C(C#N)/C#N)S1	10.1039/c5cc06628j	n				
DTM-1	CN(C)C1=CC=C(/C=C/C=C(C#N)/C#N)S1	10.1039/c5cc06628j	811		76		
DTM-2	CN(C)C1=CC=C(/C=C/C=C/C=C(C#N)/C#N)S1	10.1039/c5cc06628j	117		16		
DTM-3	CN(C)C1=CC=C(/C=C/C=C/C=C/C=C(C#N)/C#N)S1	10.1039/c5cc06628j	327		44		
IDP-1	IC1=CC=C(C(/C=C/C2=CC=C(N(C)C)C=C2)=O)C=C1	10.1039/c6ra02710e	12.88		2.02		
IDP-2	IC1=CC=C(C(/C=C/C=C/C2=CC=C(N(C)C)C=C2)=O)C=C1	10.1039/c6ra02710e	37.14		10.8		
IDP-3	IC1=CC=C(C(/C=C/C=C/C=C/C2=CC=C(N(C)C)C=C2)=O)C=C1	10.1039/c6ra02710e	13.35		5.67		

Compound	SMILES	DOI	K_d / nM	K_i / nM	Error / nM	K_i against	Two-site model?
IDP-4	IC1=CC=C(C(/C=C/C=C/C=C/C=C/C2=CC=C(N(C)C)C=C2)=O)C=C1	10.1039/c6ra02710e	16.24		4.78		
MC-1	CC1(C)/C(N(C)C2=C1C=CC=C2)=C\C=C3CCCC(/C=C(C#N)/C#N)=C\3Cl	10.1039/c7cc05056a	59.09				
ICTAD-1	CN(C)C1=CC=C(C2=CC(OC(C(C3=NC4=C(C=CC=C4)S3)=C5)=N)=C5C=C2)C=C1	10.1039/d0sc02060e	3780				
Ferrie2020-61	O=C(COC1=CC=C(C)C(C)=C1)NC2=CC(C3=CC=C(I)C=C3)=NO2	10.1039/D0SC02159H		4.56			
Sanna2021-14	CN(C)C(C=C1)=CC=C1C2=NC3=CC=C(OCCOCCOCCOCCOCCOCCOCC4=CC=C(N=C(C5=CC=C(N(C)C)C=C5)S6)C6=C4)C=C3S2	10.1039/d1sc01263k	1400		1600		
Sanna2021-15	CN(C)C(C=C1)=CC=C1C2=NC3=CC=C(OCCOCCOCCOCCOCCOCCOCCOCCOCCOC4=CC=C(N=C(C5=CC=C(N(C)C)C=C5)S6)C6=C4)C=C3S2	10.1039/d1sc01263k	78		26		
Sanna2021-16	CN(C)C(C=C1)=CC=C1C2=NC3=CC=C(OCCOCCOCCOCCOCCOCCOCCOCCOCCOCCOCCOCCOCCOCCOC4=CC=C(N=C(C5=CC=C(N(C)C)C=C5)S6)C6=C4)C=C3S2	10.1039/d1sc01263k	7		5		
Sanna2021-17	CN(C)C(C=C1)=CC=C1C2=NC3=CC=C(OCCOCCOCCOCCOCCOCCOCCOCCOCCOCCOCCOCCOCCOCCOCCOCCOCCOCCOCCOCCOCCOC4=CC=C(N=C(C5=CC=C(N(C)C)C=C5)S6)C6=C4)C=C3S2	10.1039/d1sc01263k	320		75		
[18F]THK-523	[18F]CCOC1=CC=C(N=C(C2=CC=C(N)C=C2)C=C3)C3=C1	10.1093/brain/awr038	20.7				
Dai2022-7a	CN(C)C1=CC=C(/C=C2CC(C=C(OCCOCCOCC(F)(F)F)C=C3)=C3C\2=O)C=C1	10.1111/cbdd.14162	749		39		
Dai2022-7b	CN(C)C1=CC=C(/C=C2CC(C=C(OCCOCCOC(C(F)(F)F)C(F)(F)F)C=C3)=C3C\2=O)C=C1	10.1111/cbdd.14162	173		19		
Dai2022-7c	CN(C)C1=CC=C(/C=C2CC(C=C(OCCOCCOCC(F)(C(	10.1111/cbdd.14162	595		64		

Compound	SMILES	DOI	K_d / nM	K_i / nM	Error / nM	K_i against	Two-site model?
	F)(F)F)F)C=C3)=C3C\2=O)C=C1						
Dai2022-7d	CN(C)C1=CC=C(/C=C2CC(C=C(OCCOCCOC(C)C(F)(F)F)C=C3)=C3C\2=O)C=C1	10.1111/cbdd.14162	384		56		
Probe3	O=C(N(/C=C/C1=CC=C(N(C)C)C=C1)N=C2)C3=C2OC4=CC(CC(C(O)=O)C#N)=CC=C4O3	10.1155/2018/1651989	350		30		
BF-227	[18F]CCOC1=CC=C2C(OC(/C=C/C3=CN=C(N(C)C)S3)=N2)=C1	10.1177/1536012118796297	15.7		3.1		
BF-227	[18F]CCOC1=CC=C2C(OC(/C=C/C3=CN=C(N(C)C)S3)=N2)=C1	10.1177/1536012118796297		12		[^{3}H]BF-227	
BMB	NC1=CC=C(/C=C/C(C=C2OC)=CC=C2/C=C/C3=CC=C(N)C=C3)C=C1	10.1177/1536012118796297		76		[^{3}H]BF-227	
BTA-1	C1=CC=C(N=C(C2=CC=C(NC)C=C2)S3)C3=C1	10.1177/1536012118796297		146		[^{3}H]BF-227	
Clorgyline	ClC1=CC=C(OCCCN(C)CC#C)C(Cl)=C1	10.1177/1536012118796297		>9000		[^{3}H]BF-227	
Florbetapir	FCCOCCOCCOC1=NC=C(/C=C/C2=CC=C(NC)C=C2)C=C1	10.1177/1536012118796297		12		[^{3}H]BF-227	
Flutafuranol	OC1=CC=C(C=C(C2=CC=C(NC)C=C2F)O3)C3=C1	10.1177/1536012118796297		42		[^{3}H]BF-227	
Flutemetamol	OC1=CC=C(N=C(C2=CC=C(NC)C(F)=C2)S3)C3=C1	10.1177/1536012118796297		65		[^{3}H]BF-227	
Josephson01	CN(C)C1=NC=C(S1)/C=C/C2=NC(C=C3)=C(O2)C=C3OCCF	10.1177/1536012118796297		12		[^{3}H]BF-227	
Josephson02	CN(C)C1=NC=C(S1)/C=C/C2=NC(C=C3)=C(O2)C=C3OCCCF	10.1177/1536012118796297		9.01		[^{3}H]BF-227	
Josephson03	CN(C)C1=NC=C(S1)/C=C/C2=NC(C=C3)=C(O2)C=C3OCCCCF	10.1177/1536012118796297		31		[^{3}H]BF-227	
Josephson04	CN(C)C1=NC=C(S1)/C=C/C2=NC(C=C3)=C(O2)C=C3OCCCCCF	10.1177/1536012118796297		28		[^{3}H]BF-227	
Josephson05	CN(C)C1=NC=C(S1)/C=C/C2=NC(C=C3)=C(O2)C=C3OCCCCCCF	10.1177/1536012118796297		23		[^{3}H]BF-227	
Josephson06	CN(C)C1=NC=C(S1)/C=C/C2=NC(C=C3)=C(O2)C=C3OCCCCCCCF	10.1177/1536012118796297		27		[^{3}H]BF-227	

Compound	SMILES	DOI	K_d / nM	K_i / nM	Error / nM	K_i against	Two-site model?
Josephson07	CN(C)C1=NC=C(S1)/C=C/C2=NC(C=C3)=C(O2)C=C3OCCOCF	10.1177/1536012118796297		11		[^{3}H]BF-227	
Josephson08	CN(C)C1=NC=C(S1)/C=C/C2=NC(C=C3)=C(O2)C=C3OCCOCCOCF	10.1177/1536012118796297		13		[^{3}H]BF-227	
Josephson09	CN(C)C1=NC=C(S1)/C=C/C2=NC(C=C3)=C(O2)C=C3OCCOCCOCCOCF	10.1177/1536012118796297		26		[^{3}H]BF-227	
Josephson10	CN(C)C1=NC=C(S1)/C=C/C2=NC(C=C3)=C(O2)C=C3OCCOCCOCCOCCOCF	10.1177/1536012118796297		18		[^{3}H]BF-227	
Josephson11	IC1=CC(OC(/C=C/C2=CN=C(N(C)C)S2)=N3)=C3C=C1	10.1177/1536012118796297		223		[^{3}H]BF-227	
Josephson12	CN(C)C1=NC=C(S1)/C=C/C2=NC3=C(O2)C=CC(I)=C3	10.1177/1536012118796297		96		[^{3}H]BF-227	
Josephson13	FC1=CC(OC(/C=C/C2=CC=CC=C2)=N3)=C3C=C1	10.1177/1536012118796297		1247		[^{3}H]BF-227	
Josephson14	FC1=CC2=C(OC(/C=C/C3=CC=CC=C3)=N2)C=C1	10.1177/1536012118796297		528		[^{3}H]BF-227	
Josephson15	FC1=CC(OC(/C=C/C2=CN=CS2)=N3)=C3C=C1	10.1177/1536012118796297		1446		[^{3}H]BF-227	
PiB	OC1=CC=C2C(SC(C3=CC=C(NC)C=C3)=N2)=C1	10.1177/1536012118796297		77		[^{3}H]BF-227	
RO-16-6491	ClC1=CC=C(C(NCCN)=O)C=C1	10.1177/1536012118796297		>9000		[^{3}H]BF-227	
ThS	C[N+]1=C(C2=CC=C(N(C)C)C=C2)SC3=CC(C4=[N+](C)C5=CC=C(C)C(S(=O)([O-])=O)=C5S4)=CC=C31	10.1177/1536012118796297		2150		[^{3}H]BF-227	
[125I]SIL23	[125I]/C=C/COC1=CC(SC2=CC([N+]([O-])=O)=CC=C2N3)=C3C=C1	10.1371/journal.pone.0055031	635				
SIL22	BrC1=CC(SC2=CC([N+]([O-])=O)=CC=C2N3)=C3C=C1	10.1371/journal.pone.0055031		102		[^{125}I]SIL2^3	
SIL26	FCCOC1=CC(SC2=CC([N+]([O-])=O)=CC=C2N3)=C3C=C1	10.1371/journal.pone.0055031		103		[^{125}I]SIL2^3	
SIL3B	O=[N+](C1=CC=C(N2)C(SC3=C2C=CC=C3)=C1)[O-]	10.1371/journal.pone.0055031		71.5		[^{125}I]SIL2^3	

Compound	SMILES	DOI	K_d / nM	K_i / nM	Error / nM	K_i against	Two-site model?
SIL5	COC1=CC(SC2=CC([N+]([O-])=O)=CC=C2N3)=C3C=C1	10.1371/journal.pone.0055031		110		[^{125}I]SIL2[3]	
[125I]BF-180	CNC(C=C1)=CC=C1/C=C/C2=NC3=C(O2)C=CC([125I])=C3	10.1385/JMN:24:2:247	10.6		1.5		
BF-133	FC1=CC=C(OC(/C=C/C2=CC=C(N(C)C)C=C2)=N3)C3=C1	10.1385/JMN:24:2:247		3.4	0.73	[^{125}I]BF-180	
BF-140	FC1=CC=C(OC(/C=C/C2=CC=C(N)C=C2)=N3)C3=C1	10.1385/JMN:24:2:247		2.1	0.18	[^{125}I]BF-180	
BF-145	FC1=CC=C(OC(/C=C/C2=CC=C(NC)C=C2)=N3)C3=C1	10.1385/JMN:24:2:247		4.5	1.9	[^{125}I]BF-180	
BF-151	FC1=CC=C(OC(C2=CC3=CC=C(N(C)C)C=C3C=C2)=N4)C4=C1	10.1385/JMN:24:2:247		5.3	0.56	[^{125}I]BF-180	
[3H]PiB	OC1=CC=C(N=C(C2=CC=C(N([3H])C)C=C2)S3)C3=C1	10.1523/JNEUROSCI.0630-07.2007	0.71				Site 1
[3H]PiB	OC1=CC=C(N=C(C2=CC=C(N([3H])C)C=C2)S3)C3=C1	10.1523/JNEUROSCI.0630-07.2007	19.8				Site 2
BF-126	CN(C)C(C=C1)=CC=C1/C=C/C2=NC3=C(N2)C=CC=C3	10.1523/JNEUROSCI.1738-05.2005		1.2	0.68	[^{125}I]BF-180	
BF-158	CNC(C=C1)=CC=C1C2=NC3=CC=CC=C3C=C2	10.1523/JNEUROSCI.1738-05.2005		>5000		[^{125}I]BF-180	
BF-170	NC(C=C1)=CC=C1C2=NC3=CC=CC=C3C=C2	10.1523/JNEUROSCI.1738-05.2005		>5000		[^{125}I]BF-180	
[3H]BTA-1	C1=CC=C(N=C(C2=CC=C(NC[3H])C=C2)S3)C3=C1	10.1523/jneurosci.23-06-02086.2003	2.8		0.35		
BF-124	CCN(CC)C(C=C1)=CC=C1/C=C/C2=NC3=C(S2)C=CC=C3	10.1523/JNEUROSCI.4456-03.2004		10.9	2.2	[^{125}I]BF-180	
BF-125	CCN(CC)C(C=C1)=CC=C1/C=C/C2=NC3=C(O2)C=CC=C3	10.1523/JNEUROSCI.4456-03.2004		4.9	1.9	[^{125}I]BF-180	
BF-148	FC1=CC(OC(/C=C/C2=CC=C(N(C)C)C=C2)=N3)=C3C=C1	10.1523/JNEUROSCI.4456-03.2004		4.2	2.5	[^{125}I]BF-180	
BF-164	NC(C=C1)=CC=C1/C=C/C2=NC3=C(O2)C=CC=C3	10.1523/JNEUROSCI.4456-03.2004		0.38	0.4	[^{125}I]BF-180	

Compound	SMILES	DOI	K_d / nM	K_i / nM	Error / nM	K_i against	Two-site model?
BF-165	CNC(C=C1)=CC=C1/C=C/C2=NC3=C(O2)C=C(O)C=C3	10.1523/JNEUROSCI.4456-03.2004		1.8	0.2	[^{125}I]BF-180	
BF-168	CNC(C=C1)=CC=C1/C=C/C2=NC3=C(O2)C=C(OCCF)C=C3	10.1523/JNEUROSCI.4456-03.2004		6.4	1	[^{125}I]BF-180	
BF-169	CNC(C=C1)=CC=C1/C=C/C2=NC3=C(O2)C=CC=C3	10.1523/JNEUROSCI.4456-03.2004		7.1	1.2	[^{125}I]BF-180	
BF-180	CNC(C=C1)=CC=C1/C=C/C2=NC3=C(O2)C=CC(I)=C3	10.1523/JNEUROSCI.4456-03.2004	10.6		1.5		
BF-191	ClC(C=C1)=CC=C1/C=C/C2=NC3=C(O2)C=CC=C3	10.1523/JNEUROSCI.4456-03.2004		>5000		[^{125}I]BF-180	
BF-208	FC(C=C1)=CC=C1/C=C/C2=NC3=C(O2)C=CC=C3	10.1523/JNEUROSCI.4456-03.2004		>5000		[^{125}I]BF-180	
N-282	CN(C)C(C=C1)=CC=C1/C=C/C2=NC3=C(O2)C=CC=C3	10.1523/JNEUROSCI.4456-03.2004		4.3	1.4	[^{125}I]BF-180	
BAP-2	F[B-]1(F)N2C(C=C3[N+]1=CC=C3)=C(C)C=C2/C=C/C4=CC=C(N(C)C)S4	10.2310/7290.2013.00049	55				
BAP-3	F[B-]1(F)N2C(C=C3[N+]1=CC=C3)=C(C)C=C2/C=C/C4=CC=C(N(C)C)O4	10.2310/7290.2013.00049	149				
BAP-4	F[B-]1(F)N2C(C=C3[N+]1=CC=C3)=C(C)C=C2/C=C/C4=CC=C(C5=CC=C(N(C)C)C=C5)S4	10.2310/7290.2013.00049	27				
BAP-5	F[B-]1(F)N2C(C=C3[N+]1=CC=C3)=C(C)C=C2/C=C/C4=CC=C(C5=CC=C(N(C)C)C=C5)O4	10.2310/7290.2013.00049	18				
BF-227	[18F]CCOC1=CC=C2C(OC(/C=C/C3=CN=C(N(C)C)S3)=N2)=C1	10.2967/jnumed.106.037556		4.3	1.5	[^{125}I]BF-180	
THK-5105	[18F]CC(O)COC1=CC=C(N=C(C2=CC=C(N(C)C)C=C2)C=C3)C3=C1	10.2967/jnumed.112.117341	35.9				
[125I]DRM106	[125I]C1=CN2C(C=C1)=NC(C3=CC=C(C4=NNC=C4)C=C3)=C2	10.2967/jnumed.114.146944	10.1		5.1		

Compound	SMILES	DOI	K_d / nM	K_i / nM	Error / nM	K_i against	Two-site model?
[125I]DRM106	[125I]C1=CN2C(C=C1)=NC(C3=CC=C(C4=NNC=C4)C=C3)=C2	10.2967/jnumed.114.146944	2890		344.5		
Astemizole	FC(C=C1)=CC=C1CN2C(NC3CCN(CCC4=CC=C(OC)C=C4)CC3)=NC5=CC=CC=C52	10.3233/JAD-2010-1262	2.1		0.1		
[125I]TZDM	IC1=CC(SC(C2=CC=C(N(C)C)C=C2)=N3)=C3C=C1	10.5012/bkcs.2008.29.9.1765	0.13				
AH-2	CN(C)C1=CC=C2C(C=CC(C3=[N+](C)C(C=CC=C4)=C4S3)=C2)=C1	10.7150/thno.68743	227				
PyrPEG	CNC1=CC=C2C(OC(C3=NC=C(C4=CC5=C(C=C(OCCOCCOC)C=C5)O4)N=C3)=C2)=C1	acschemneuro.0c00211	63.8				
